# Base-Promoted Annulation of Amidoximes with Alkynes: Simple Access to 2,4-Disubstituted Imidazoles

**DOI:** 10.3390/molecules25163621

**Published:** 2020-08-09

**Authors:** Hina Mehmood, Muhammad Asif Iqbal, Le Lu, Ruimao Hua

**Affiliations:** Key Laboratory of Organic Optoelectronics & Molecular Engineering of Ministry of Education, Department of Chemistry, Tsinghua University, Beijing 100084, China; hinamehmood123@gmail.com (H.M.); ykb15@mails.tsinghua.edu.cn (M.A.I.); lul18@mails.tsinghua.edu.cn (L.L.)

**Keywords:** base-promoted, annulation, amidoximes, alkynes, 2,4-disubstituted imidazoles

## Abstract

An efficient construction of imidazole ring by a Cs_2_CO_3_-promoted annulation of amidoximes with terminal alkynes in DMSO has been developed. This protocol provides a simple synthetic route with high atom-utilization for the synthesis of 2,4-disubstituted imidazoles in good yields under transition-metal-free and ligand-free conditions. Internal alkynes can also undergo the annulation to give 2,4,5-trisubstituted imidazoles.

## 1. Introduction

**Figure 1:**
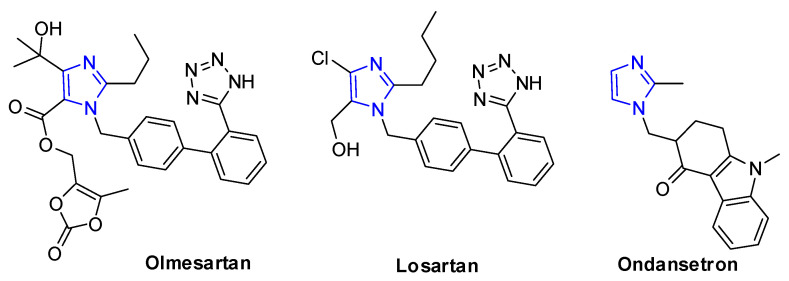
Examples of commercial drugs with imidazole skeleton.

Imidazole ring is the important class of nitrogen-heterocyclic structural motif that has been found in several commercial drugs, such as olmesartan [1,2,3,4] and losartan [5,6,7], for the treatment of hypertension, and ondansetron [8,9,10,11] for reducing nausea and emesis, which have become the best-selling five-membered ring heterocyclic pharmaceuticals [12] (Figure 1). Molecules having this nitrogen-heterocyclic structure often exhibit important and interesting physiological and biological activities [13,14,15]. In addition, imidazoles have been also used as ligands in organometallic complexes [16,17]. Therefore, there has been increasing interest in the developments of efficient methodologies for the construction of imidazole ring [18,19,20].

**Scheme 1:**
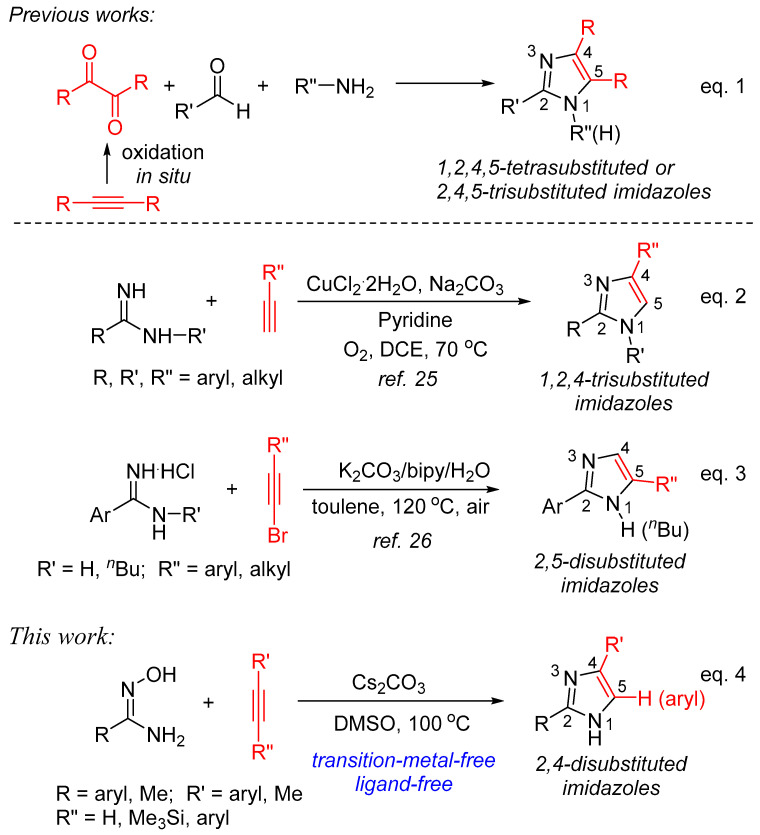
Synthesis of diverse position-substituted imidazoles from alkynes.

Among them, the construction of imidazole starting from alkyne as one of the reactants has been well developed. As depicted in Scheme 1, one of the classic approaches to synthesize 1,2,4,5-tetrasubstituted or 2,4,5-trisubstituted imidazoles is the three-component cyclization of α-diketone, aldehyde, and amine or ammonia sources catalyzed by transition metal complexes or under acidic conditions (Equation (1)). Recently, this protocol has been evolved using internal alkynes as starting materials via generating 1,2-diketones in situ by oxidation reaction [21,22,23,24]. On the other hand, the formal [3+2] annulation of substituted amidines with alkyne forming imidazole ring usually shows high atom-utilization, and two representative procedures are concluded in Scheme 1. One of the known procedures is the annulation of amidines with terminal alkynes catalyzed by CuCl_2_·2H_2_O in pyridine in the presence of Na_2_CO_3_ under atmospheric oxygen to afford 1,2,4-trisubstituted imidazoles in modest to good yields (Equation (2)) [25]. The other involves the cyclocondensation of amidine hydrochlorides with bromoacetylenes promoted by K_2_CO_3_ under air in the presence of 2,2′-bipyridine and water affording various 2,5-disubstituted imidazoles in good yields (Equation (3)) [26]. It is readily apparent to find that, in addition to the requirement of CuCl_2_·2H_2_O and/or ligand (pyridine and 2,2′-bipyridine), in both cases, inorganic bases were used as the additives, indicating that base is the key promoter to realize the formation of imidazoles. Therefore, in continuation of our interest in developing alkyne annulation in the synthesis of nitrogen-heterocyclic compounds [27,28,29,30] and base/DMSO-promoted C-N bond formation [31,32,33,34], we decided to explore the possibility of constructing an imidazole ring with the use of inorganic bases as the promoters, without the use of ligands under transition-metal-free conditions. In this paper, we would like to report a new, simple and efficient procedure to afford a variety of 2,4-disubstituted imidazoles starting with amidoximes and terminal alkynes in the presence of Cs_2_CO_3_ in DMSO (Equation (4)) [35].

## 2. Results and Discussion

We firstly examined the reaction of benzamidoxime (*N*′-hydroxybenzimidamide) (**1a**) with phenyl acetylene (**2a**, 2.0 equivalent) in the presence of Na_2_CO_3_ (4.0 equivalent) in DMSO at 100 °C for 24 h; fortunately, 2,4-diphenylimidazole (**3aa**) could be isolated from the reaction mixture in a 10% yield (Table 1, entry 1). The structure of **3aa** was characterized by its ^1^H- and ^13^C-NMR spectral data, which are the same as the reported ones. In addition, **3aa** was recrystallized in a mixed solvents of petroleum/EtOAc/EtOH as white crystals, its X-ray diffraction studies confirm the structure unambiguously [36].

Several other inorganic bases, such as K_2_CO_3_, KOH, KO*^t^*Bu and Cs_2_CO_3_, were then examined, and **3aa** could be obtained in a 34~75% yield (Table 1, entries 2–5). These results indicate that, in DMSO, Cs_2_CO_3_ is the best base for the present transformation, thus, the influence of other solvents and the amounts of **1a** and Cs_2_CO_3_ were investigated. As shown in entries 6–8, when THF, 1,4-dioxane and DMF were used as solvents to replace DMSO, the yields of **3aa** were decreased significantly. In addition, decreasing amounts of **2a** (from 2.0 equivalent to 1.5 equivalent or 1.0 equivalent) resulted in the considerable decrease of yields (Table 1, entries 9–10). Although the use of 2.5. of Cs_2_CO_3_ also gave results similar to those in entry 5 (Table 1, entry 11 vs. entry 5), the yields of **3aa** were reduced when 1.0 equivalent or 0.5. of Cs_2_CO_3_ were used (Table 1, entries 12–13). In addition, as discussed above, the base is the key promoter to promote the formation of imidazoles, the absence of Cs_2_CO_3_ led to no **3aa** formation at all (Table 1, entry 14).

**Scheme 2:**
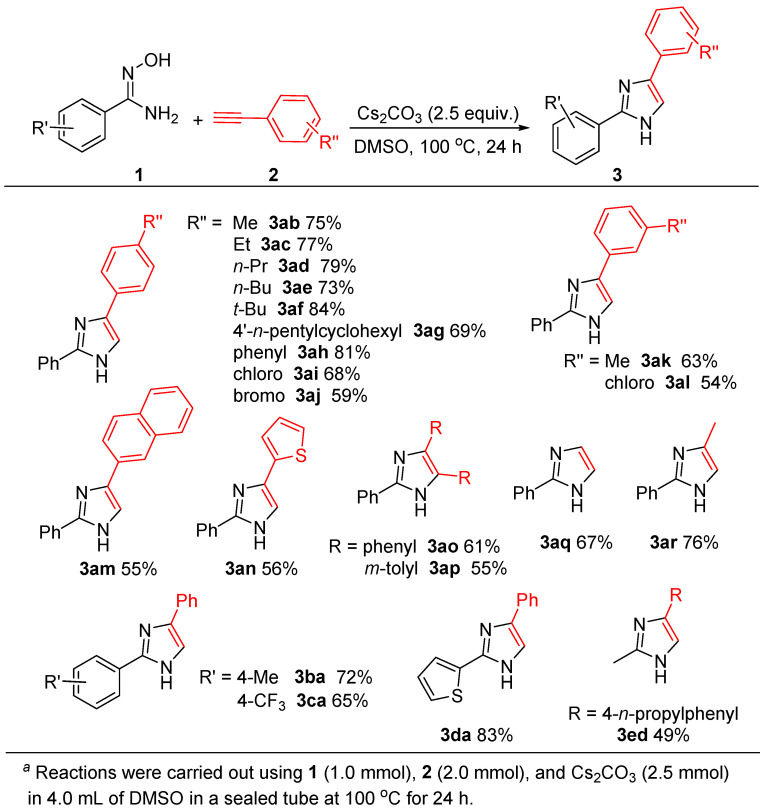
Substrate scope of imidazole synthesis *^a^*.

With the optimized conditions established (Table 1, entry 11), we then investigated the scope and generality of the imidazole formation with the use of various alkynes bearing electron-donating and electron-withdrawing groups, as well as several amidoximes, and the obtained results are concluded in Scheme 2. The reactions of **1a** with various aromatic terminal alkynes bearing electron-donating groups and electron-withdrawing groups could occur smoothly, to give the corresponding imidazoles in moderate to good yields. It was noted that *para*-alkyl-substituted aromatic alkynes (R″ = Me, **2b**; Et, **2c**; *n*-Pr, **2d**; *n*-Bu, **2e**; *t*-Bu, **2f**; 4′-*n*-pentylcyclohexyl, **2g**) showed high reactivity to produce the desired products (**3ab** ~ **3ag**) in 69–84% yields. *para*-Phenyl phenyl acetylene (**2h**) reacted with **1a** afforded **3ah** in a high yield (81%). The reaction of *para*-chlorophenyl acetylene (**2i**), an electron-poor alkyne, with **1a** gave the product **3ai** in a moderate yield (68%). In addition, the reaction of **1a** with *para*-bromophenyl acetylene (**2j**) afford **3aj** in a 59% yield. These results were apparent that the electron-donating group on aromatic terminal alkynes would benefit the formation of imidazoles.

When *meta*-substituted aromatic terminal alkynes were used, the reactions gave slight decrease in yields as compared to *para*-substituted ones. For example, the reactions of **1a** with (3-methylphenyl)ethyne (**2k**), or with (3-chlorophenyl)ethyne (**2l**) gave **3ak** in a 63% yield (vs. **3ab** 75%) and **3al** in a 54% yield (vs. **3ag** 68%), respectively. It was noted that, in these cases, alkyne bearing electron-donating group also show relatively high reactivity (**3ak** vs. **3al**), similar to the results as R″ at *para*-position.

In addition, the present reaction conditions are also suitable for the annulation of **1a** with 2-naphthylacetylene (**2m**), and with 2-thienylacetylene (**2n**), a heteroaromatic terminal alkyne, afforded **3am** and **3an** in 55% and 56% yields, respectively.

Moreover, under the similar conditions, internal alkynes can also undergo the cyclocondensation with **1a** to produce the corresponding 2,4,5-trisubstituted imidazoles. For instance, the reactions of **1a** with 1,2-diphenyacetylene (**2o**), or with 1,2-bis(m-tolyl)acetylene (**2p**) gave **3ao** and **3ap** in 61% and 55% yields.

Interestingly, when 1-(trimethylsilyl)acetylene (**2q**) and 1-methyl-2-trimethylsilylacetylene (**2r**) were employed, the reactions with **1a** produced 2-phenylimidazole (**3aq**, 67%) and 2-phenyl-4- methylimidazole (**3ar**, 76%), indicating that desilylation took place smoothly under the used basic conditions.

On the other hand, the substituent (R′) effect on the aryl group of **1** was also investigated, and the results from two representative examples with the use of either electron-donating group (*para*-Me, **1b**) or electron-withdrawing group (*para*-CF_3_, **1c**) were reported. As shown in Scheme 2, the corresponding products of **3ba** and **3ca** could be obtained in 72% and 65% yields, respectively. Again, the electron-donating group is favorable for the formation of imidazole ring.

Additionally, the present reaction conditions could be applied to heteroarylamidoximes and alkylamidoximes. For example, the reaction between 2-thienylamidoxime (**1d**) and **2a** afford **3da** in a 83% yield, and the reaction of acetamidoxime (**1e**) with **2d** produced the expected product of **3ed** in a 49% yield.

It is worth noting that the present reaction conditions are tolerant to C(*sp*^2^)-Cl and C(*sp*^2^)-Br bonds, the obtained products bearing C(*sp*^2^)-X bonds have a highly potential application in organic synthesis via their cross-coupling reactions.

**Scheme 3:**
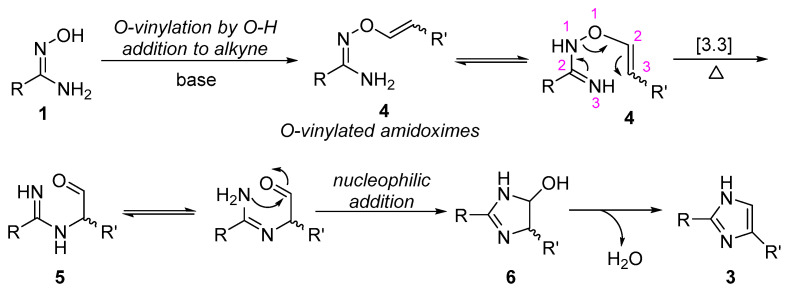
Proposed mechanism for the formation of imidazoles.

Amidoximes have been well known to be the useful building blocks for the construction of nitrogen-heterocyclic compounds, and the five-membered nitrogen-heterocyclic compounds formation is usually proposed to involve the step of N-O bond cleavage via 3,3-sigmatropic rearrangement [37,38,39]. Therefore, on the basis of our results and the known chemistry of amidoximes, a possible reaction mechanism for the formation of imidazole ring is shown in Scheme 3. It involves the regioselective nucleophilic addition of O-H bond to alkyne under basic conditions, giving an *o*-vinylated amidoxime (**4**), which undergoes sequential 3,3-sigmatropic rearrangement forming intermediate 5, and intramolecular nucleophilic addition of nitrogen atom to aldehyde affording five-membered heterocyclic intermediate **6**, followed by dehydration to form imidazole ring.

**Figure 2:**
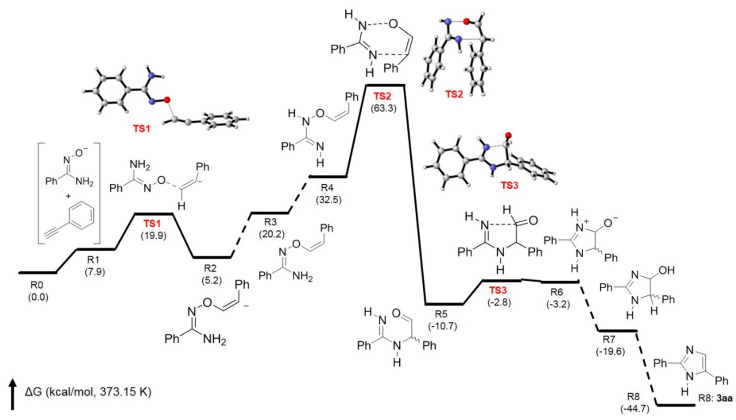
Free energy profile for the base-promoted annulation of **1a** with **2a** affording **3aa**.

In order to support the proposed mechanism, a theoretical calculation was conducted by using the quantum chemistry program Gaussian 16 [40], and all structures were optimized by using M06-2X Minnesota functional with the 6-31G(d,p) basis set [41]. Figure 2 shows the free energy changes in the base-promoted annulation of **1a** with **2a** forming **3aa** with the transition states for the formation of key intermediates **4**–**6**. It clearly indicates that the transition state for the formation of intermediates **4** (from TS1) and **6** (from TS3) can be found with low activation energies. The key step for the formation of imidazole ring is the 3,3-sigmatropic rearrangement to give intermediate **5**. The transition state (TS2) between R4 (intermediate **4**) and R5 (intermediate **5**) is not very high in activation energy, with 30.8 kcal/mol (TS2-R4), and the Gibbs free energy change in this step is −43.2 kcal/mol. These results have confirmed that at 100 °C (373.15 K, reaction temperature), 3,3-sigmatropic rearrangement can occur smoothly to construct imidazole ring as the proposed routes shown in Scheme 3.

## 3. Materials and Methods

### 3.1. General Methods

All commercial reagents are analytically pure and used directly without further purification. Nuclear magnetic resonance (NMR) spectra were recorded on an ECA-400 spectrometer (JEOL, Tokyo, Japan) using DMSO-*d*_6_ as solvent at 298 K. ^1^H-NMR (400 MHz) chemical shifts (δ) were referenced to internal standard TMS (for 1H, *δ* = 0.00 ppm). ^13^C-NMR (100 MHz) chemical shifts were referenced to internal solvent DMSO-*d*_6_ (for ^13^C, *δ* = 39.52 ppm). Mass spectra (MS) were obtained on a GC-MS-QP2010S (Shimadzu, Tokyo, Japan), and the high-resolution mass spectra (HRMS) with electron spray ionization (ESI) were obtained with a micrOTOF-Q spectrometer (Agilent, California, CA, USA). Single crystals of 3aa were obtained by slow evaporation of their solution in a mixture solvents of petroleum, ethyl acetate and EtOH.

### 3.2. Typical Experimental Procedure for the Synthesis of 2,4-Diphenyl-1H-imidazole *(**3aa**)*

A mixture of benzamidoxime (1a, 136.1 mg, 1.0 mmol), phenylacetylene (2a, 204.1 mg, 2.0 mmol) and Cs_2_CO_3_ (815.1 mg, 2.5 mmol) in DMSO (4.0 mL), in a 25 mL screw-capped thick-walled Pyrex tube was stirred at 100 °C for 24 h in an oil bath. After the reaction mixture was cooled to room temperature, it was poured into a solvent mixture of water (50.0 mL) and ethyl acetate (20.0 mL), and the two phases were then separated. The aqueous layer was extracted with ethyl acetate (3 × 20.0 mL). The combined organic extracts were dried over anhydrous Na_2_SO_4_. After removal of the solvent under reduced pressure, the residue was purified by column chromatography on silica gel with petroleum ether/ethyl acetate (gradient mixture ratio from 100:0 to 70:20) as eluent, to afford 3aa as a white solid (160.4 mg, 73%).

The characterization data for known products of **3aa**, **3ab**, **3af**, **3ah**-**3al**, **3an**, **3ao**, **3aq**, **3ar**, **3ba**, **3ca** and **3da** reported in the Supplementary Materials. Each of **3ac**, **3ad**, **3ae**, **3ag**, **3am**, **3ap** and **3ed** are new compounds, and their spectroscopic data are reported below.

### 3.3. Characterization Data of Products

*4-(4-Ethylphenyl)-2-phenyl-1H-imidazole* (**3ac**):

White solid (190.4 mg, 77%). ^1^H-NMR (400 MHz, DMSO-*d*_6_) *δ* 12.71 (s, 1H), 8.17 (d, *J* = 7.6 Hz, 2H), 7.87 (d, *J* = 7.6 Hz, 2H), 7.70 (s, 1H), 7.50 (t, *J* = 7.6 Hz, 2H), 7.37 (t, *J* = 7.3 Hz, 1H), 7.25 (d, *J* = 8.0 Hz, 2H), 2.61 (q, *J* = 7.5 Hz, 2H), 1.20 (t, *J* = 7.5 Hz, 3H); ^13^C-NMR (100 MHz, DMSO-*d*_6_) *δ* 146.1, 141.9, 130.8, 128.7, 128.0, 127.9, 125.0, 124.5, 28.0, 15.6 HRMS (ESI) *m/z*: [M + H]^+^ calcd for C_17_H_16_N_2_, 249.1386; found 249.1388.

*2-Phenyl-4-(4-n-propylphenyl)-1H-imidazole* (**3ad**):

White solid (206.7 mg, 79%). ^1^H-NMR (400 MHz, DMSO-*d*_6_) *δ* 12.64 (sbr, 1H), 8.15 (d, *J* = 7.7 Hz, 2H), 7.85 (d, *J* = 7.7 Hz, 2H), 7.66 (s, 1H), 7.49 (t, *J* = 7.5 Hz, 2H), 7.38 (t, *J* = 7.3 Hz, 1H), 7.22 (d, *J* = 7.8 Hz, 2H), 2.56 (t, *J* = 7.4 Hz, 2H), 1.69–1.53 (m, 2H), 0.91 (t, *J* = 7.4 Hz, 3H); ^13^C-NMR (100 MHz, DMSO-*d*_6_) δ 146.0, 140.2, 130.8, 129.3, 128.5, 128.4, 127.9, 125.0, 124.4, 118.7, 115.3, 37.0, 24.0, 13.5; HRMS (ESI) *m/z*: [M + H]^+^ calcd for C_18_H_18_N_2_, 263.1543; found 263.1540.

*4-(4-Butylphenyl)-2-phenyl-1H-imidazole* (**3ae**):

White solid (201.3 mg, 73%). ^1^H NMR (400 MHz, DMSO-*d*_6_) *δ* 12.64 (sbr, 1H), 8.14 (d, *J* = 7.4 Hz, 2H), 7.83 (d, *J* = 7.3 Hz, 2H), 7.65 (s, 1H), 7.48 (t, *J* = 7.2 Hz, 2H), 7.36 (t, *J* = 7.0 Hz, 1H), 7.21 (d, *J* = 7.5 Hz, 2H), 2.58 (t, *J* = 7.3 Hz, 2H), 1.63–1.50 (m, 2H), 1.40–1.22 (m, 2H), 0.90 (t, *J* = 7.1 Hz, 3H); ^13^C-NMR (100 MHz, DMSO-*d*_6_) *δ* 146.0, 140.3, 130.8, 129.3, 128.5, 128.3, 127.8, 124.9, 124.4, 34.6, 33.1, 21.7, 13.7; HRMS (ESI) *m/z*: [M + H]^+^ calcd for C_19_H_20_N_2_, 277.1699; found 277.1698.

*4-(4-(4-n-Pentylcyclohexyl)phenyl)-2-phenyl-1H-imidazole* (**3ag**):

White Solid (256.7 mg, 69%). ^1^H-NMR (400 MHz, DMSO-*d*_6_) *δ* 12.58 (s, 1H), 8.00 (d, *J* = 7.6 Hz, 2H), 7.74 (d, *J* = 7.8 Hz, 2H), 7.63 (s, 1H), 7.46 (t, *J* = 7.6 Hz, 2H), 7.35 (t, *J* = 7.3 Hz, 1H), 7.22 (d, *J* = 8.0 Hz, 2H), 2.50–2.38 (m, 1H), 1.83–1.80 (m, 4H), 1.52–1.37 (m, 2H), 1.35–1.13 (m, 10H), 1.10–0.95 (m, 2H), 0.88 (t, *J* = 6.6 Hz, 3H); ^13^C-NMR (100 MHz, DMSO-*d*_6_) *δ* 145.8, 145.4, 130.6, 128.6, 128.0, 126.7, 124.8, 124.3, 43.9, 36.8, 36.6, 33.8, 33.1, 31.6, 26.0, 22.1, 13.9; HRMS (ESI) *m/z*: [M + H]^+^ calcd for C_26_H_32_N_2_, 373.2638; found 373.2635.

*4-(Naphthalen-2-yl)-2-phenyl-1H-imidazole* (**3am**):

White solid (148.3 mg, 55%). ^1^H-NMR (400 MHz, DMSO-*d*_6_) *δ* 12.72 (s, 1H), 8.39 (s, 1H), 8.09–7.85 (m, 7H), 7.55–7.34 (m, 5H); ^13^C-NMR (100 MHz, DMSO-*d*_6_) *δ* 146.0, 140.9, 133.3, 132.1, 131.8, 130.5, 128.6, 128.1, 127.7, 127.6, 127.4, 126.1, 125.1, 125.0, 124.9, 123.7, 121.7, 114.9; HRMS (ESI) *m*/*z*: [M + H]^+^ calcd for C_19_H_14_N_2_, 271.1230; found 271.1235.

*2-Phenyl-4,5-di-m-tolyl-1H-imidazole* (**3ap**):

White solid (178.1 mg, 55%). ^1^H-NMR (400 MHz, DMSO-*d*_6_) *δ* 12.59 (s, 1H), 8.08 (d, *J* = 8.3 Hz, 3H), 7.50–7.43 (m, 3H), 7.40–7.24 (m, 5H), 7.22–7.12 (m, 2H), 7.04 (d, *J* = 6.9 Hz, 1H), 2.34 (s, 3H), 2.27 (s, 3H); ^13^C-NMR (100 MHz, DMSO-*d*_6_) *δ* 145.2, 137.6, 137.0, 135.0, 130.9, 130.3, 129.5, 128.8, 128.5, 128.3, 128.2, 128.1, 128.0, 127.8, 127.6, 127.0, 125.5, 125.1, 124.1, 21.0, 20.9; HRMS (ESI) *m*/*z*: [M + H]^+^ calcd for C_23_H_20_N_2_, 325.1699; found 325.1697.

*2-Methyl-4-(4-n-propylphenyl)-1H-imidazole* (**3ed**):

Waxy oil (98.1 mg, 49%). ^1^H-NMR (400 MHz, DMSO-*d*_6_) *δ* 7.55 (d, *J* = 8.1 Hz, 2H), 7.28 (s, 1H), 7.09 (d, *J* = 8.2 Hz, 2H), 2.50–2.41 (m, 2H), 2.26 (s, 3H), 1.60–1.46 (m, 2H), 0.85 (t, *J* = 7.3 Hz, 3H); ^13^C-NMR (100 MHz, DMSO-*d*_6_) *δ* 144.1, 1, 139.5, 131.5, 128.4, 123.9, 36.9, 24.0, 13.9, 13.6; HRMS (ESI) *m*/*z*: [M + H]^+^ calcd for C_13_H_16_N_2_, 201.0681; found 201.0687.

## 4. Conclusions

In summary, we developed a simple and efficient method to prepare 2,4-disubstituted imidazoles in moderate to good yields from easily available starting materials of amidoximes and terminal alkynes promoted by Cs_2_CO_3_ in DMSO. The significant advantages of the present procedure include the formation of imidazoles under transition-metal-free and ligand-free conditions, high atom-utilization, and a broad substrate scope. 2-substituted and 2,4,5-trisubstituted imidazoles could be also prepared under the similar conditions by using 1-(trimethylsilyl)acetylene and internal alkynes.

## Figures and Tables

**Table 1:**
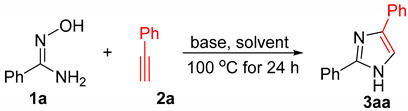
Optimizing reaction conditions for imidazole synthesis *^a^*.

Entry	2a (equiv)	Base(equiv)	Solvent	Yield(%)*b*
1	2.0	Na2CO3(4)	DMSO	10
2	2.0	K2CO3(4)	DMSO	34
3	2.0	KOH(4)	DMSO	53
4	2.0	KO^*t*^Bu(4)	DMSO	41
5	2.0	Cs2CO3(4)	DMSO	75
6	2.0	Cs2CO3(4)	THF	10
7	2.0	Cs2CO3(4)	Dioxane	14
8	2.0	Cs2CO3(4)	DMF	21
9	1.0	Cs2CO3(4)	DMSO	59
10	1.5	Cs2CO3(4)	DMSO	68
**11** 12 13 14	**2.0** 2.0 2.0 2.0	**Cs2CO3(2.5)** Cs2CO3(1.0) Cs2CO3(0.5) --	**DMSO** DMSO DMSO DMSO	**73** 33 19 0

*^a^* The reactions were carried out using **1a** (1.0 mmol), **2a** (1.0~2.0 equiv), and base in 4.0 mL of solvent in a sealed tube at 100 ^o^C for 24 h. *^b^* yields of **3aa** are isolated yields.

## Supplementary Materials

The following are available online. The characterization data of the known products, copies of ^1^H- and ^13^C-NMR charts of all products, X-ray structural details (including CIF files) of **3aa**, and computational predicted energies and cartesian coordinates.
