# Supplementary material for: Base-Promoted Annulation of Amidoximes with Alkynes: Simple Access to 2,4-Disubstituted Imidazoles
Source: Molecules. 2020 Aug 9;25(16):3621. doi: 10.3390/molecules25163621 (PMC7463794; doi:10.3390/molecules25163621)
Supplement: Supplementary file 1 [file molecules-25-03621-s001.pdf]

Supplementary File

Base-Promoted Annulation of Amidoximes with Alkynes: Simple Access to 2,4-Disubstituted Imidazoles

Hina Mehmood, Muhammad Asif Iqbal, Le Lu, and Ruimao Hua*

Department of Chemistry, Tsinghua University, Key Laboratory of Organic Optoelectronics & Molecular Engineering of Ministry of Education, Beijing 100084, China
E-mail: ruimao@mail.tsinghua.edu.cn

Contents Page

1. Characterization data for known products S2
2. Copies of ^{1}H NMR and ^{13}C NMR spectra of all the products S4
3. X-ray structural details of **3aa** S26
4. Computational predicted energies and cartesian coordinates S34

1. Characterization data for known products.

2,4-Diphenyl-1*H*-imidazole (**3aa**) [1]: White solid (160.4 mg, 73%); ^{1}H NMR (400 MHz, DMSO-d_6) δ 12.64 (s_{br}, 1H), 8.04 (d, *J* = 7.5 Hz, 2H), 7.87 (d, *J* = 7.5 Hz, 2H), 7.72 (s_{br}, 1H), 7.48 (t, *J* = 7.6 Hz, 2H), 7.43 – 7.32 (m, 3H), 7.22 (t, *J* = 7.3 Hz, 1H); ^{13}C NMR (100 MHz, DMSO-d_6) δ 146.6, 131.1, 129.2, 129.0, 128.6, 126.8, 125.5, 124.9; GC-MS m/z: 220 (M^+).

2-Phenyl-4-(*p*-tolyl)-1*H*-imidazole (**3ab**) [2]: White Solid (175.3 mg, 75%); ^{1}H NMR (400 MHz, DMSO-d_6) δ 12.66 (s, 1H), 8.10 (d, *J* = 7.6 Hz, 2H), 7.80 (d, *J* = 7.5 Hz, 2H), 7.69 (s_{br}, 1H), 7.48 (t, *J* = 7.7 Hz, 2H), 7.36 (t, *J* = 7.3 Hz, 1H), 7.21 (d, *J* = 7.8 Hz, 2H), 2.31 (s, 3H); ^{13}C NMR (100 MHz, DMSO-d_6) δ 145.9, 135.4, 130.7, 129.1, 128.7, 128.0, 125.0, 124.4, 20.8; GC-MS m/z: 234 (M^+).

4-(4-(*t*-butyl)Phenyl)-2-phenyl-1*H*-imidazole (**3af**) [3]: White Solid (231.7 mg, 84%); ^{1}H NMR (400 MHz, DMSO-d_6) δ 12.66 (s, 1H), 8.10 (d, *J* = 7.4 Hz, 2H), 7.82 (d, *J* = 7.6 Hz, 2H), 7.68 (s_{br}, 1H), 7.52 – 7.32 (m, 5H), 1.30 (s, 9H); ^{13}C NMR (100 MHz, DMSO-d_6) δ 148.6, 145.9, 130.7, 128.6, 128.0, 125.2, 125.0, 124.2, 34.1, 31.1; GC-MS m/z: 276 (M^+).

4-([1,1'-Biphenyl]-4-yl)-2-phenyl-1*H*-imidazole (**3ah**) [4]: White solid (239.6 mg, 81%); ^{1}H NMR (400 MHz, DMSO-d_6) δ 12.80 (s, 1H), 8.19 (d, *J* = 7.6 Hz, 2H), 8.06 (d, *J* = 7.8 Hz, 2H), 7.84 (s, 1H), 7.76 – 7.67 (m, 4H), 7.55 – 7.30 (m, 6); ^{13}C NMR (100 MHz, DMSO-d_6) δ 145.3, 139.9, 137.9, 130.7, 128.8, 128.7, 128.1, 127.1, 126.7, 126.3, 125.1, 125.0; GC-MS m/z: 296 (M^+).

4-(4-Chlorophenyl)-2-phenyl-1*H*-imidazole (**3ai**) [5]: White solid (172.5 mg, 68%); ^{1}H NMR (400 MHz, DMSO-d_6) δ 12.76 (s, 1H), 8.05 (d, *J* = 7.0 Hz, 2H), 7.91 – 7.80 (m, 3H), 7.57 (d, *J* = 7.4 Hz, 2H), 7.47 (t, *J* = 7.2 Hz, 2H), 7.36 (t, *J* = 7.2 Hz, 1H); ^{13}C NMR (100 MHz, DMSO-d_6) δ 146.1, 139.9, 133.9, 131.3, 130.4, 128.7, 128.2, 126.3, 125.0, 118.9, 114.9; GC-MS m/z: 254 (M^+).

4-(4-Bromophenyl)-2-phenyl-1*H*-imidazole (**3aj**) [1]: White solid (175.6 mg, 59%); ^{1}H NMR (400 MHz, DMSO-d_6) δ 12.79 (s_{br}, 1H), 8.09 (d, *J* = 7.4 Hz, 2H), 8.00 – 7.74 (m, 3H), 7.39 – 7.32 (m, 4H), 7.36 (t, *J* = 7.4 Hz, 1H); ^{13}C NMR (100 MHz, DMSO-d_6) δ 146.2, 140.0, 133.6, 130.5, 128.7, 128.5, 128.2, 126.0, 125.0, 114.8; GC-MS m/z: 298 (M^+).

2-Phenyl-4-(*m*-tolyl)-1*H*-imidazole (**3ak**) [3]: White solid (147.2 mg, 63%); ^{1}H NMR (400 MHz, DMSO-d_6) δ 12.76 (s, 1H), 8.13 (d, *J* = 7.6 Hz, 2H), 7.90 (s_{br}, 1H), 7.53 – 7.42 (m, 3H), 7.37 (t, *J* = 7.3 Hz, 1H), 7.32 – 7.24 (m, 2H), 7.19 (t, *J* = 7.2 Hz, 1H), 2.54 (s, 3H); ^{13}C NMR (100 MHz, DMSO-d_6) δ 145.4, 134.5, 130.8, 130.7, 128.7, 128.1(2C), 126.5, 125.7, 125.1, 21.6; GC-MS m/z: 234 (M^+).

4-(3-Chlorophenyl)-2-phenyl-1*H*-imidazole (**3al**) [1]: White solid (137.1 mg, 54%); ^{1}H NMR (400 MHz, DMSO-d_6) δ 12.81 (s, 1H), 8.08 (d, *J* = 7.5 Hz, 2H), 7.99 (s, 1H), 7.91 – 7.83 (m, 2H), 7.48 (t, *J* = 7.6 Hz, 2H), 7.43 – 7.33 (m, 2H), 7.25 (d, *J* = 8.0 Hz, 1H); ^{13}C NMR (100 MHz, DMSO-d_6) δ 146.4, 133.5, 130.4, 130.3, 128.7, 128.3, 125.9, 125.1, 123.9, 122.8; GC-MS m/z: 254 (M^+).

2-Phenyl-4-(thiophen-2-yl)-1*H*-imidazole (**3an**) [5]: White waxy liquid (126.3 mg, 56%); ^{1}H NMR (400 MHz, DMSO-d_6) δ 12.70 (s, 1H), 7.98 (d, *J* = 7.6 Hz, 2H), 7.59 (s_{br}, 1H), 7.43 (t, *J* =

7.7 Hz, 2H), 7.36 – 7.27 (m, 3H), 7.02 (t, *J* = 4.8 Hz 1H); ^{13}C NMR (100 MHz, DMSO-d_6) δ 145.8, 138.6, 136.6, 130.3, 128.7, 128.2, 127.6, 125.0, 123.3,121.5, 113.5; GC-MS m/z: 226 (M^+).

2,4,5-Triphenyl-1*H*-imidazole (**3ao**) [2]: White solid (180.7 mg, 61%); ^{1}H NMR (400 MHz, DMSO-d_6) δ 8.15 (d, *J* = 7.4 Hz, 2H), 7.58 – 7.48 (m, 6H), 7.48 – 7.31 (m, 7H); ^{13}C NMR (100 MHz, DMSO-d_6) δ 145.0, 131.6, 129.0, 128.7, 128.6, 128.4, 127.9, 127.6, 125.7; GC-MS m/z: 296 (M^+).

2-Phenyl-1*H*-imidazole (**3aq**) [6]: White solid (96.2 mg, 67%); ^{1}H NMR (400 MHz, DMSO-d_6) δ 12.42 (s_{br}, 1H), 7.93 (d, *J* = 7.5 Hz, 2H), 7.43 (t, *J* = 7.6 Hz, 2H), 7.32 (t, *J* = 7.3 Hz, 1H), 7.12 (s, 2H); ^{13}C NMR (100 MHz, DMSO-d_6) δ 145.4, 130.8, 128.9, 127.8, 124.7; GC-MS m/z: 144 (M^+).

4-Methyl-2-phenyl-1*H*-imidazole (**3ar**) [7]: White solid (120.4 mg, 76%); ^{1}H NMR (400 MHz, DMSO-d_6) δ 11.78 (s_{br}, 1H), 7.88 (d, *J* = 7.4 Hz, 2H), 7.40 (t, *J* = 7.6 Hz, 2H), 7.29 (t, *J* = 7.4 Hz, 1H), 6.80 (s, 1H), 2.19 (s, 3H); ^{13}C NMR (100 MHz, DMSO-d_6) δ 144.6, 130.9, 128.5, 127.4, 124.4, 11.7; GC-MS m/z: 158 (M^+).

4-Phenyl-2-(*p*-tolyl)-1*H*-imidazole (**3ba**) [3]: White solid (168.2 mg, 72%); ^{1}H NMR (400 MHz, DMSO-d_6) δ 12.58 (s_{br}, 1H), 7.95 (d, *J* = 8.0 Hz, 2H), 7.87 (d, *J* = 7.2 Hz, 2H), 7.68 (s_{br}, 1H), 7.36 (t, *J* = 7.7 Hz, 2H), 7.24 (d, *J* = 8.0 Hz, 2H), 7.18 (t, *J* = 7.4 Hz, 1H), 2.29 (s, 3H); ^{13}C NMR (100 MHz DMSO-d_6) δ 146.3, 137.5, 129.3, 128.5, 128.0, 126.2, 125.0, 124.4, 20.8; GC-MS m/z: 234 (M^+).

4-Phenyl-2-(4-(trifluoromethyl)phenyl)-1*H*-imidazole (**3ca**) [8]: White solid (187.4 mg, 65%); ^{1}H NMR (400 MHz, DMSO-d_6) δ 12.99 (s, 1H), 8.30 (d, *J* = 6.6 Hz, 2H), 8.02 – 7.76 (m, 5H), 7.41 (t, *J* = 7.3 Hz, 2H), 7.29 – 7.18 (m, 1H); ^{13}C NMR (100 MHz, DMSO-d_6) δ 144.5, 141.8, 136.5, 134.3, 128.6, 128.4, 128.38, 128.33, 128.01, 127.6, 126.8, 126.4, 125.8, 125.6, 125.4, 124.8, 124.6, 122.9, 121.3, 120.2, 115.1; GC-MS m/z: 288 (M^+).

4-Phenyl-2-(thiophen-2-yl)-1*H*-imidazole (**3da**) [9]: White solid (187.2 mg, 83%); ^{1}H NMR (400 MHz, DMSO-d_6) δ 12.79 (s, 1H), 7.99 – 7.60 (m, 4H), 7.52 (d, *J* = 4.6 Hz, 1H), 7.40 (t, *J* = 7.6 Hz, 2H), 7.23 (t, *J* = 7.2 Hz, 1H), 7.21 – 7.09 (m, 1H); ^{13}C NMR (100 MHz, DMSO-d_6) δ 142.1, 141.0, 134.4, 128.5, 127.8, 126.4, 125.9, 124.4, 123.9, 113.1; GC-MS m/z: 226 (M^+).

2. Copies of ^{1}H NMR and ^{13}C NMR spectra of all the products.

^{1}H NMR spectrum of compound **3aa**

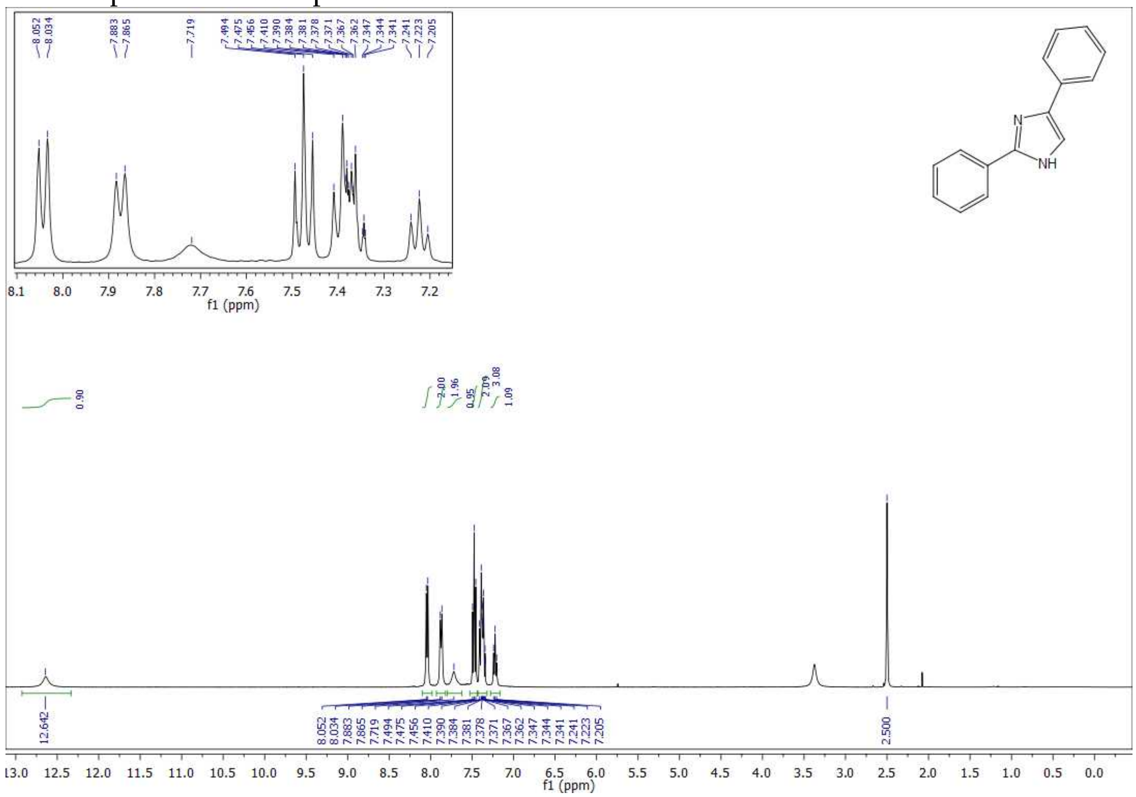

^{13}C NMR spectrum of compound **3aa**

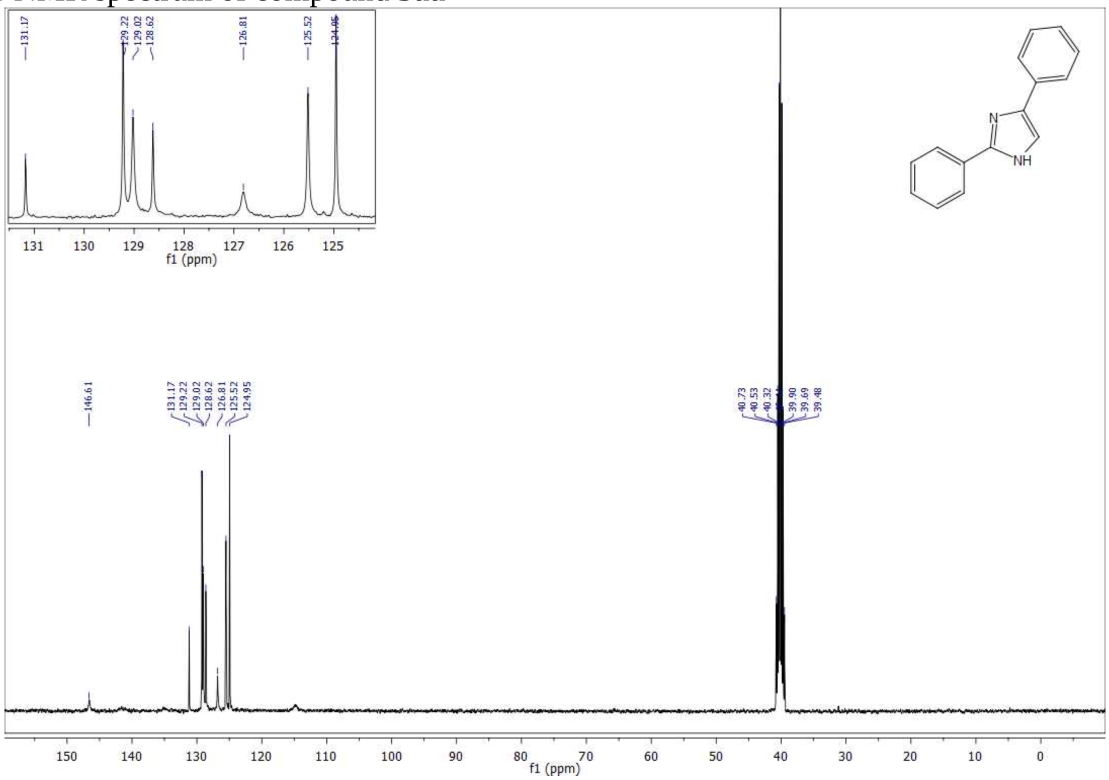

^{1}H NMR spectrum of compound **3ab**

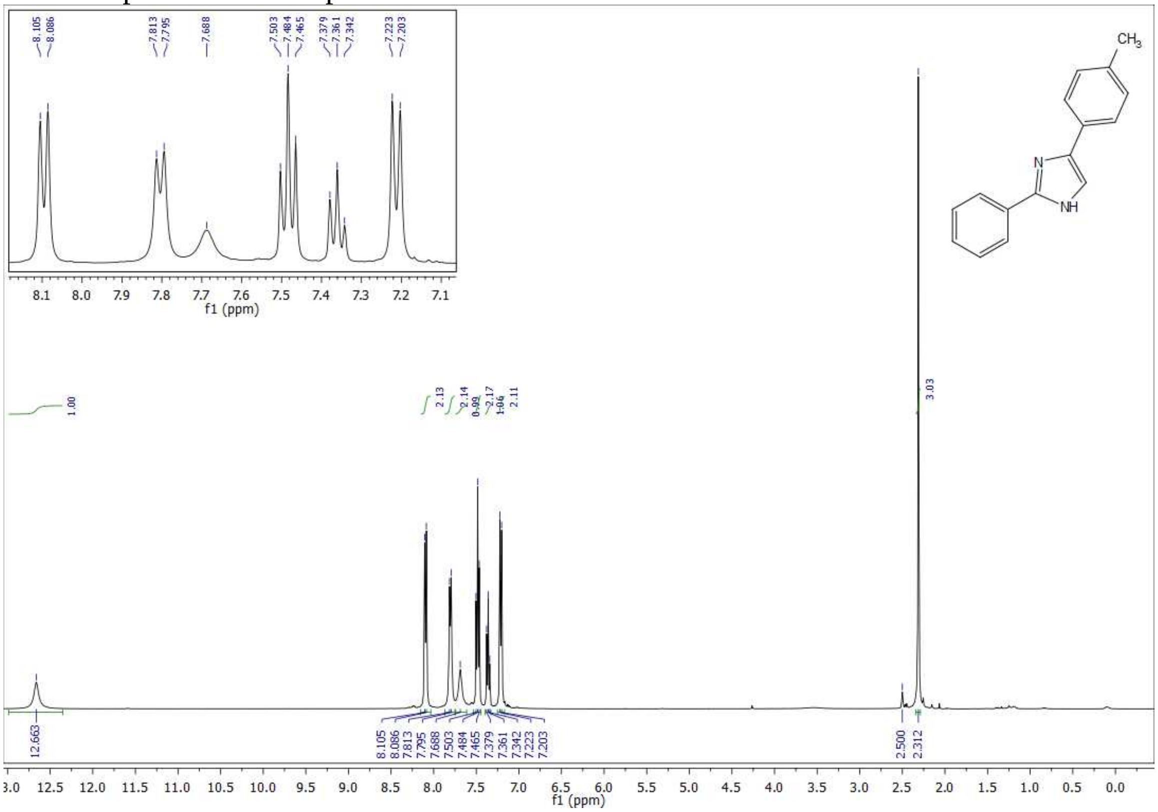

^{13}C NMR spectrum of compound **3ab**

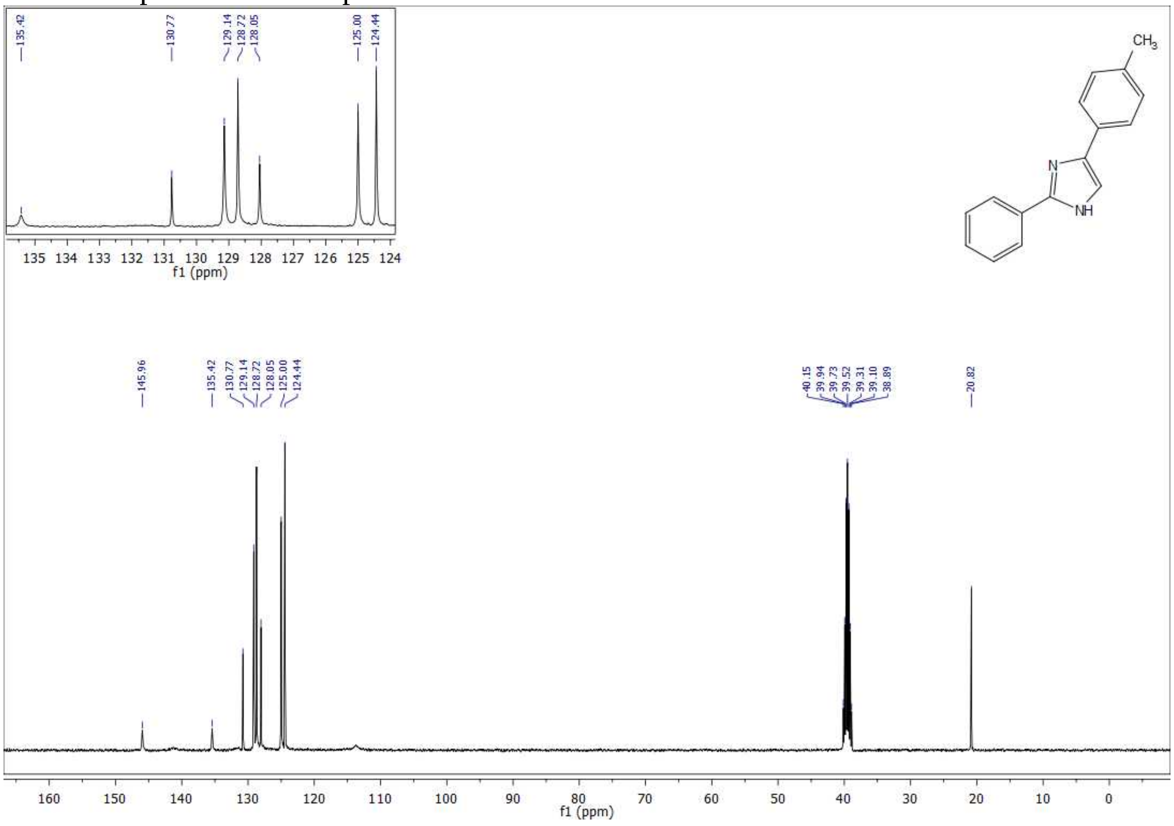

^{1}H NMR spectrum of compound **3ac**

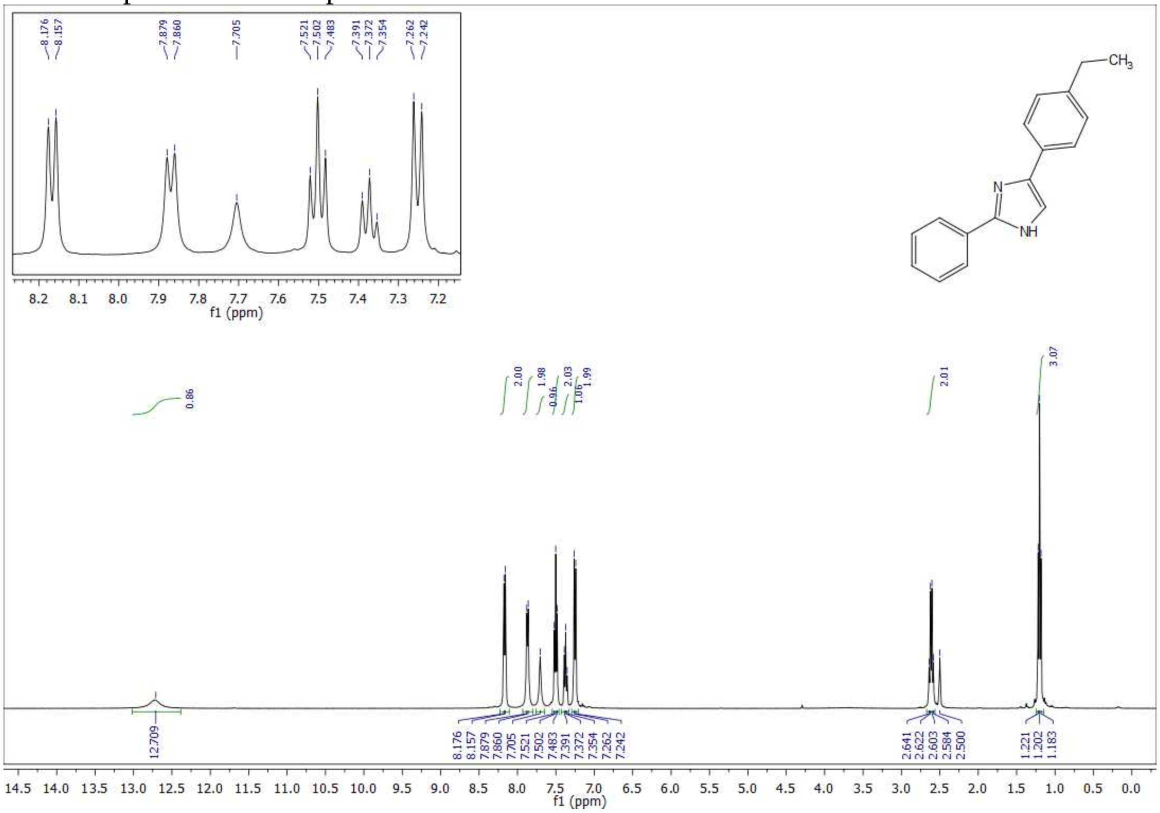

^{13}C NMR spectrum of compound **3ac**

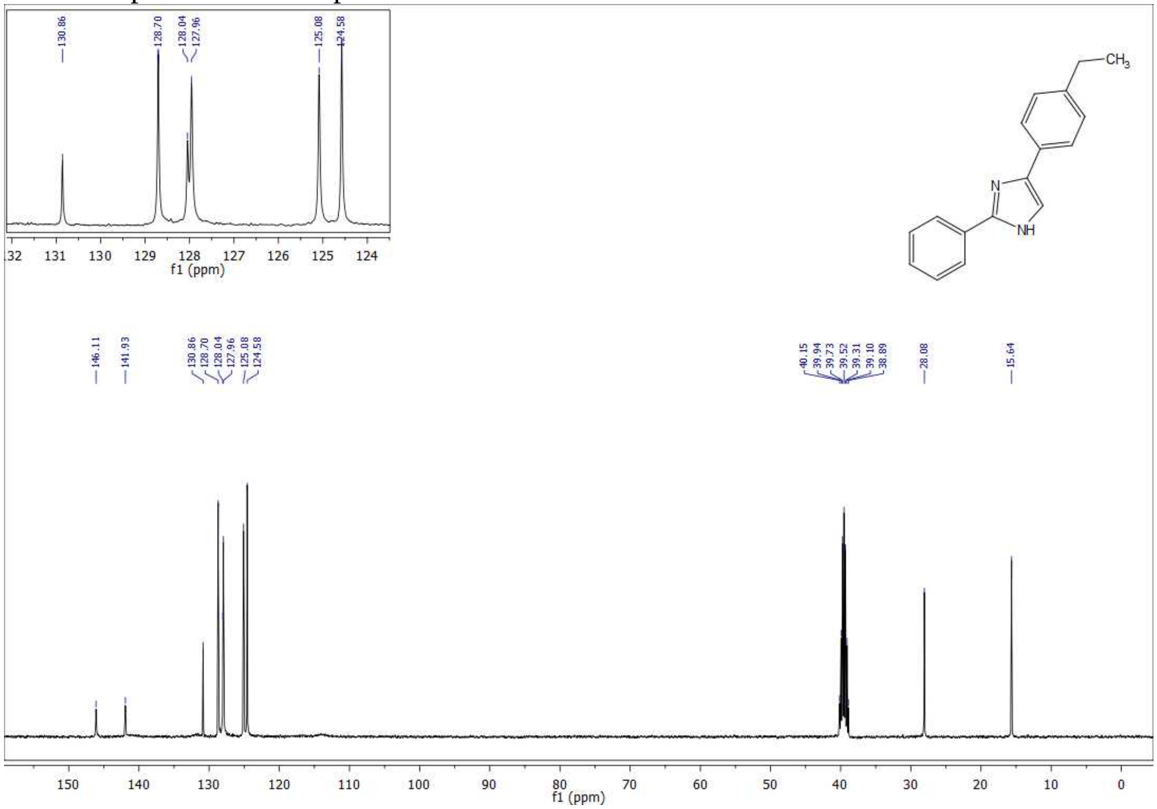

^{1}H NMR spectrum of compound **3ad**

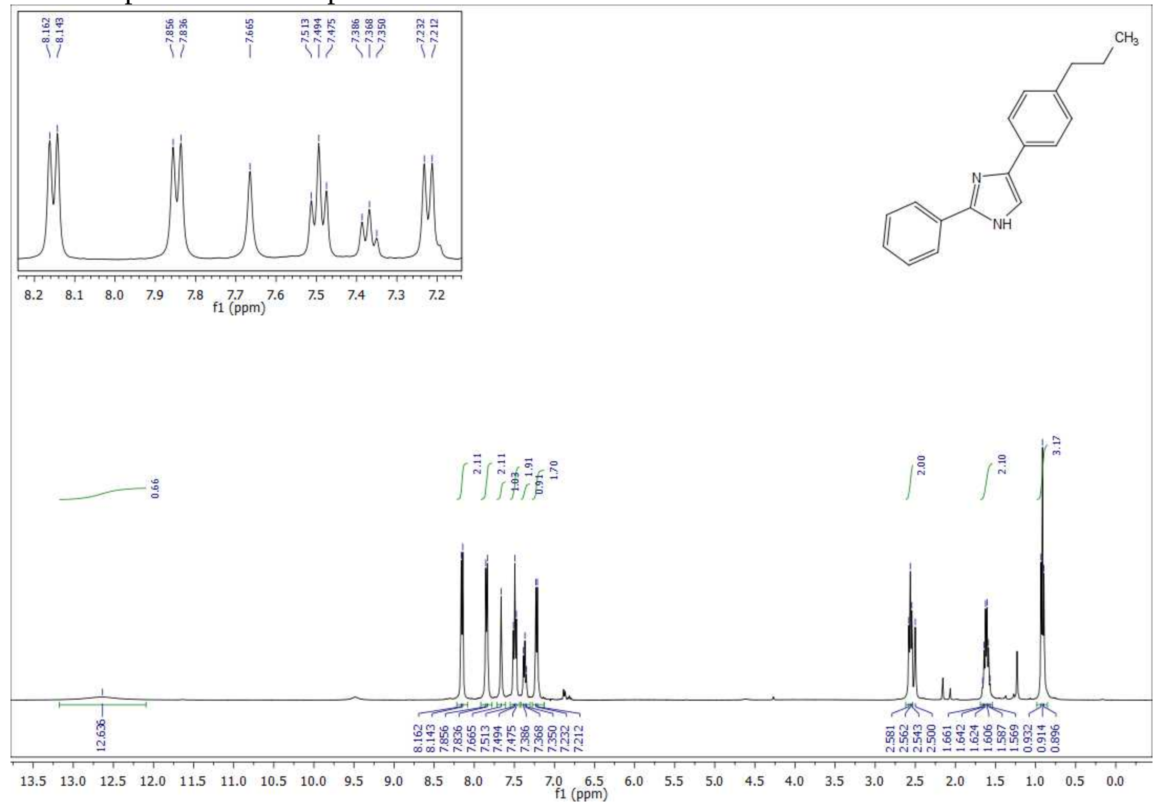

^{13}C NMR spectrum of compound **3ad**

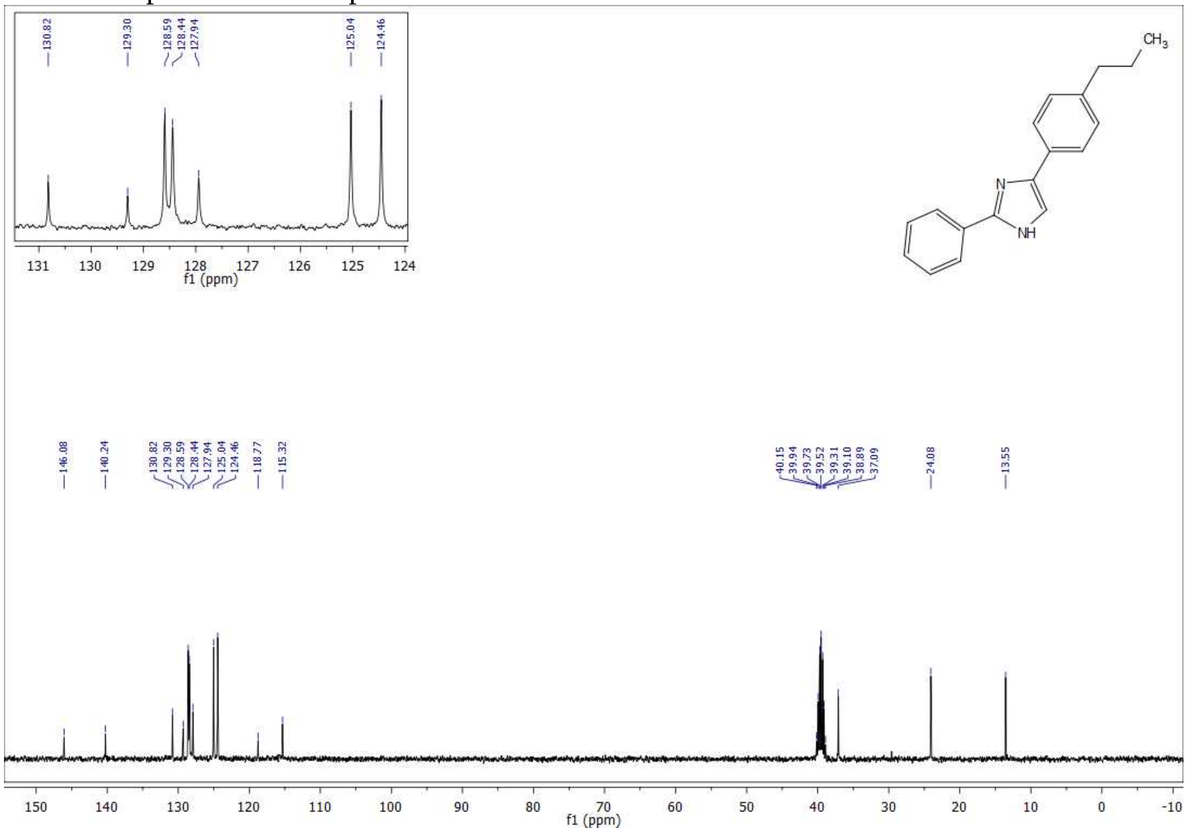

^{1}H NMR spectrum of compound **3ae**

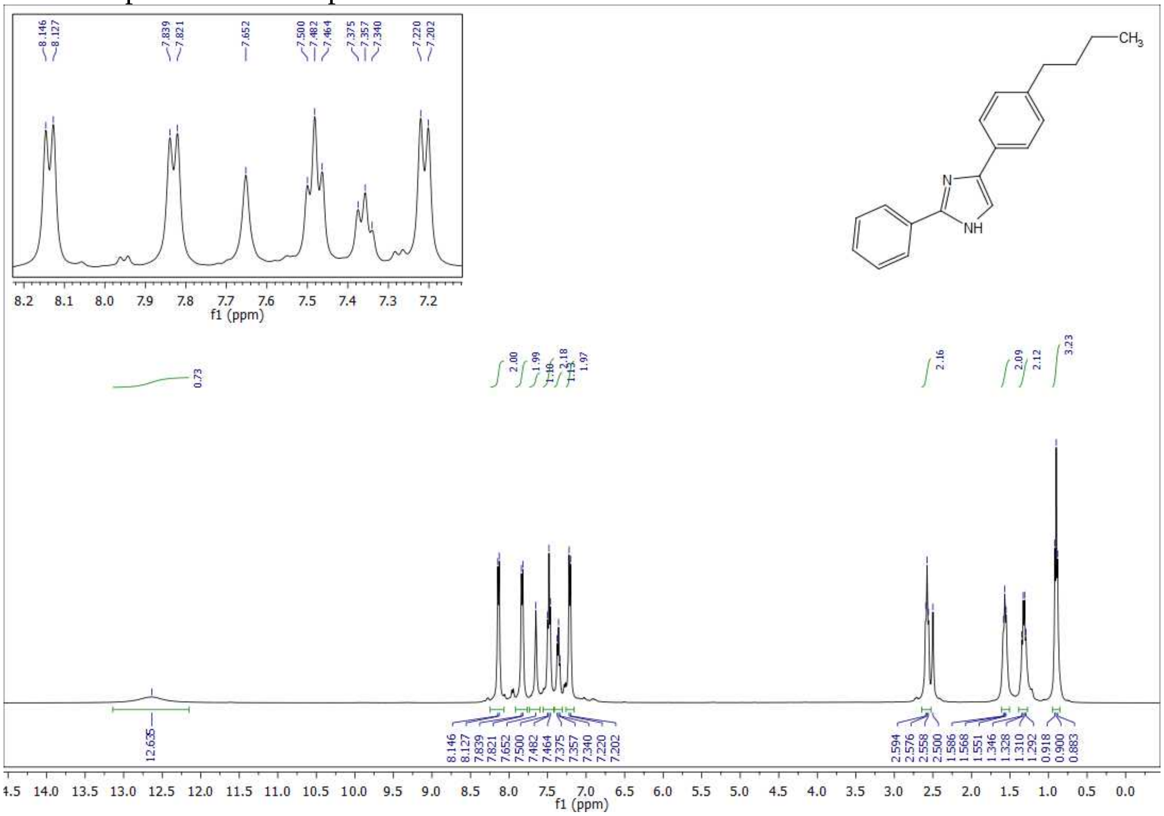

^{13}C NMR spectrum of compound **3ae**

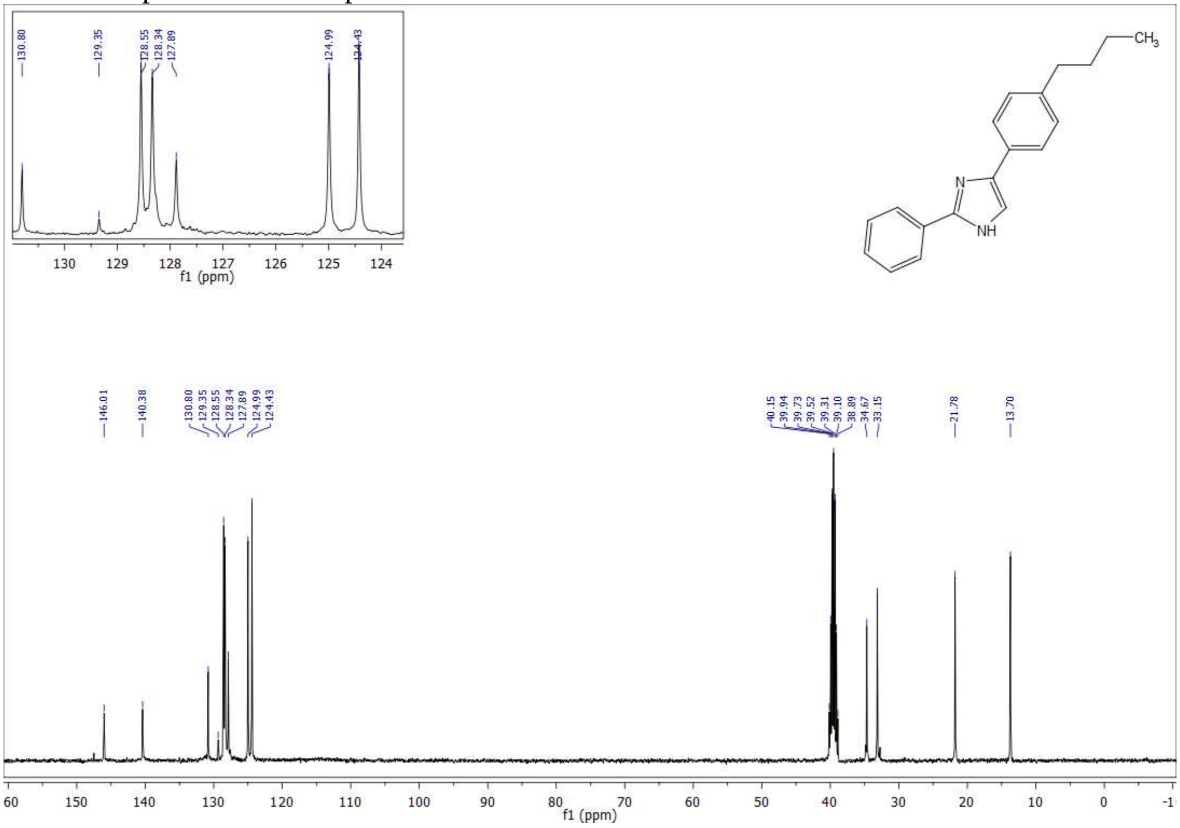

^{1}H NMR spectrum of compound **3af**

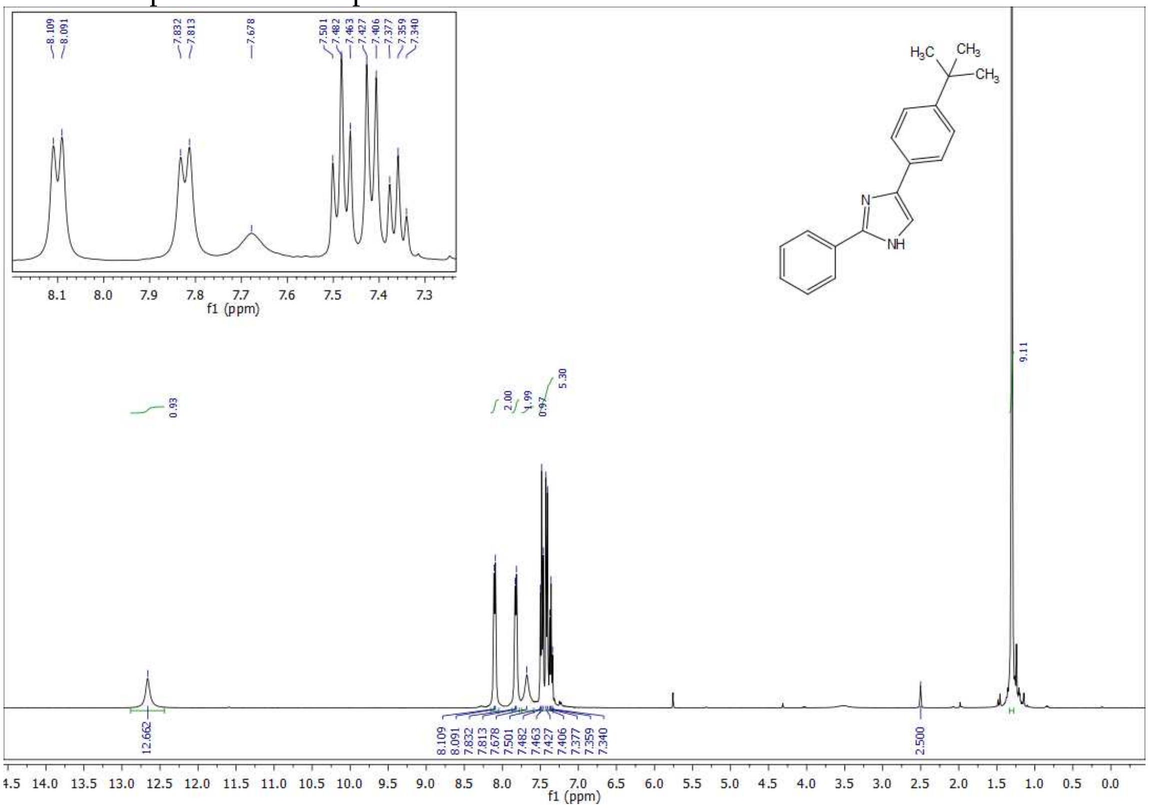

^{13}C NMR spectrum of compound **3af**

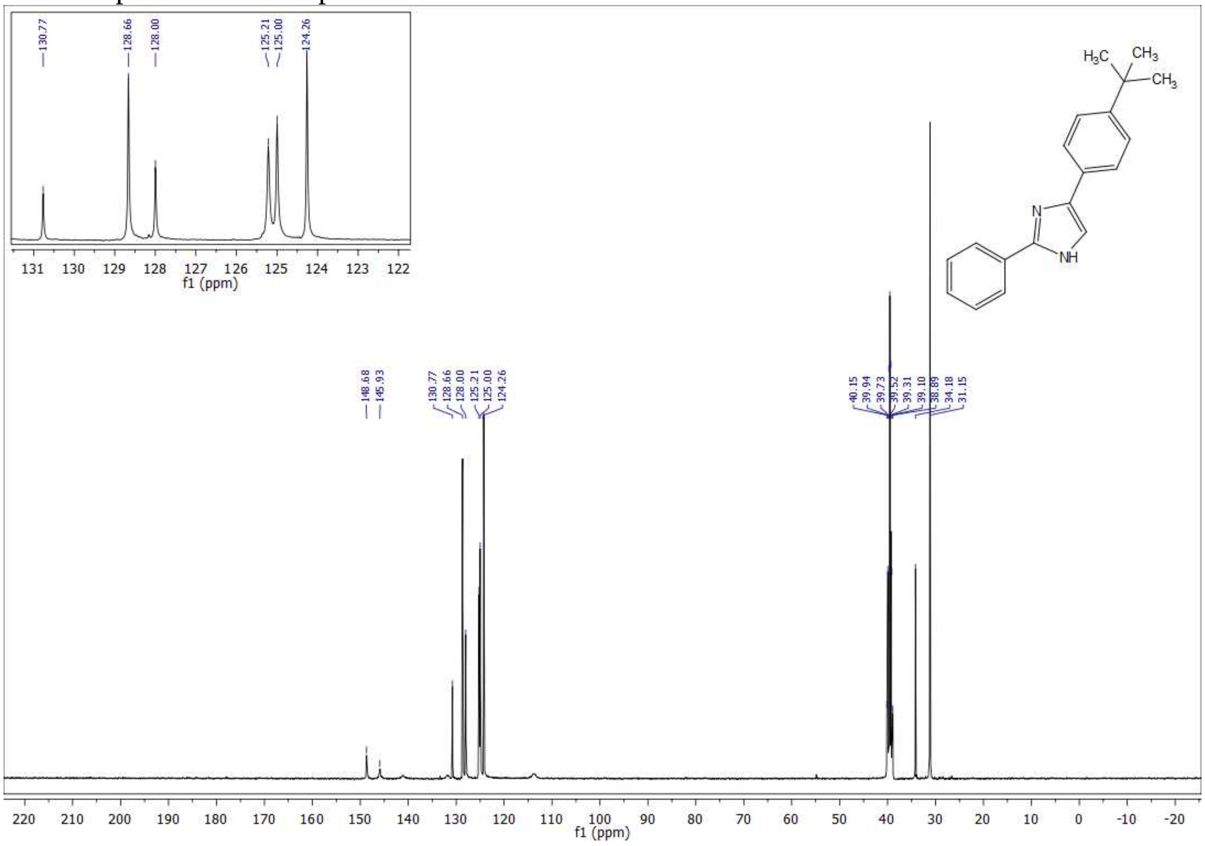

^{1}H NMR spectrum of compound **3ag**

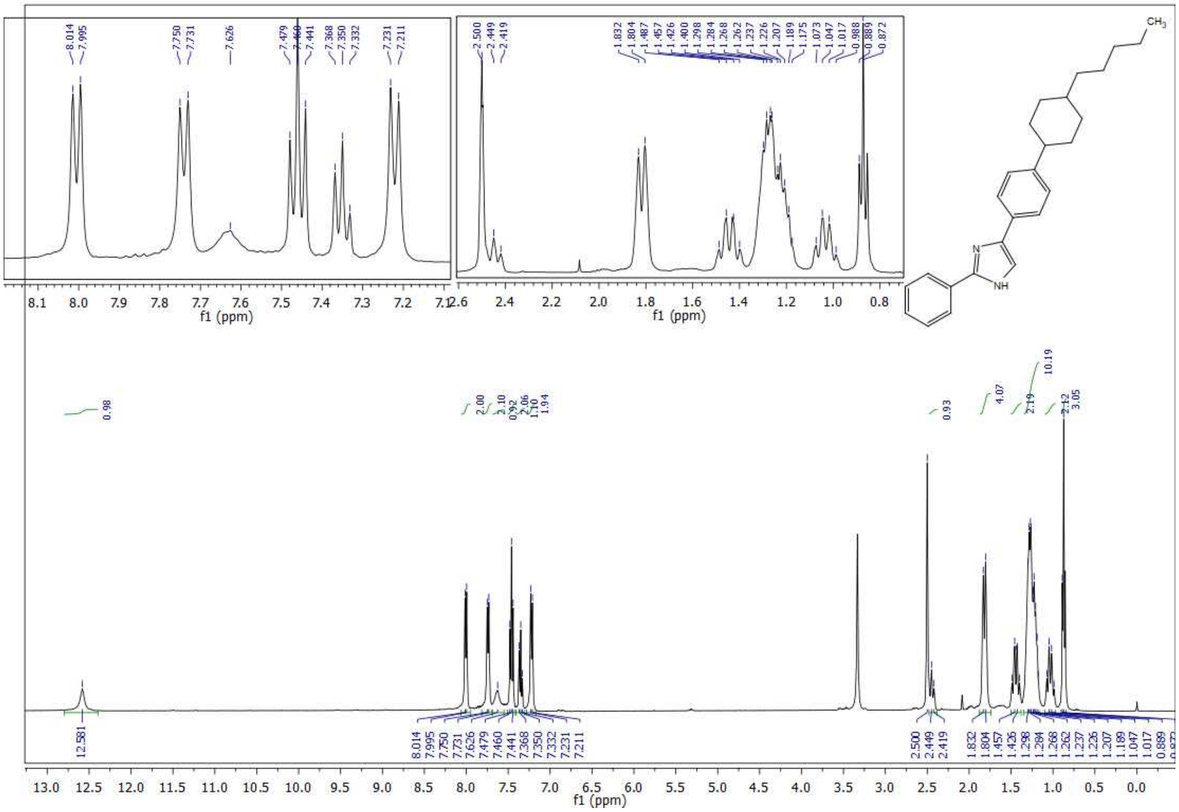

^{13}C NMR spectrum of compound **3ag**

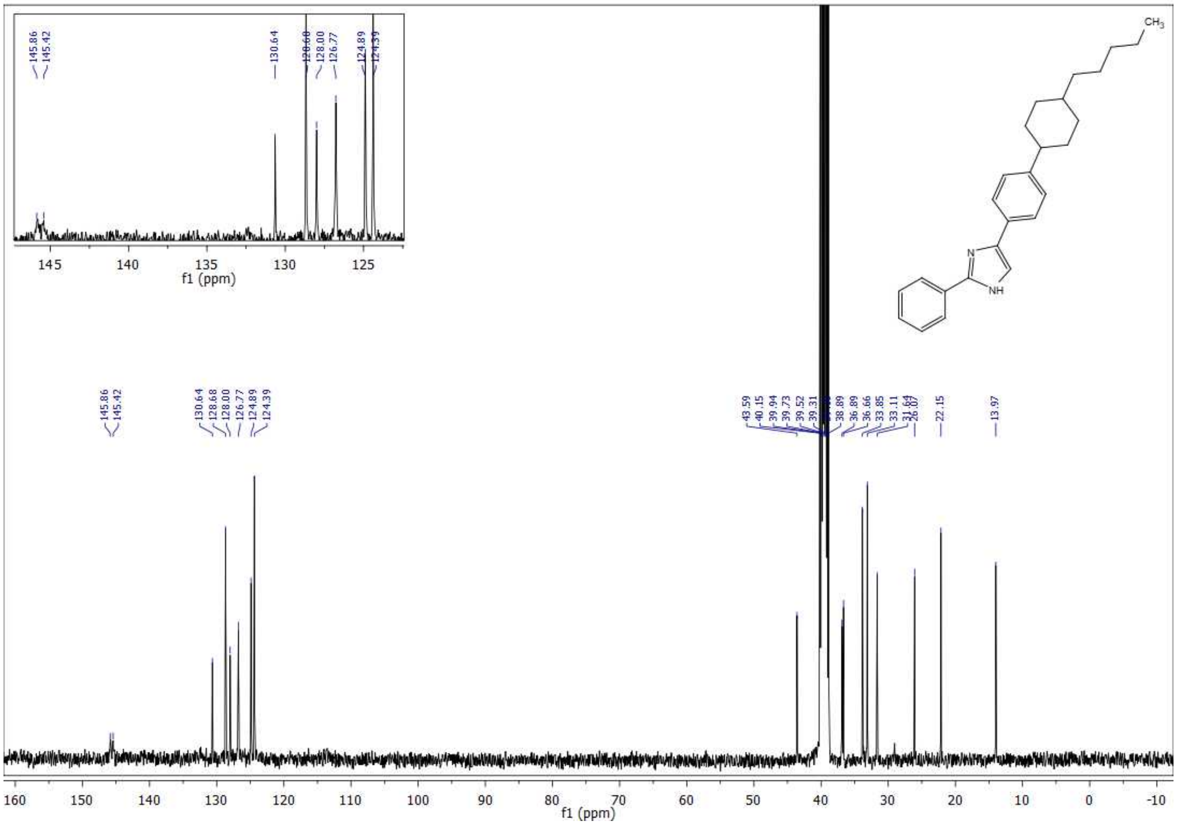

^{1}H NMR spectrum of compound **3ah**

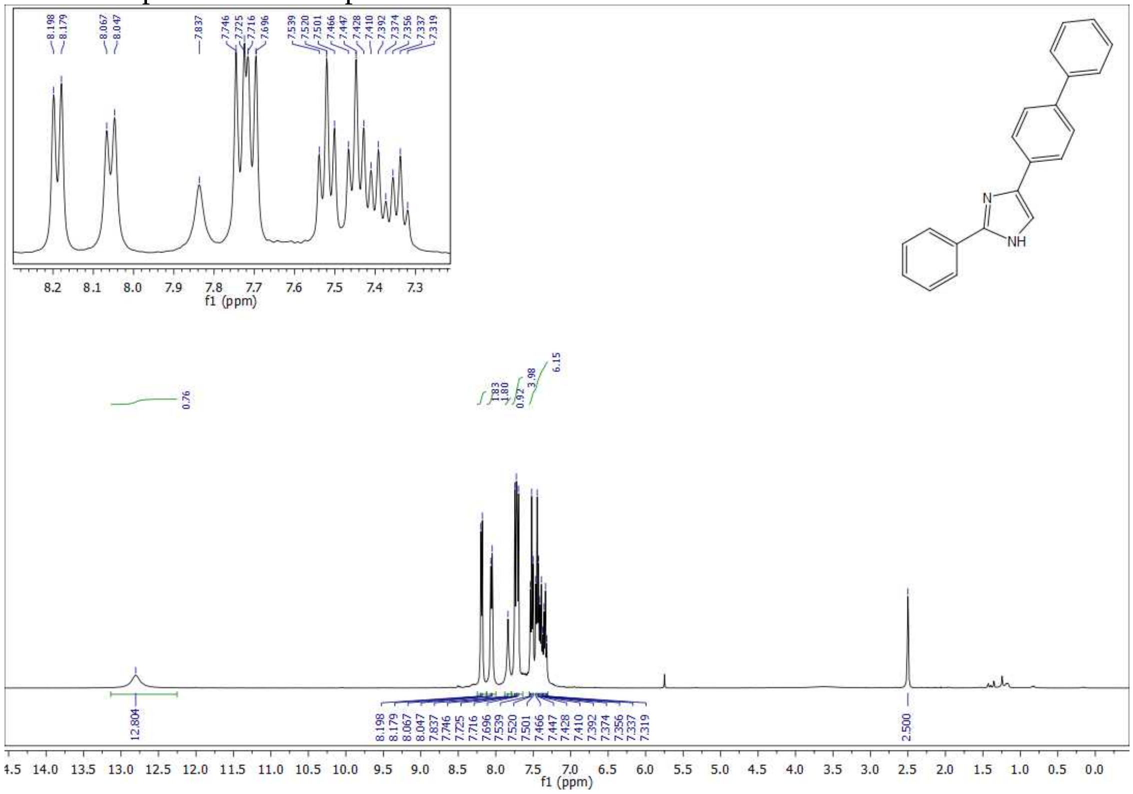

^{13}C NMR spectrum of compound **3ah**

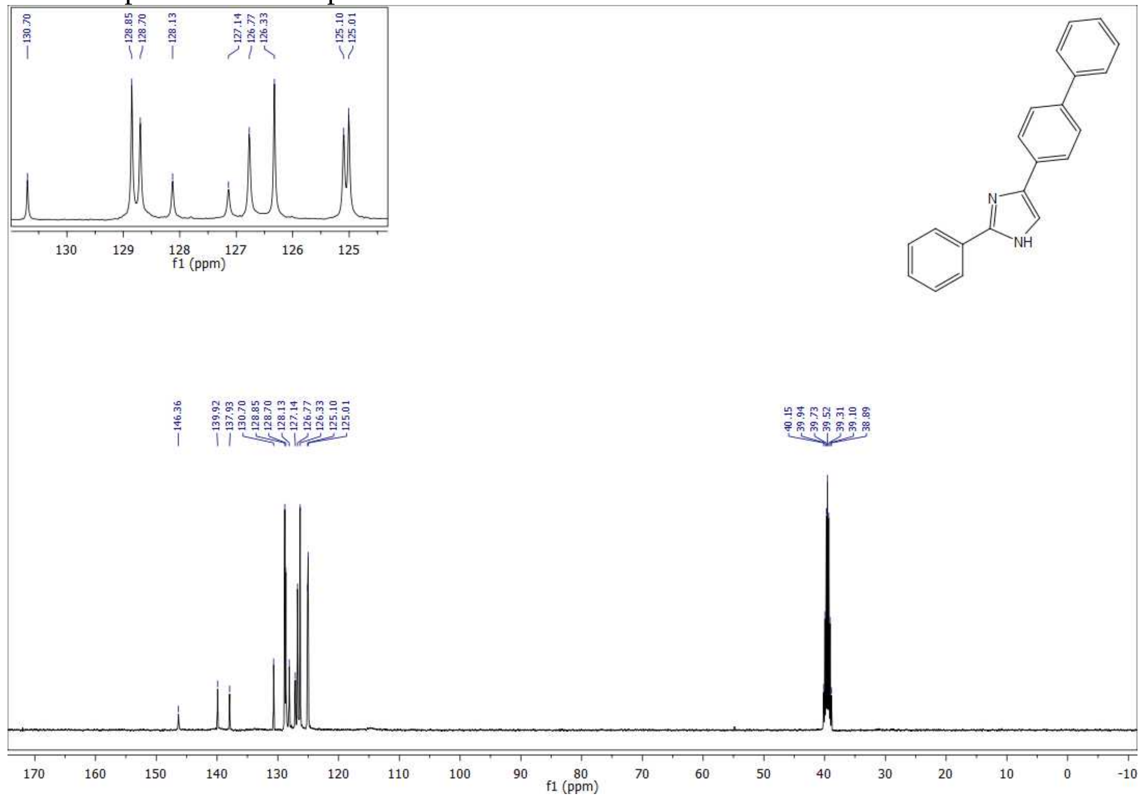

^{1}H NMR spectrum of compound **3ai**

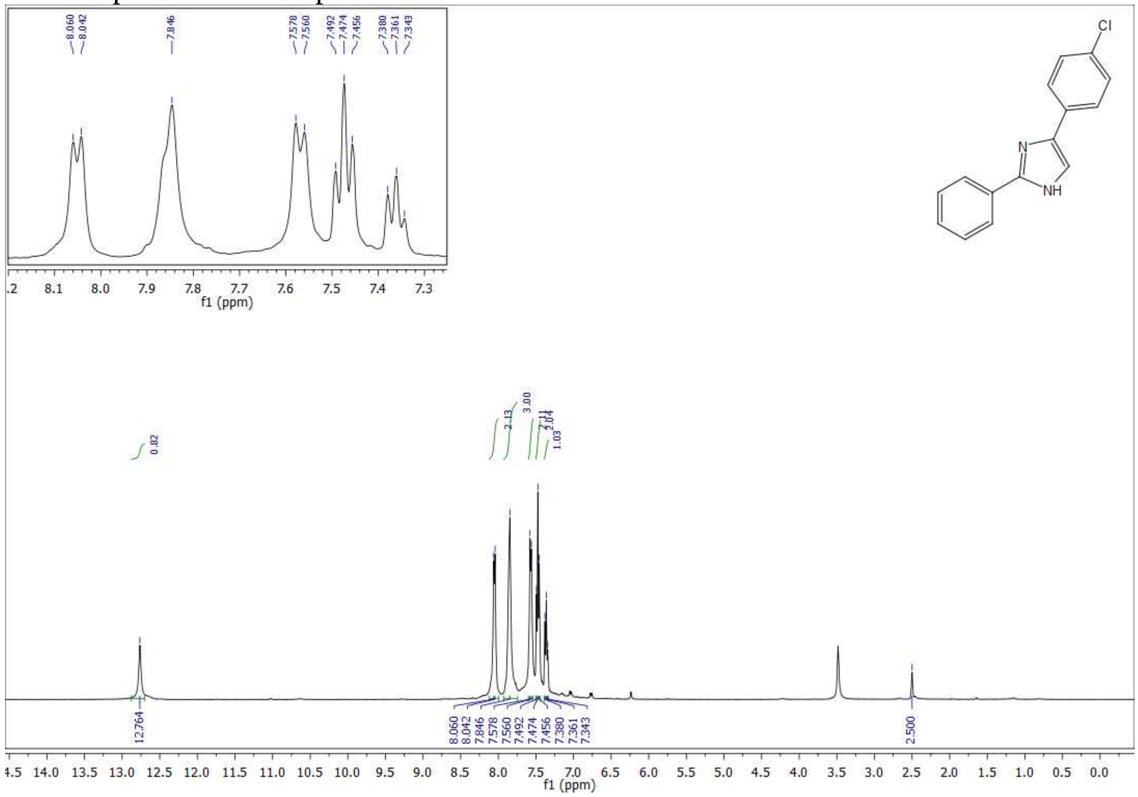

^{13}C NMR spectrum of compound **3ai**

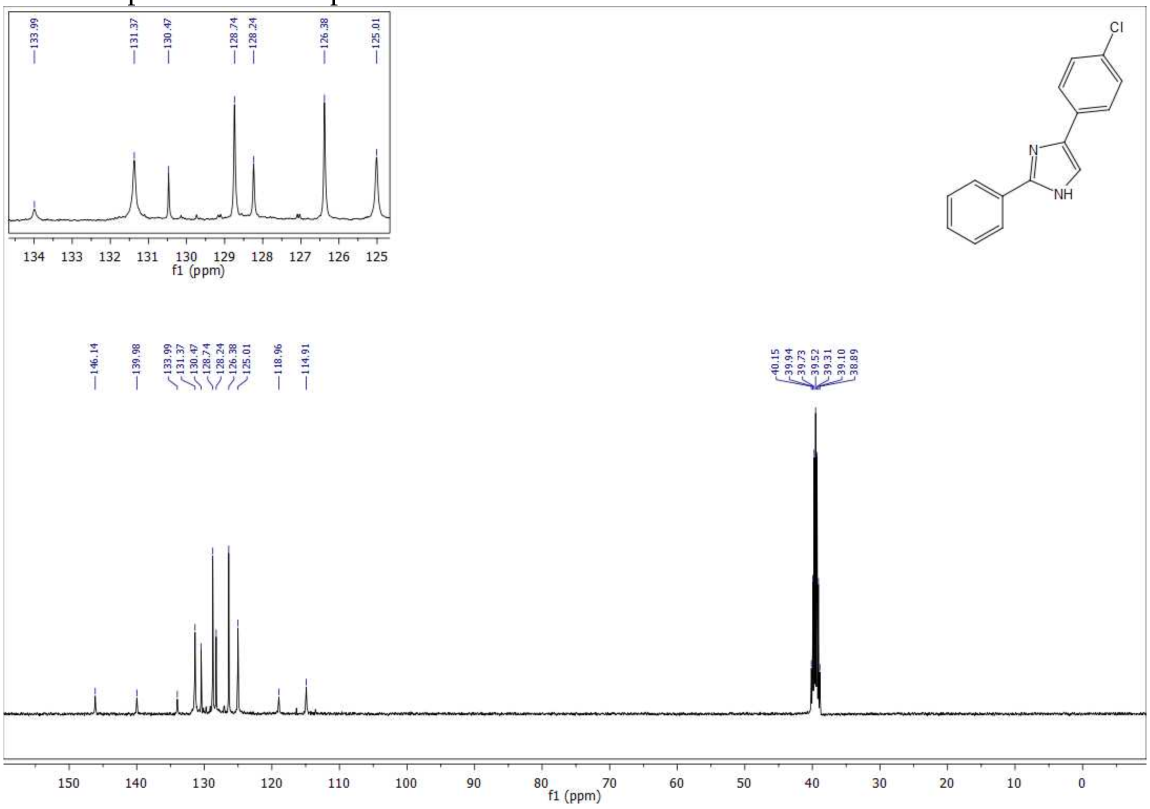

^{1}H NMR spectrum of compound **3aj**

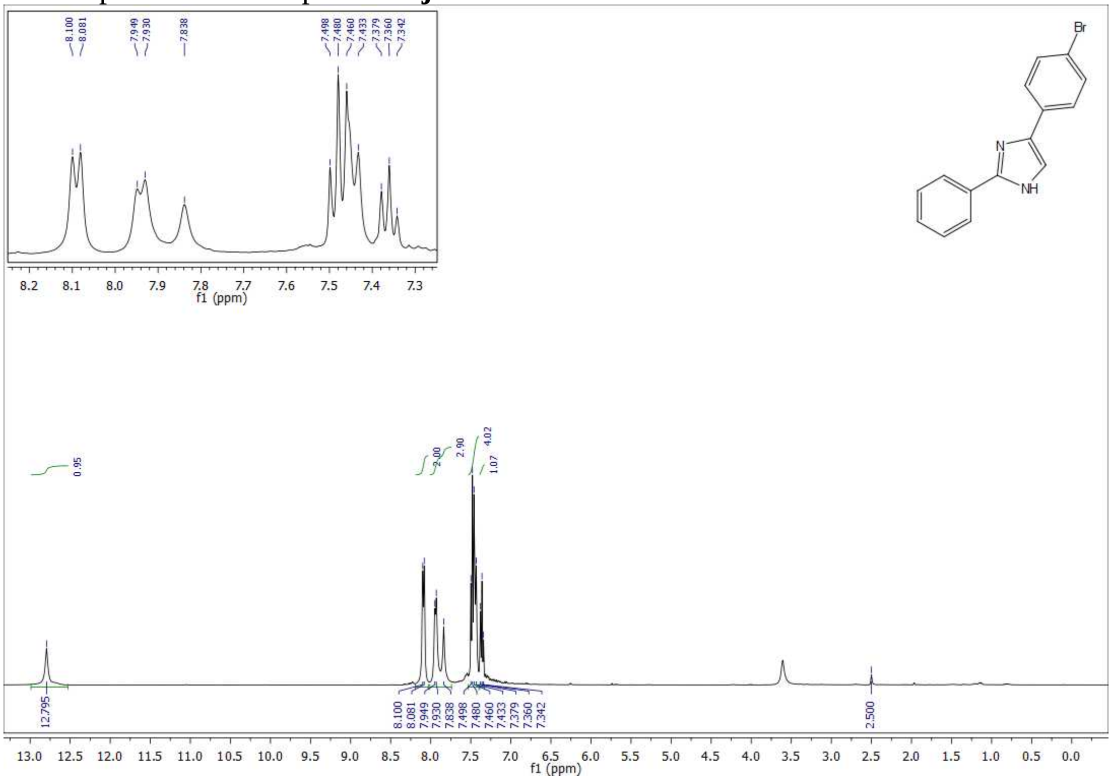

^{13}C NMR spectrum of compound **3aj**

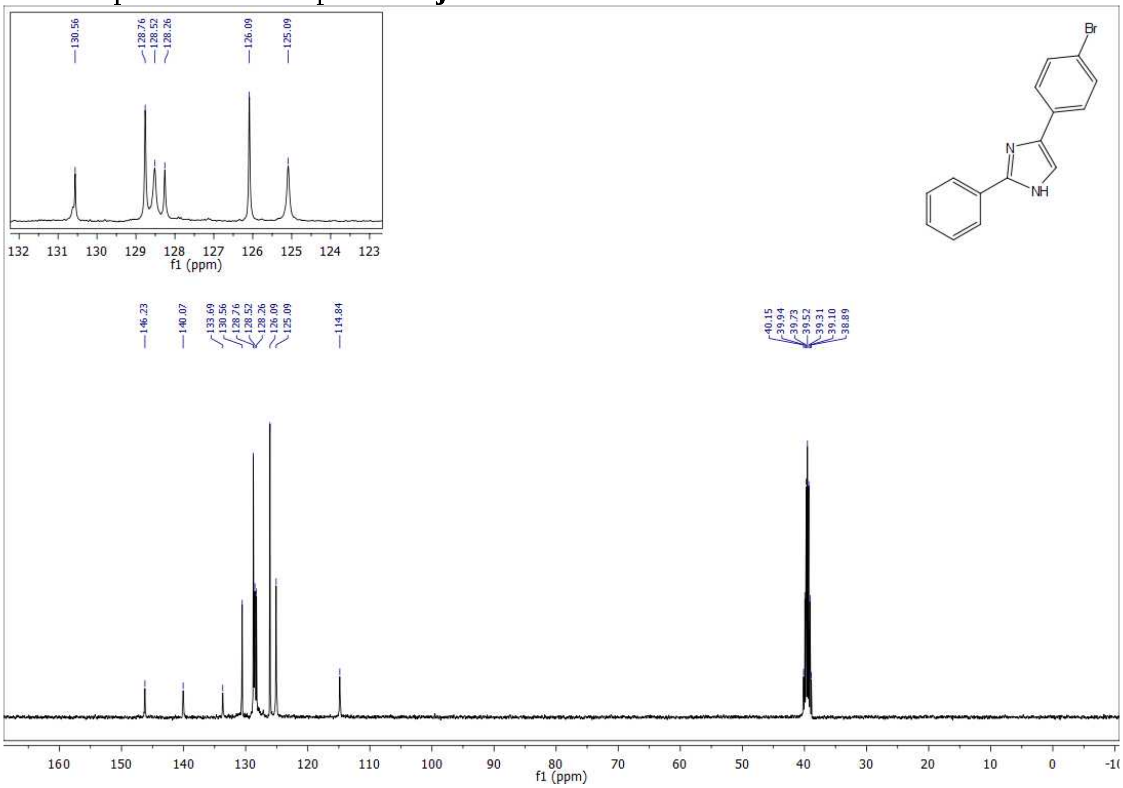

^{1}H NMR spectrum of compound **3ak**

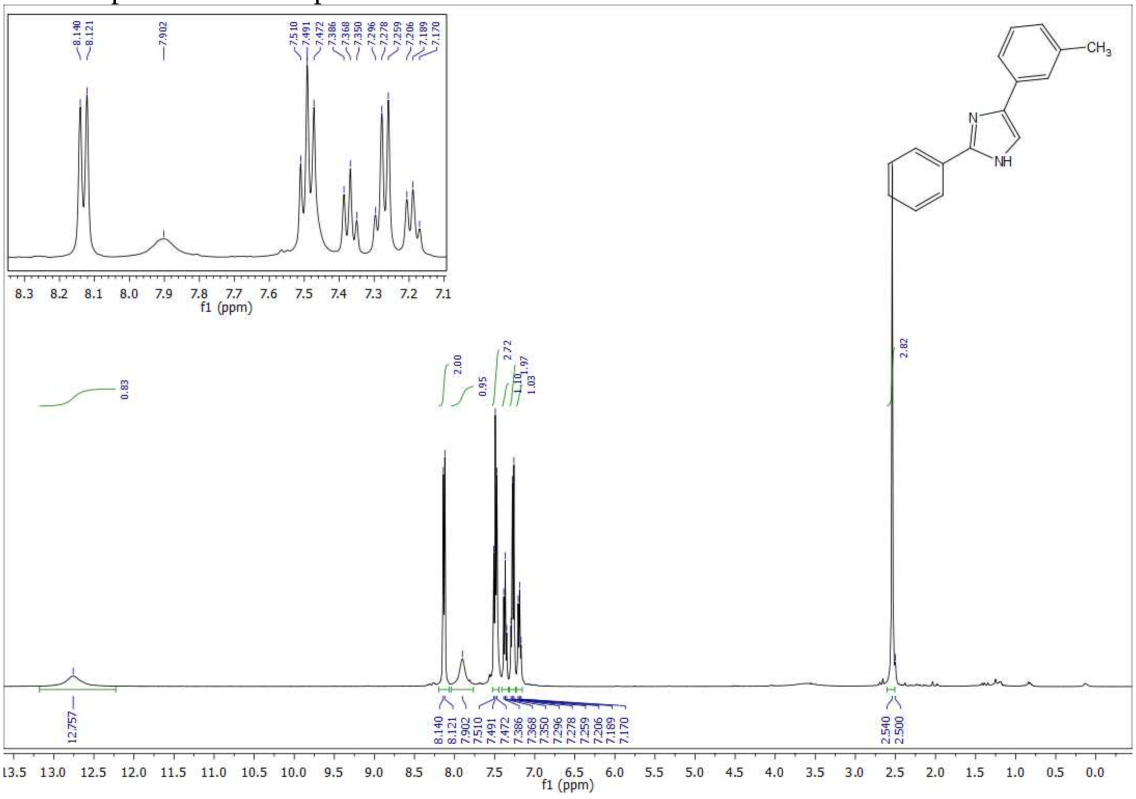

^{13}C NMR spectrum of compound **3ak**

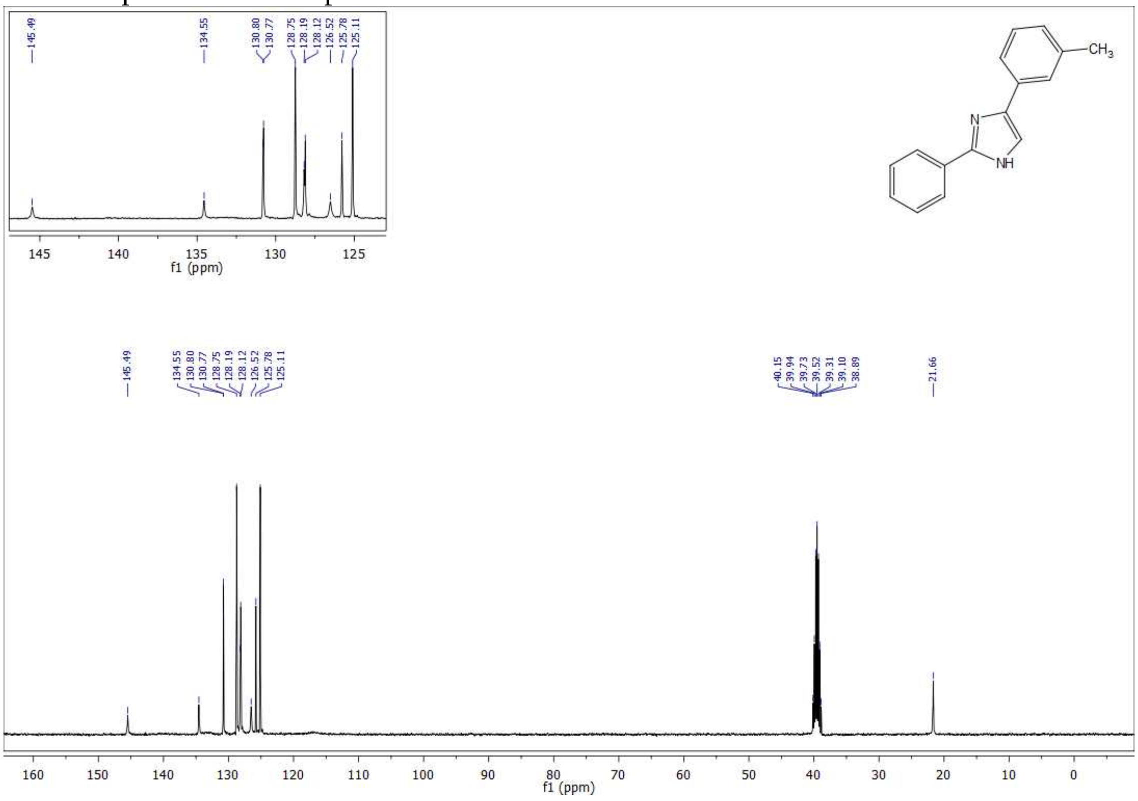

^{1}H NMR spectrum of compound **3al**

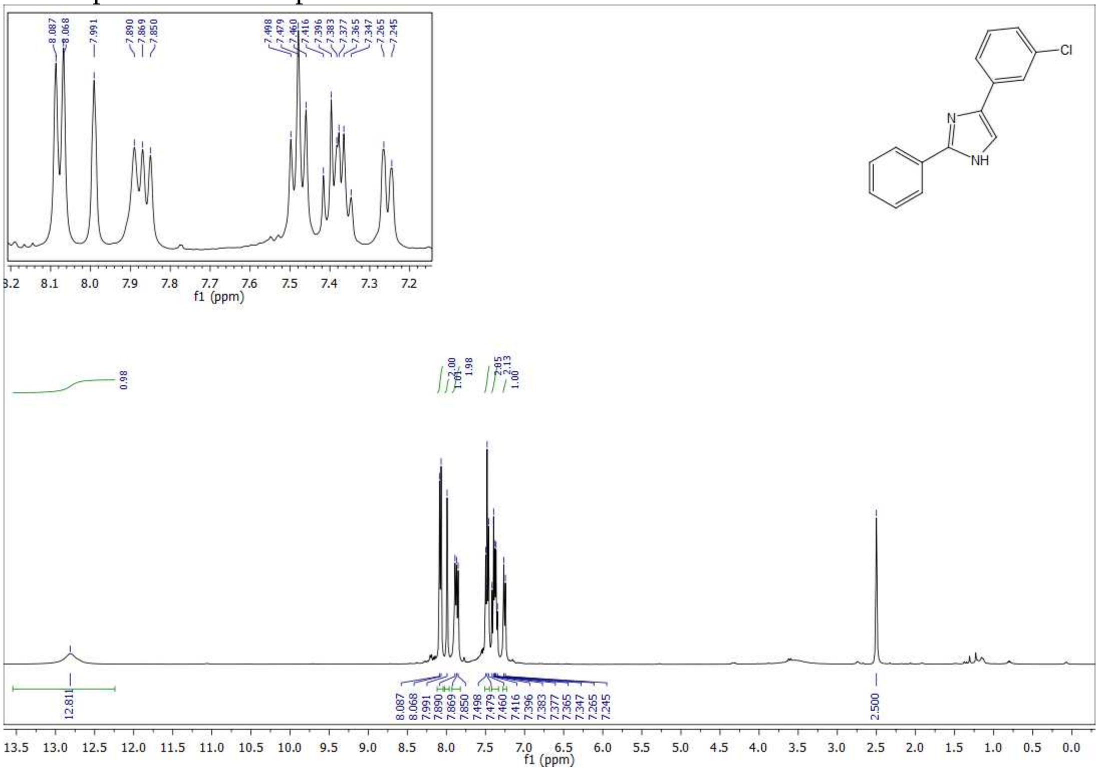

^{13}C NMR spectrum of compound **3al**

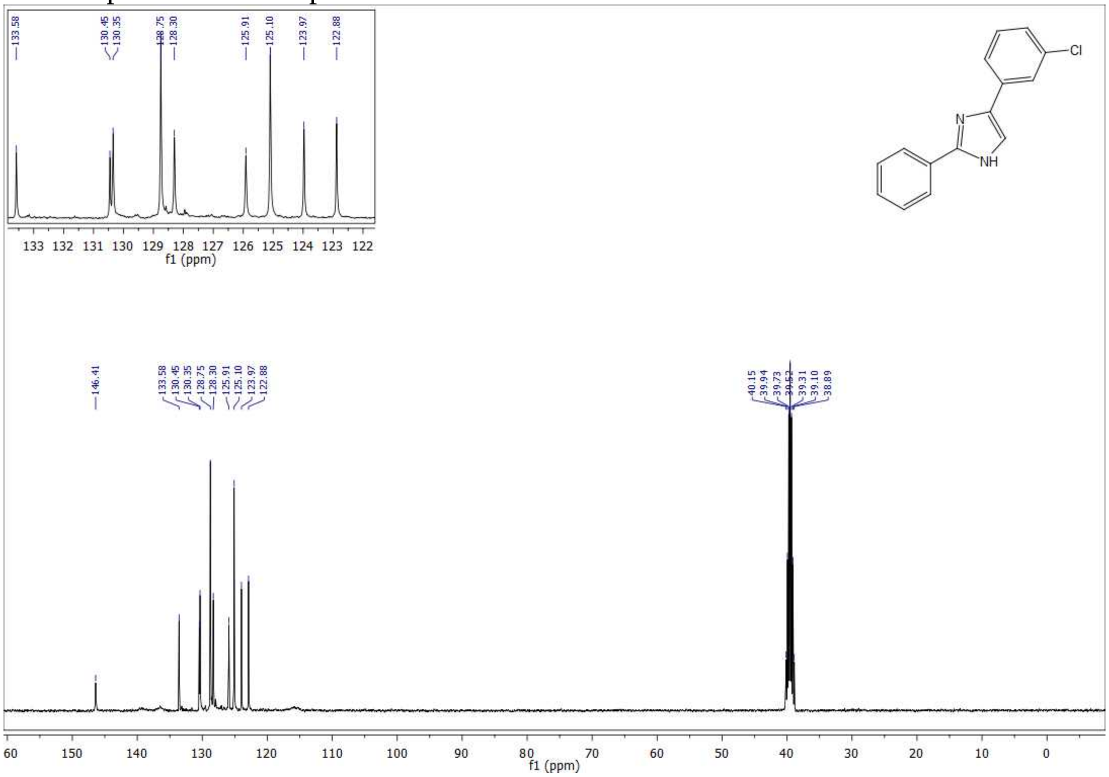

^{1}H NMR spectrum of compound **3am**

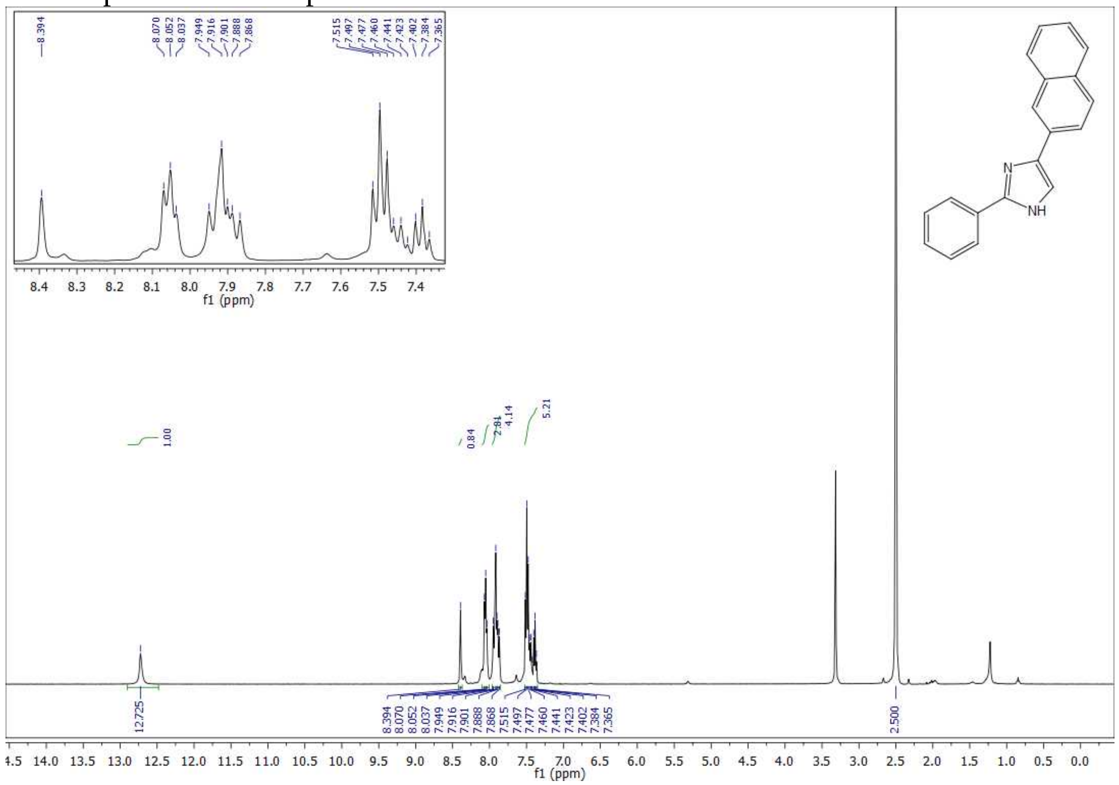

^{13}C NMR spectrum of compound **3am**

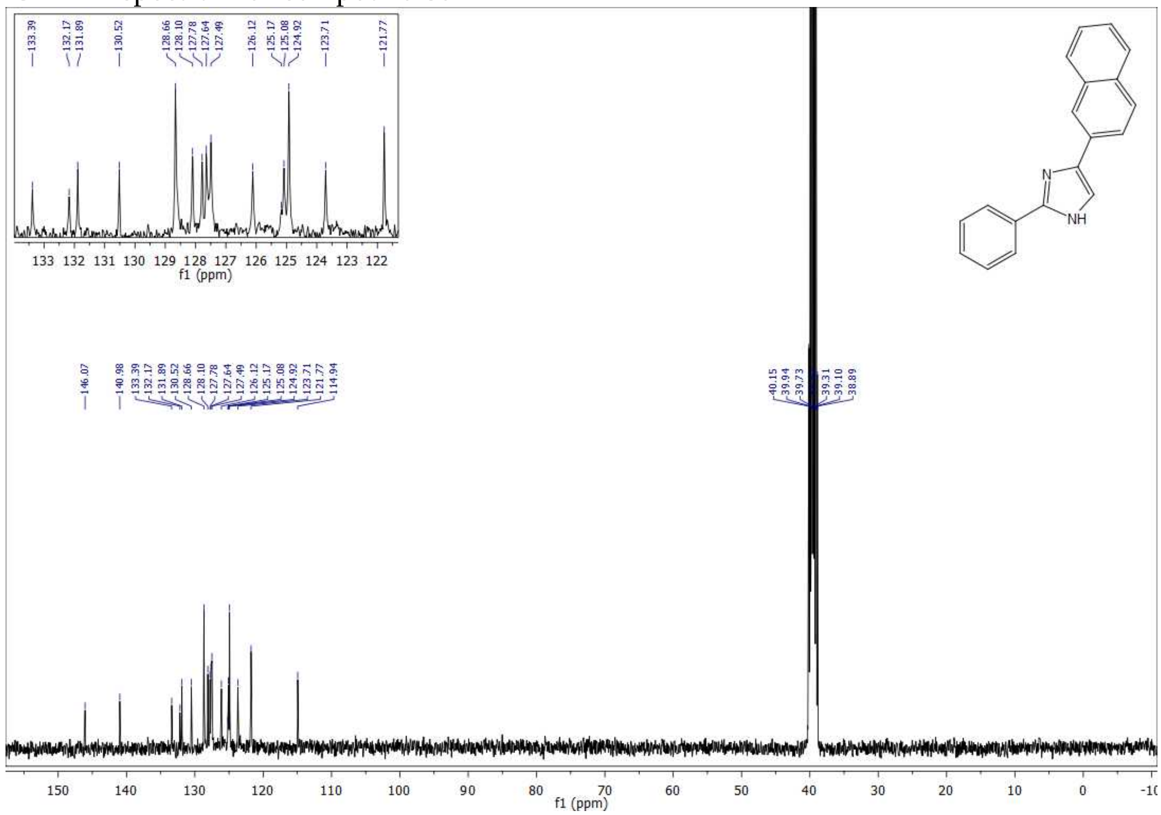

^{1}H NMR spectrum of compound **3an**

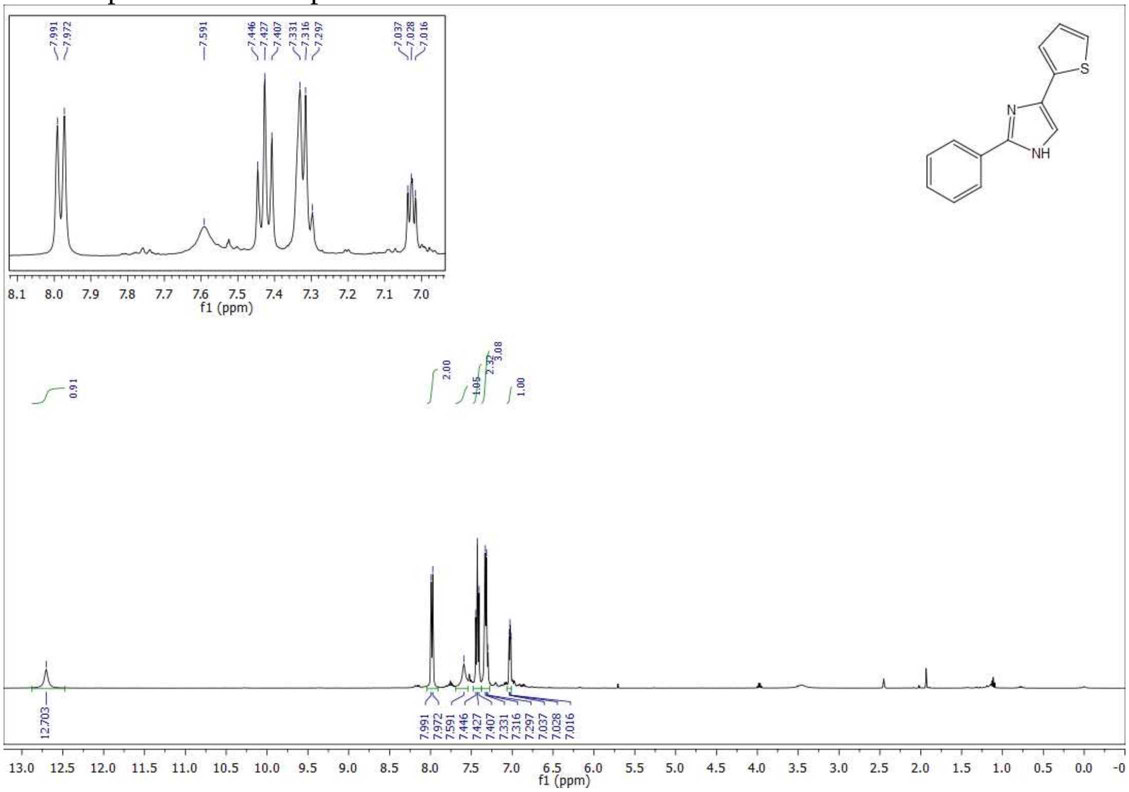

^{13}C NMR spectrum of compound **3an**

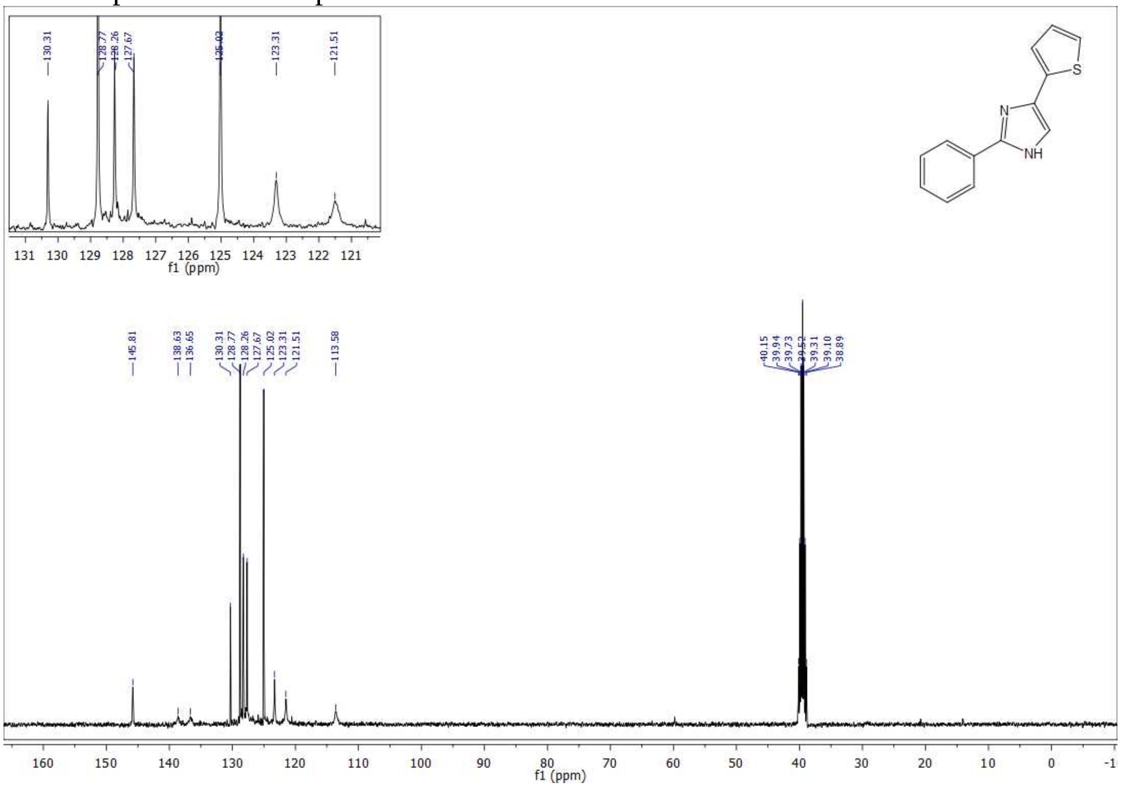

^{1}H NMR spectrum of compound **3ao**

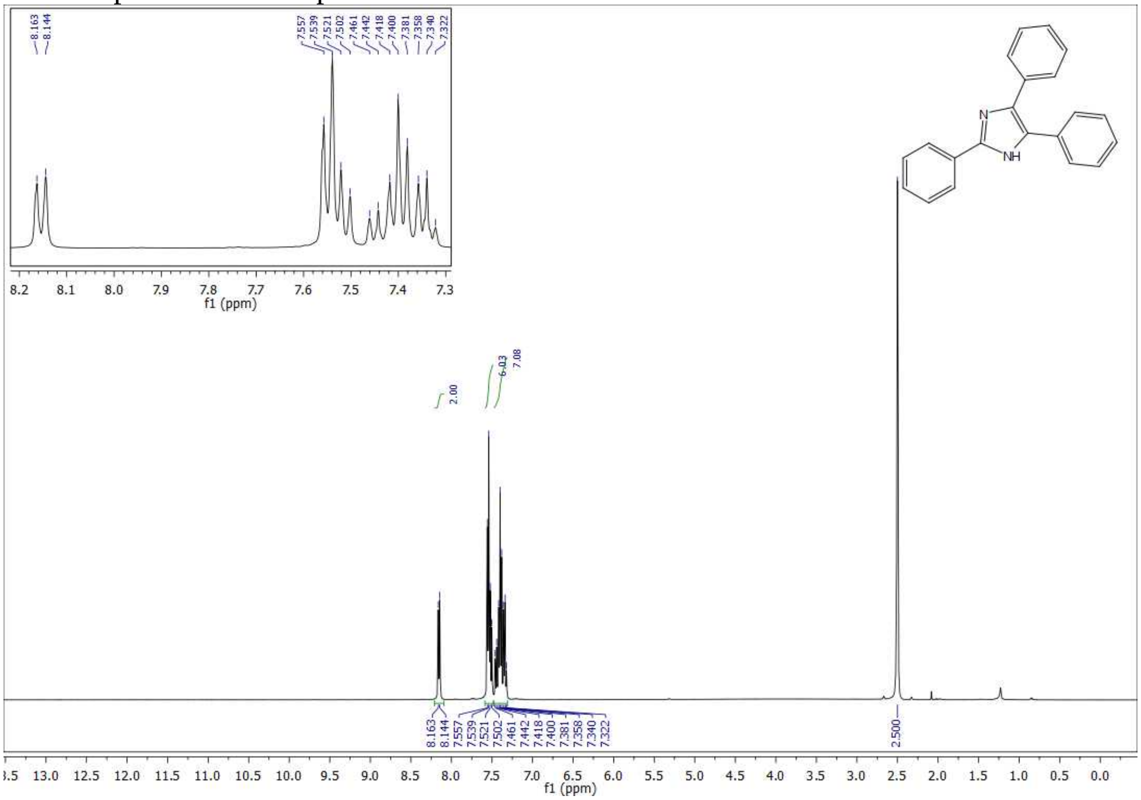

^{13}C NMR spectrum of compound **3ao**

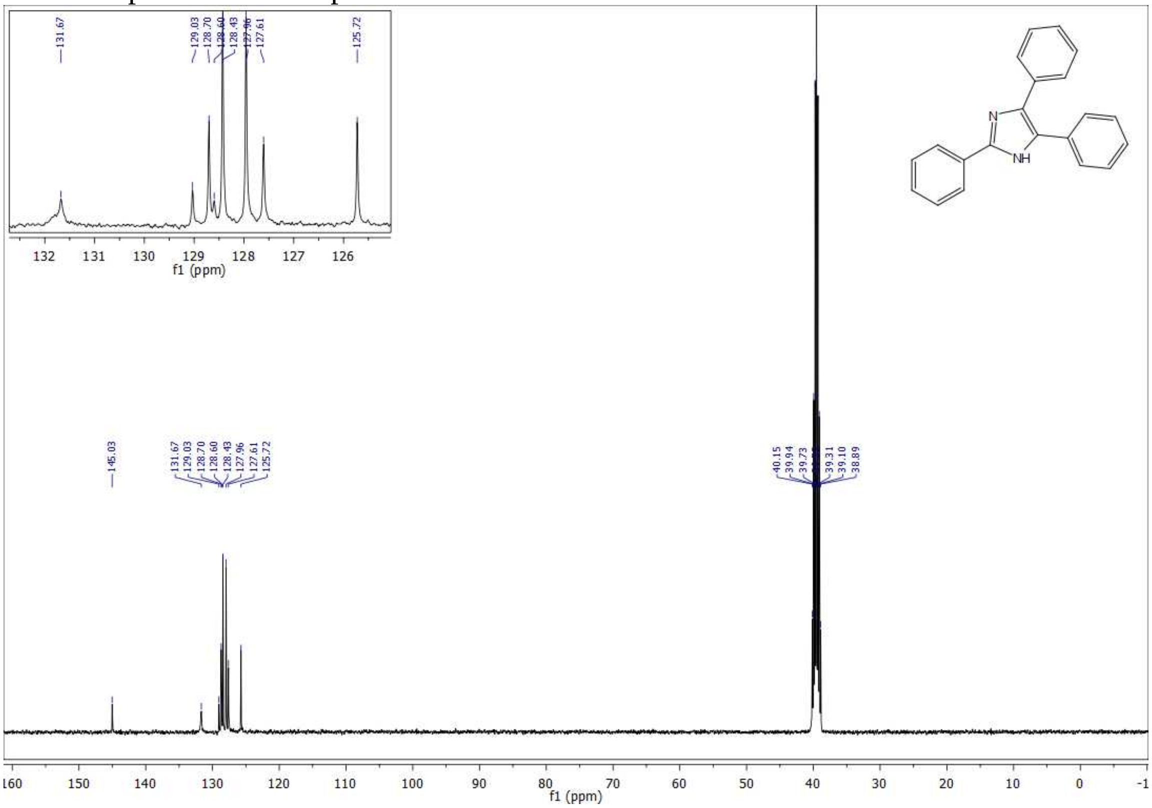

^{1}H NMR spectrum of compound **3ap**

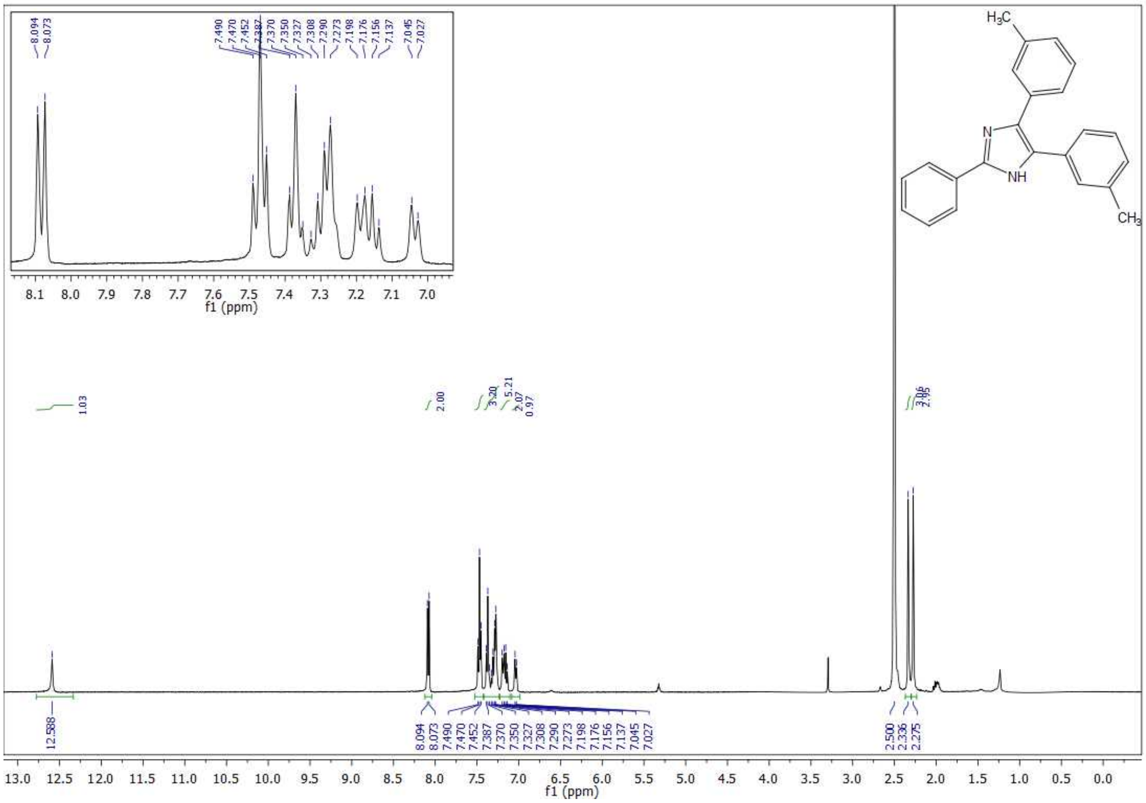

^{13}C NMR spectrum of compound **3ap**

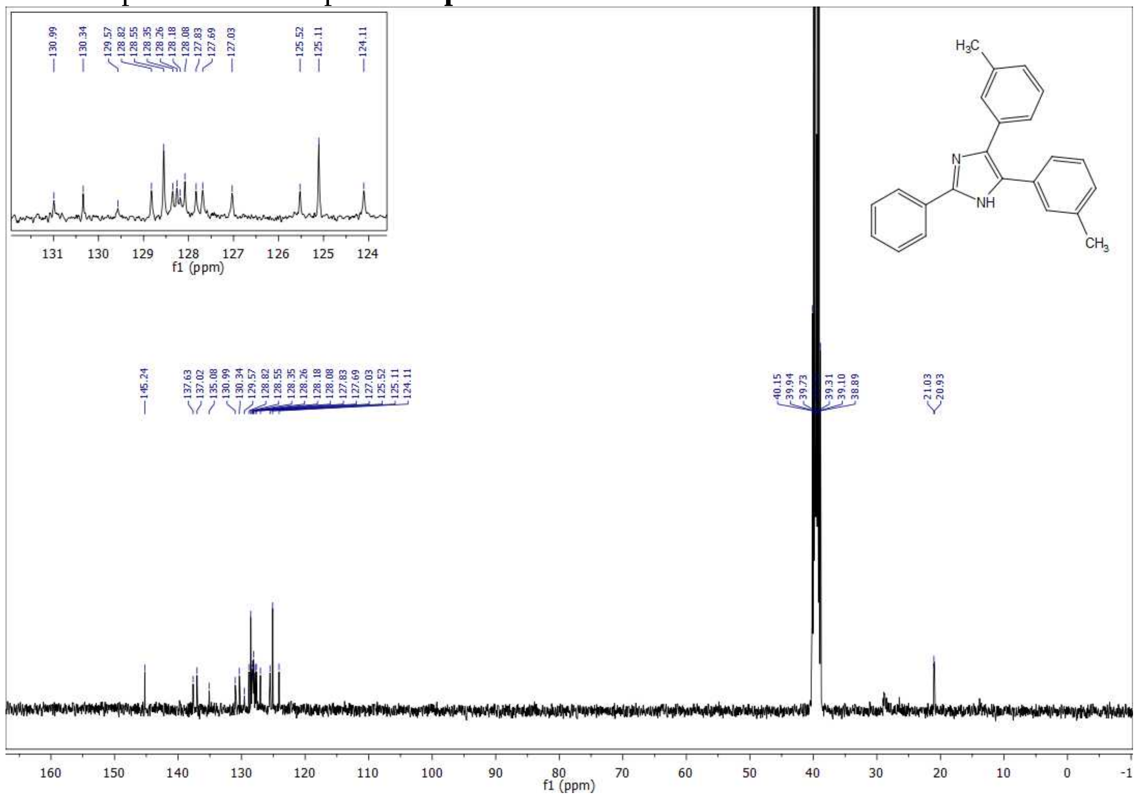

^{1}H NMR spectrum of compound **3aq**

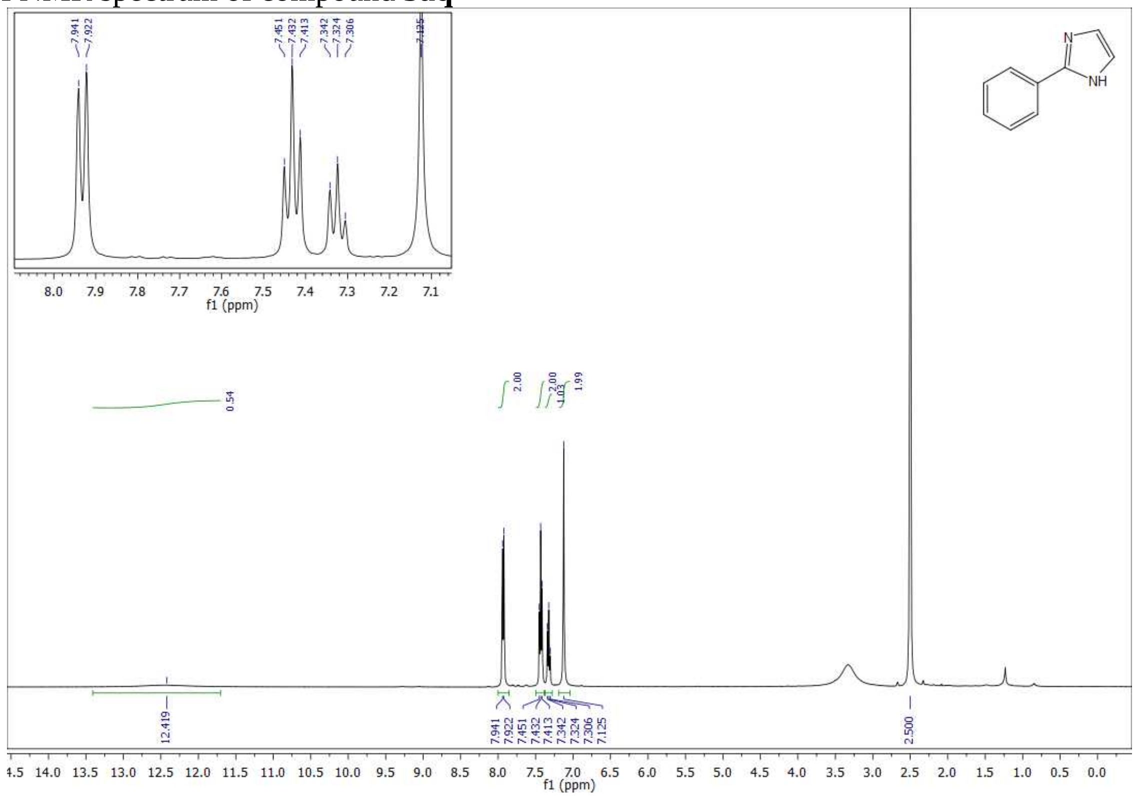

^{13}C NMR spectrum of compound **3aq**

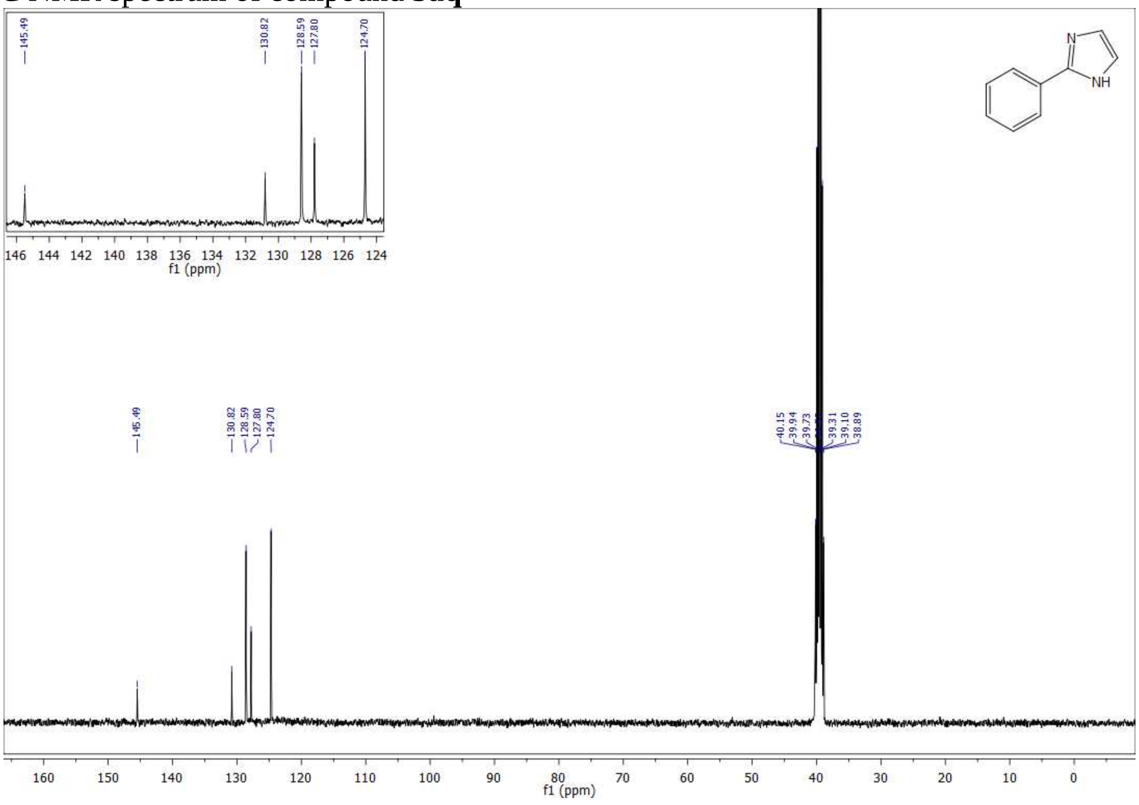

^{1}H NMR spectrum of compound **3ar**

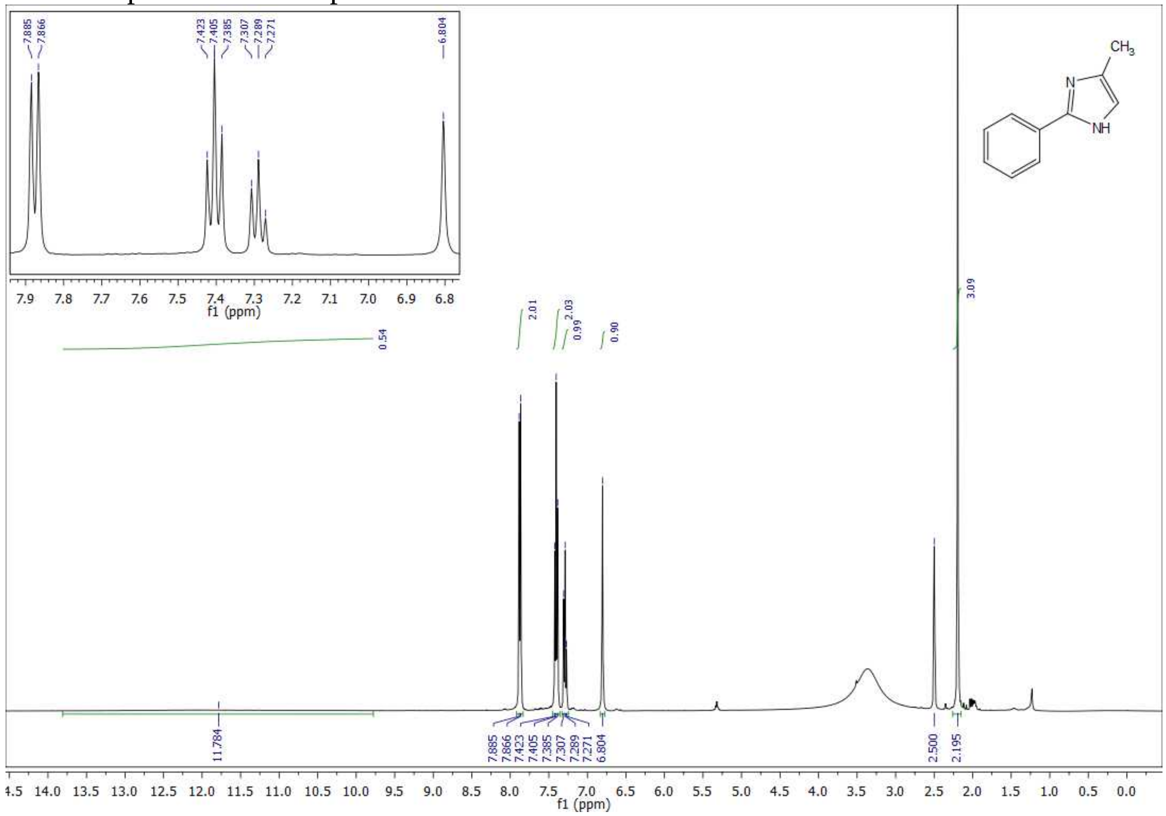

^{13}C NMR spectrum of compound **3ar**

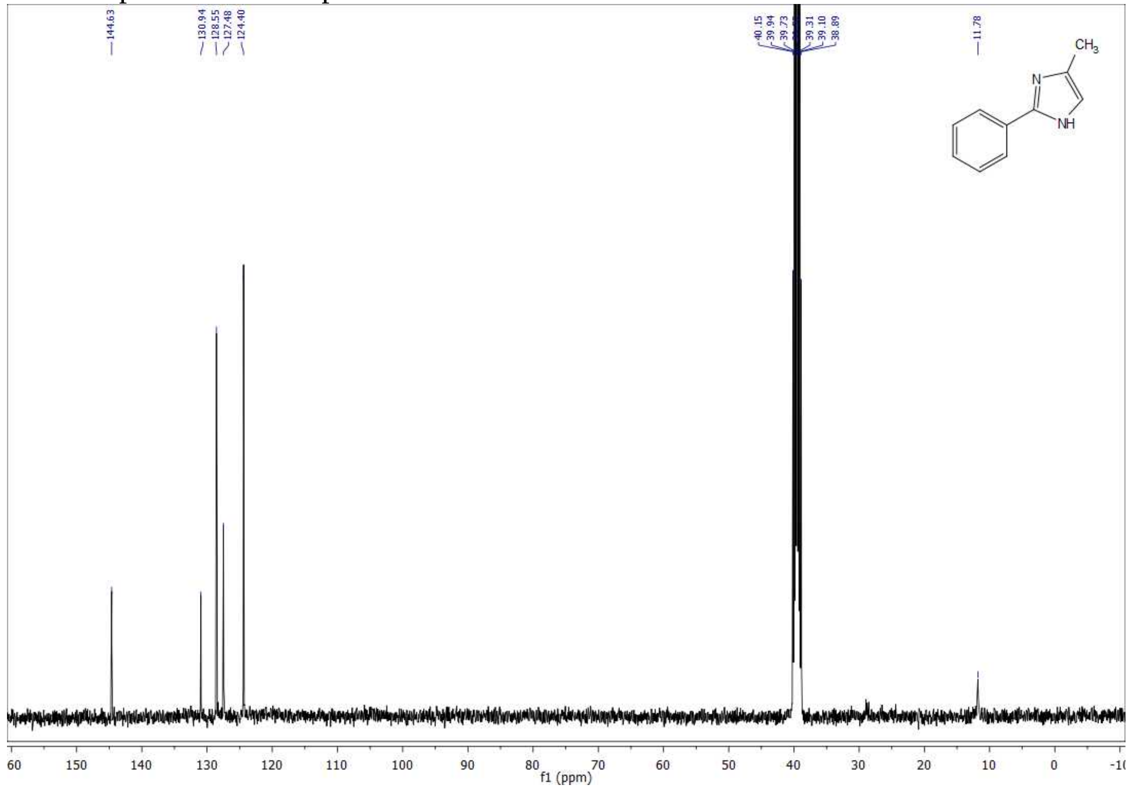

^{1}H NMR spectrum of compound **3ba**

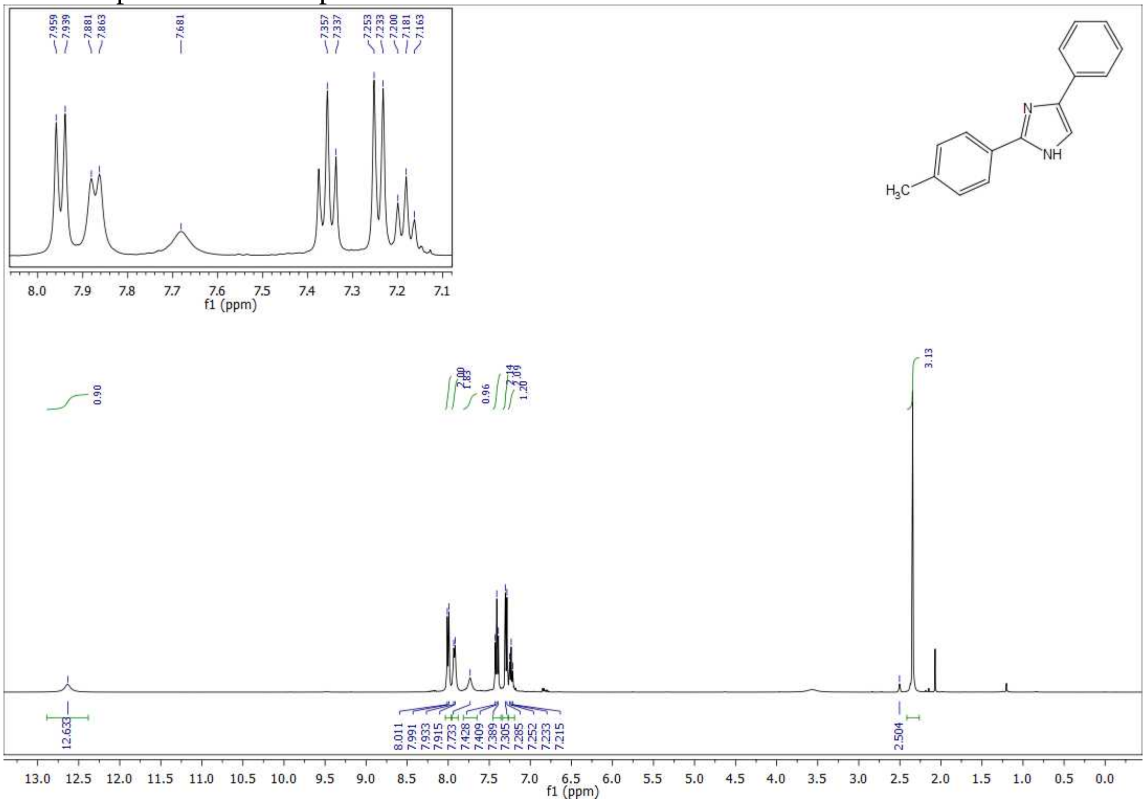

^{13}C NMR spectrum of compound **3ba**

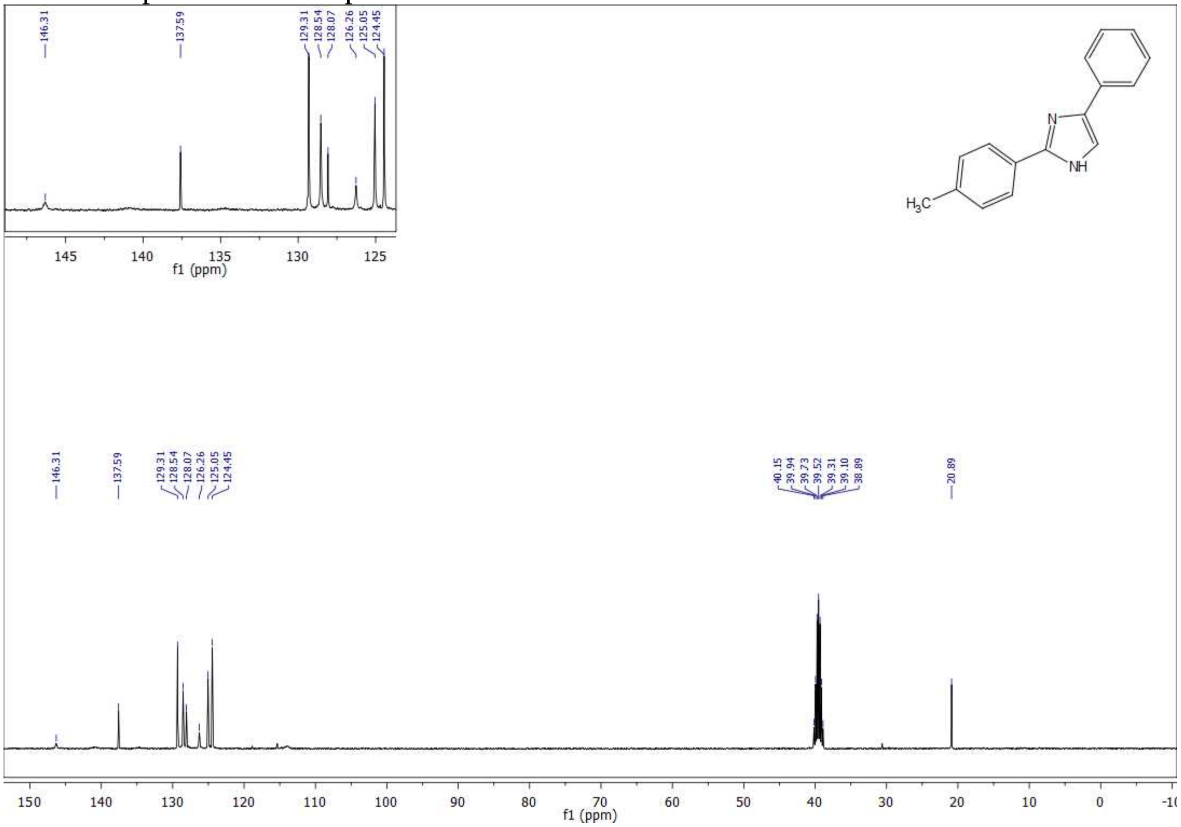

^{1}H NMR spectrum of compound **3ca**

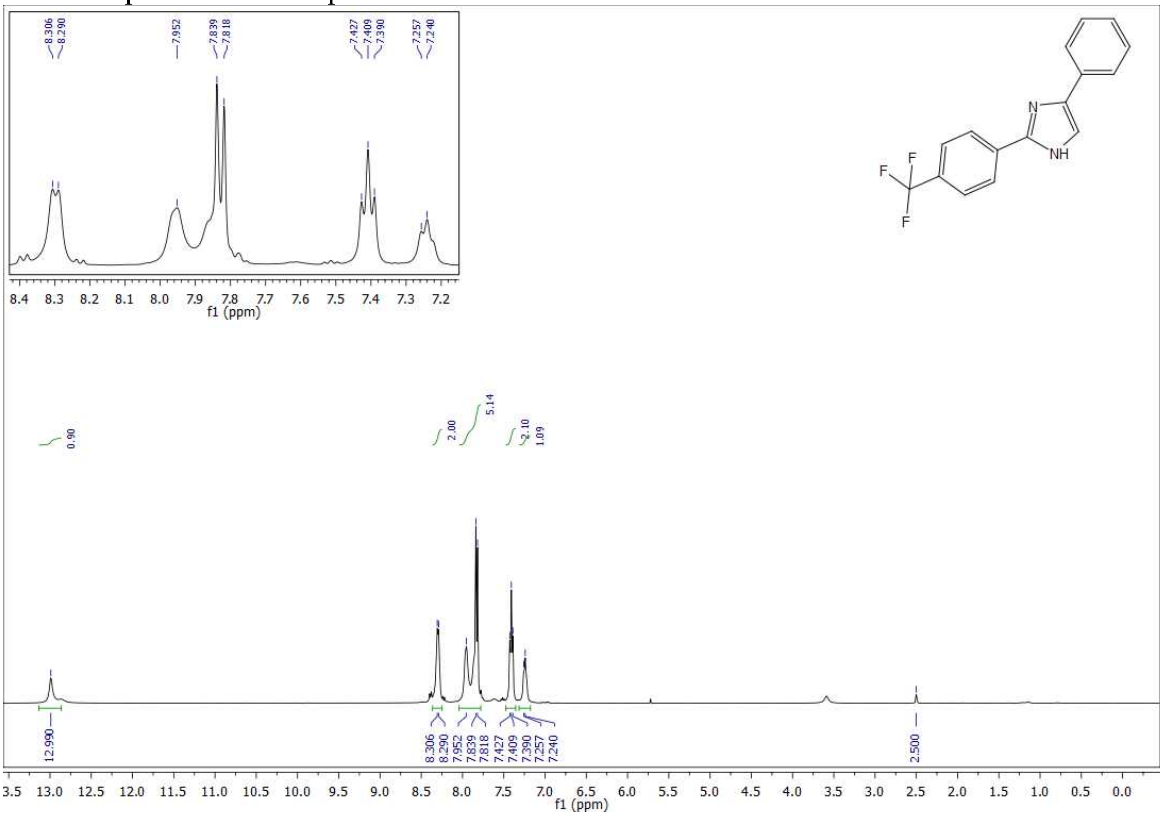

^{13}C NMR spectrum of compound **3ca**

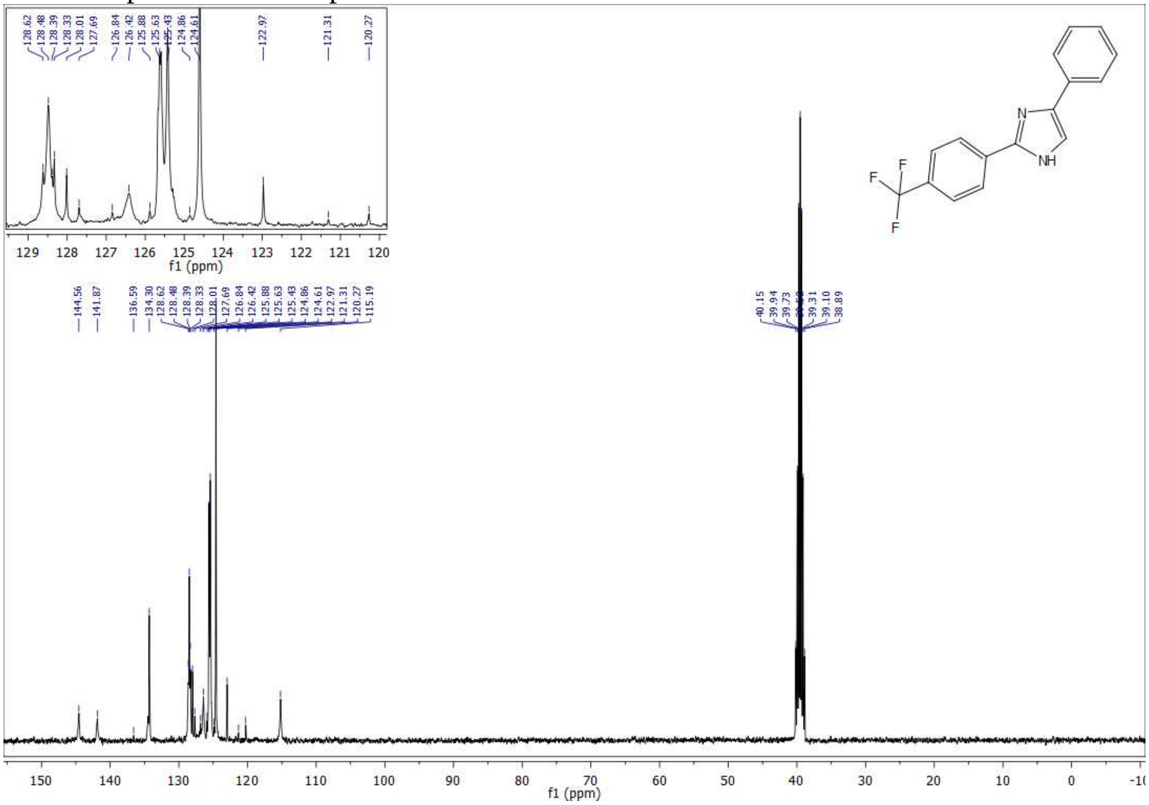

^{1}H NMR spectrum of compound **3da**

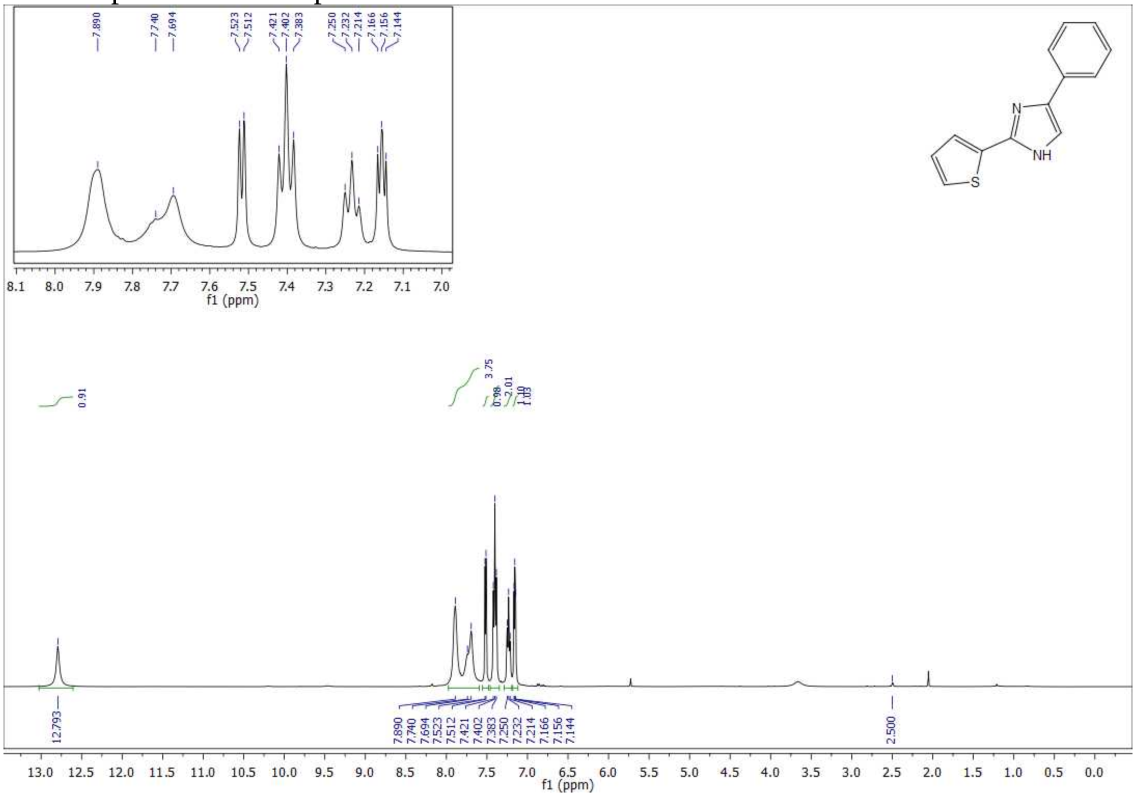

^{13}C NMR spectrum of compound **3da**

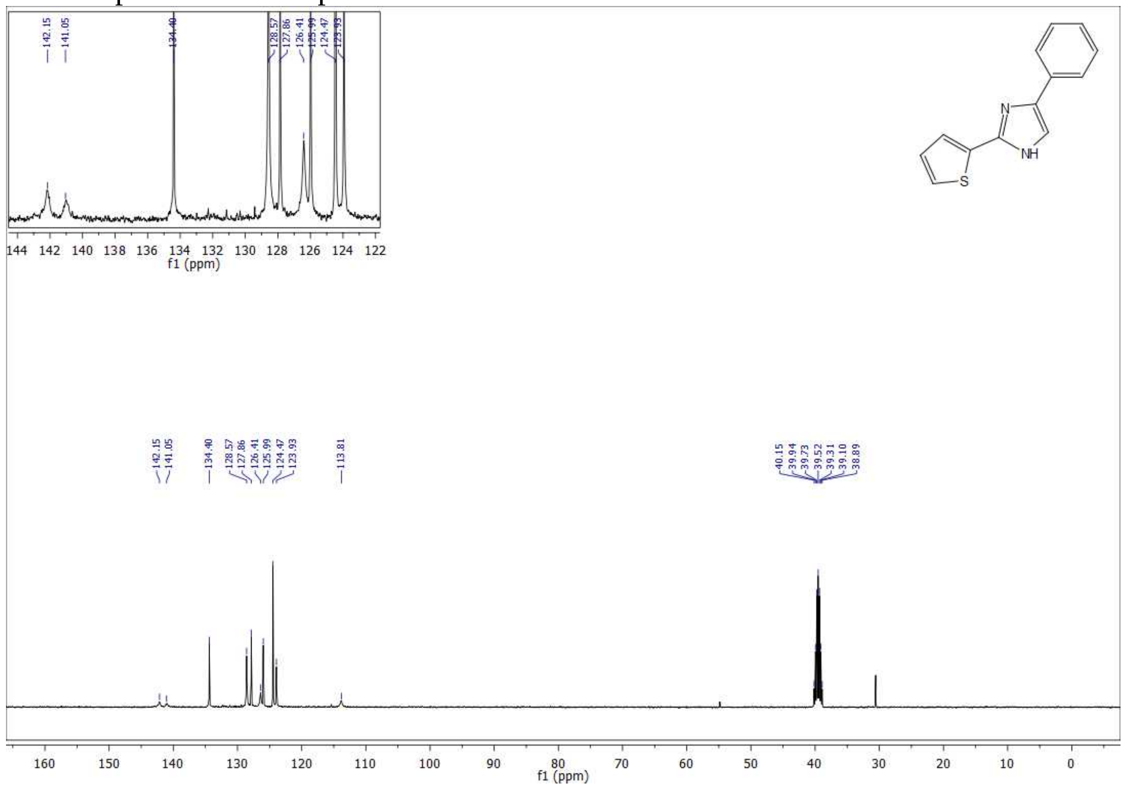

^{1}H NMR spectrum of compound **3ed**

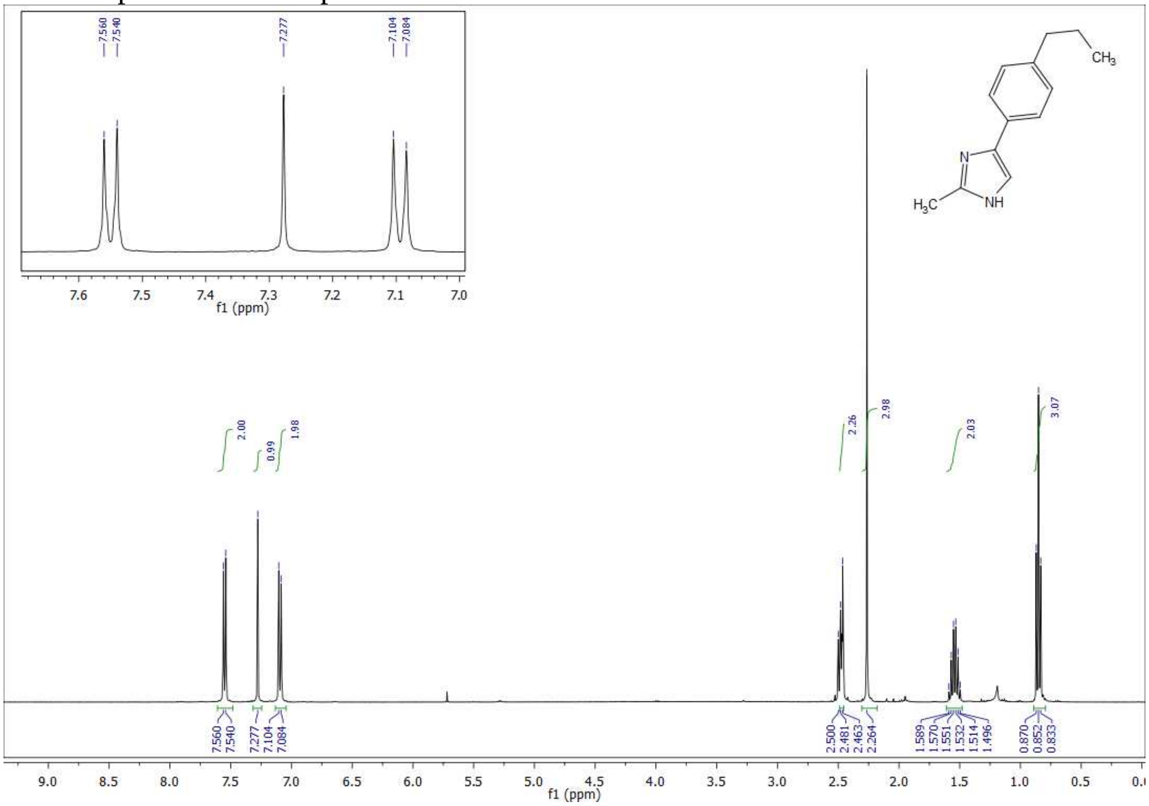

^{13}C NMR spectrum of compound **3ed**

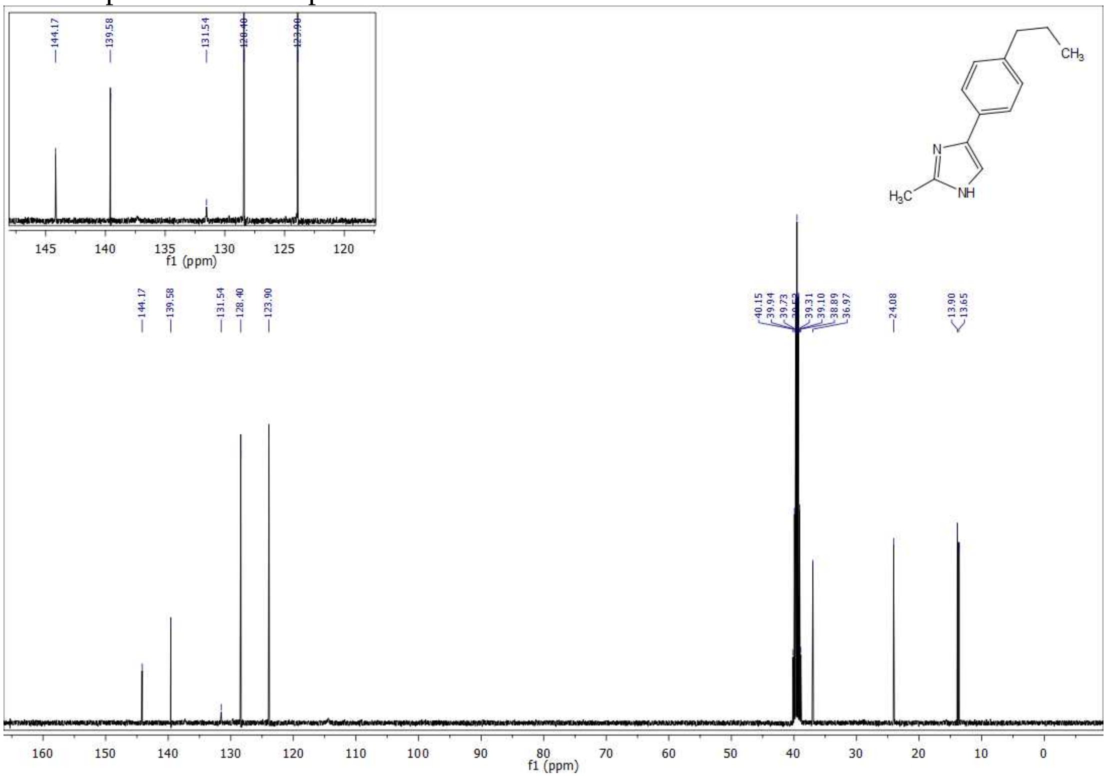

3. X-ray structural details of **3aa**

Crystal Structure of $(C_{15}H_{12}N_2)_6 \cdot C_2H_5OH$

The low temperature [100(2)°K] single-crystal X-ray experiments were performed on a SuperNova diffractometer with Cu K_α radiation. Unit cell was obtained and refined by 2642 reflections with 3.9° < θ < 70.9°. No decay was observed in data collection. Raw intensities were corrected for Lorentz and polarization effects, and for absorption by empirical method. Direct phase determination yielded the positions of all non-hydrogen atoms. All non-hydrogen atoms were subjected to anisotropic refinement. The hydrogen atoms were generated geometrically with C-H bonds of 0.93 Å according to criteria described in the SHELXTL manual (Bruker, 1997). They were included in the refinement with $U_{iso}(H) = 1.2U_{eq}$ of their parent atoms. The solvent molecule, C_2H_5OH, was disordered. It distributed in 6 possible orientations to give the statistic symmetry. The final full-matric least-square refinement on F^2 converged with $R1 = 0.0468$ and $wR2 = 0.1205$ for 2265 observed reflections [$I \geq 2\sigma(I)$]. The final difference electron density map shows no features. Details of crystal parameters, data collection and structure refinement are given in Table 1.

Data collection was controlled by CrysAlis^{Pro} (Rigaku, 2016). Computations were performed using the SHELXTL NT ver. 5.10 program package (Bruker, 1997) on an IBM PC 586 computer. Analytic expressions of atomic scattering factors were employed, and anomalous dispersion corrections were incorporated (*International Tables for X-ray Crystallography*, 1989). Crystal drawings were produced with XP (Bruker, 1997).

References

Bruker. (1997) SHELXTL. Structure Determination Programs, Version 5.10, Bruker AXS Inc.,6300 Enterprise Lane, Madison, WI 53719-1173, USA.

International Tables for X-ray Crystallography: (1989) Vol. C (Kluwer Academic Publishers, Dordrecht) Tables 4.2.6.8 and 6.1.1.4.

Rigaku. (2016) CrysAlis^{Pro}, Data Collection and Process Software for Rigaku Oxford Diffraction X-ray Diffractometer, Version 5.4, February, 2016. Rigaku Corporation, 9009, New Trails Drive, The Woodlands, TX77381, USA.

Table 1. Details of Data Collection, Processing and Structure Refinement

Sample code	20171114-2-**3aa**
Molecular formula	$(C_{15}H_{12}N_2)_6 \cdot C_2H_5OH$
Molecular weight	1367.66
Color and habit	colorless needle
Crystal size	0.10 × 0.15 × 0.25 mm
Crystal system	trigonal
Space group	$R\bar{3}$ (No. 148)
Unit cell parameters	a = 21.6938(3) Å α = 90.00°
	b = 21.6938(3) Å β = 90.00°
	c = 14.0946(3) Å γ = 120.00°
	V = 5744.5(2) Å^3 Z = 3 F(000) = 2166
Density (calcd)	1.186 g/cm^3
Diffractometer	SuperNova, Dual, Cu at zero, AtlasS2
Radiation	Cu K_α, λ = 1.54178 Å
Temperature	100(2)°K
Scan type	ω-scan
Data collection range	$-23 < h < 24$, $-18 < k < 23$, $-16 < l < 15$; θ_{max} = 71.2°
Reflections measured	Total: 4417 Unique (n): 2407 Observed [I ≥ 2σ(I)]: 2265
Absorption coefficient	0.559 mm^{-1}
Minimum and maximum transmission	0.782, 1.000
No. of variables, p	173
Weighting scheme	$w = \frac{1}{\sigma^2(F_o^2)+(0.05P)^2+5.0P}$ $P = (F_o^2 + 2F_c^2)/3$
$R1 = \frac{\Sigma\|\|F_o\|-\|F_c\|\|}{\Sigma\|F_o\|}$ (for all reflections)	0.0488 0.0468
$wR2 = \sqrt{\frac{\Sigma[w(F_o^2-F_c^2)^2]}{\Sigma w(F_o^2)^2}}$ (for all reflections)	0.1221 0.1205 (for observed data)
Goof = S = $\sqrt{\frac{\Sigma[w(F_o^2-F_c^2)^2]}{n-p}}$	1.265
Largest and mean Δ/σ	0.001, 0.000
Residual extrema in final difference map	-0.597 to 0.484 e Å^{-3}

Table 2. Atomic coordinates and equivalent isotropic temperature factors* (Å^2)

Atoms	*x*	*y*	*z*	$U_{eq.}$	*Occupancy*
N(1)	0.63185(6)	0.95833(6)	0.04498(8)	0.0258(3)	1.0000
N(2)	0.66306(6)	0.94876(6)	0.19129(8)	0.0245(3)	1.0000
C(1)	0.68537(7)	0.98413(7)	0.10956(9)	0.0239(3)	1.0000
C(2)	0.59218(7)	0.89840(7)	0.17758(9)	0.0246(3)	1.0000
C(3)	0.57273(8)	0.90430(7)	0.08703(9)	0.0268(3)	1.0000
C(4)	0.75792(7)	1.04258(7)	0.09173(9)	0.0254(3)	1.0000
C(5)	0.77487(8)	1.08569(8)	0.01141(10)	0.0295(3)	1.0000
C(6)	0.84376(8)	1.14087(8)	-0.00290(11)	0.0348(4)	1.0000
C(7)	0.89653(9)	1.15401(9)	0.06226(12)	0.0398(4)	1.0000
C(8)	0.88017(9)	1.11155(10)	0.14246(12)	0.0422(4)	1.0000
C(9)	0.81176(8)	1.05616(9)	0.15666(11)	0.0337(4)	1.0000
C(10)	0.54866(8)	0.84925(7)	0.25315(10)	0.0268(3)	1.0000
C(11)	0.58040(8)	0.83669(8)	0.33115(10)	0.0318(3)	1.0000
C(12)	0.53903(10)	0.79001(8)	0.40228(11)	0.0400(4)	1.0000
C(13)	0.46587(10)	0.75536(8)	0.39674(12)	0.0435(4)	1.0000
C(14)	0.43376(9)	0.76792(9)	0.32023(13)	0.0421(4)	1.0000
C(15)	0.47454(8)	0.81459(8)	0.24881(11)	0.0337(4)	1.0000
O(1)	0.3333	0.6667	0.5452(3)	0.0581(12)	0.5000
C(16)	0.3333	0.6667	0.6321(5)	0.117(3)	0.5000
C(17)	0.2845(8)	0.6934(10)	0.6704(7)	0.058(3)	0.1667

*$U_{eq.}$ defined as one third of the trace of the orthogonalized **U** tensor.

Table 3. Bond lengths (Å) and bond angles (º)

N(1)-C(1)	1.3565(17)	C(7)-C(8)	1.387(2)
N(1)-C(3)	1.3663(18)	C(8)-C(9)	1.380(2)
N(2)-C(1)	1.3338(17)	C(10)-C(11)	1.395(2)
N(2)-C(2)	1.3840(18)	C(10)-C(15)	1.395(2)
C(1)-C(4)	1.4671(19)	C(11)-C(12)	1.388(2)
C(2)-C(3)	1.3702(19)	C(12)-C(13)	1.377(3)
C(2)-C(10)	1.4684(19)	C(13)-C(14)	1.383(3)
C(4)-C(9)	1.394(2)	C(14)-C(15)	1.387(2)
C(4)-C(5)	1.3955(19)	O(1)-C(16)	1.224(7)
C(5)-C(6)	1.385(2)	C(16)-C(17)	1.538(7)
C(6)-C(7)	1.382(2)		
C(1)-N(1)-C(3)	107.79(11)	C(7)-C(6)-C(5)	120.41(14)
C(1)-N(2)-C(2)	105.90(11)	C(6)-C(7)-C(8)	119.61(15)
N(2)-C(1)-N(1)	110.65(12)	C(9)-C(8)-C(7)	120.21(15)
N(2)-C(1)-C(4)	124.69(12)	C(8)-C(9)-C(4)	120.75(14)
N(1)-C(1)-C(4)	124.66(12)	C(11)-C(10)-C(15)	118.56(13)
C(3)-C(2)-N(2)	109.20(12)	C(11)-C(10)-C(2)	120.84(13)
C(3)-C(2)-C(10)	128.85(13)	C(15)-C(10)-C(2)	120.60(13)
N(2)-C(2)-C(10)	121.95(12)	C(12)-C(11)-C(10)	120.63(15)
N(1)-C(3)-C(2)	106.46(12)	C(13)-C(12)-C(11)	120.37(16)
C(9)-C(4)-C(5)	118.61(13)	C(12)-C(13)-C(14)	119.55(15)
C(9)-C(4)-C(1)	119.56(12)	C(13)-C(14)-C(15)	120.62(16)
C(5)-C(4)-C(1)	121.83(13)	C(14)-C(15)-C(10)	120.27(15)
C(6)-C(5)-C(4)	120.41(14)	O(1)-C(16)-C(17)	110.5(4)
Hydrogen bonding			
H(1)···N(2)$^{\#1}$	1.95(2)	N(1)-H(1)···N(2)$^{\#1}$	170.8(18)

Symmetry transformation codes: #1(0.3333-*x*+*y*, 1.6667-*x*, -0.3333+*z*).

Table 4. Anisotropic thermal parameters* (Å^2)

Atoms	U_{11}	U_{22}	U_{33}	U_{23}	U_{13}	U_{12}
N(1)	0.0305(6)	0.0284(6)	0.0176(6)	0.0002(4)	-0.0017(4)	0.0139(5)
N(2)	0.0268(6)	0.0276(6)	0.0182(5)	-0.0004(4)	0.0005(4)	0.0129(5)
C(1)	0.0292(7)	0.0270(7)	0.0175(6)	-0.0023(5)	-0.0008(5)	0.0155(6)
C(2)	0.0267(7)	0.0264(7)	0.0210(6)	-0.0021(5)	0.0000(5)	0.0134(6)
C(3)	0.0277(7)	0.0287(7)	0.0220(7)	-0.0024(5)	-0.0025(5)	0.0127(6)
C(4)	0.0295(7)	0.0272(7)	0.0203(6)	-0.0017(5)	0.0016(5)	0.0147(6)
C(5)	0.0338(8)	0.0306(7)	0.0219(7)	0.0005(5)	-0.0017(5)	0.0144(6)
C(6)	0.0385(8)	0.0316(8)	0.0277(7)	0.0059(6)	0.0027(6)	0.0125(7)
C(7)	0.0303(8)	0.0393(9)	0.0384(9)	0.0079(7)	0.0025(6)	0.0087(7)
C(8)	0.0300(8)	0.0506(10)	0.0369(9)	0.0102(7)	-0.0037(6)	0.0133(7)
C(9)	0.0311(8)	0.0399(8)	0.0264(7)	0.0078(6)	0.0009(6)	0.0150(7)
C(10)	0.0309(7)	0.0252(7)	0.0230(7)	-0.0024(5)	0.0027(5)	0.0129(6)
C(11)	0.0370(8)	0.0297(7)	0.0255(7)	0.0002(6)	-0.0009(6)	0.0143(6)
C(12)	0.0586(11)	0.0312(8)	0.0252(8)	0.0020(6)	0.0020(7)	0.0186(8)
C(13)	0.0571(11)	0.0279(8)	0.0331(8)	0.0025(6)	0.0177(7)	0.0119(8)
C(14)	0.0359(9)	0.0329(8)	0.0474(10)	-0.0019(7)	0.0132(7)	0.0097(7)
C(15)	0.0309(8)	0.0321(8)	0.0340(8)	-0.0026(6)	0.0016(6)	0.0125(6)
O(1)	0.0687(19)	0.0687(19)	0.037(2)	0.000	0.000	0.0343(10)
C(16)	0.160(5)	0.160(5)	0.030(3)	0.000	0.000	0.080(3)
C(17)	0.079(6)	0.122(8)	0.020(4)	0.002(4)	-0.002(4)	0.084(6)

*The exponent takes the form: $-2\pi^2\Sigma\Sigma U_{ij}h_ih_j\mathbf{a}_i^*\mathbf{a}_j^*$

Table 5. Coordinates and isotropic temperature factors* (Å^2) for H atoms

Atoms	*x*	*y*	*z*	$U_{eq.}$	*Occupancy*
H(1)	0.6330(11)	0.9747(11)	-0.0163(15)	0.049(5)	1.0000
H(3)	0.5281	0.8769	0.0596	0.032	1.0000
H(5)	0.7397	1.0773	-0.0328	0.035	1.0000
H(6)	0.8546	1.1693	-0.0567	0.042	1.0000
H(7)	0.9427	1.1911	0.0524	0.048	1.0000
H(8)	0.9154	1.1205	0.1868	0.051	1.0000
H(9)	0.8014	1.0275	0.2102	0.040	1.0000
H(11)	0.6298	0.8598	0.3356	0.038	1.0000
H(12)	0.5608	0.7821	0.4540	0.048	1.0000
H(13)	0.4383	0.7237	0.4441	0.052	1.0000
H(14)	0.3844	0.7449	0.3166	0.051	1.0000
H(15)	0.4524	0.8228	0.1978	0.040	1.0000
H(1A)	0.3595	0.6522	0.5258	0.087	1.6667
H(16A)	0.3807	0.6974	0.6553	0.140	1.6667
H(16B)	0.3174	0.6194	0.6553	0.140	1.6667
H(17A)	0.2843	0.6936	0.7385	0.088	1.6667
H(17B)	0.3009	0.7408	0.6474	0.088	1.6667
H(17C)	0.2372	0.6623	0.6474	0.088	1.6667

*The exponent takes the form: $-8\pi^2 U \sin^2\theta/\lambda^2$

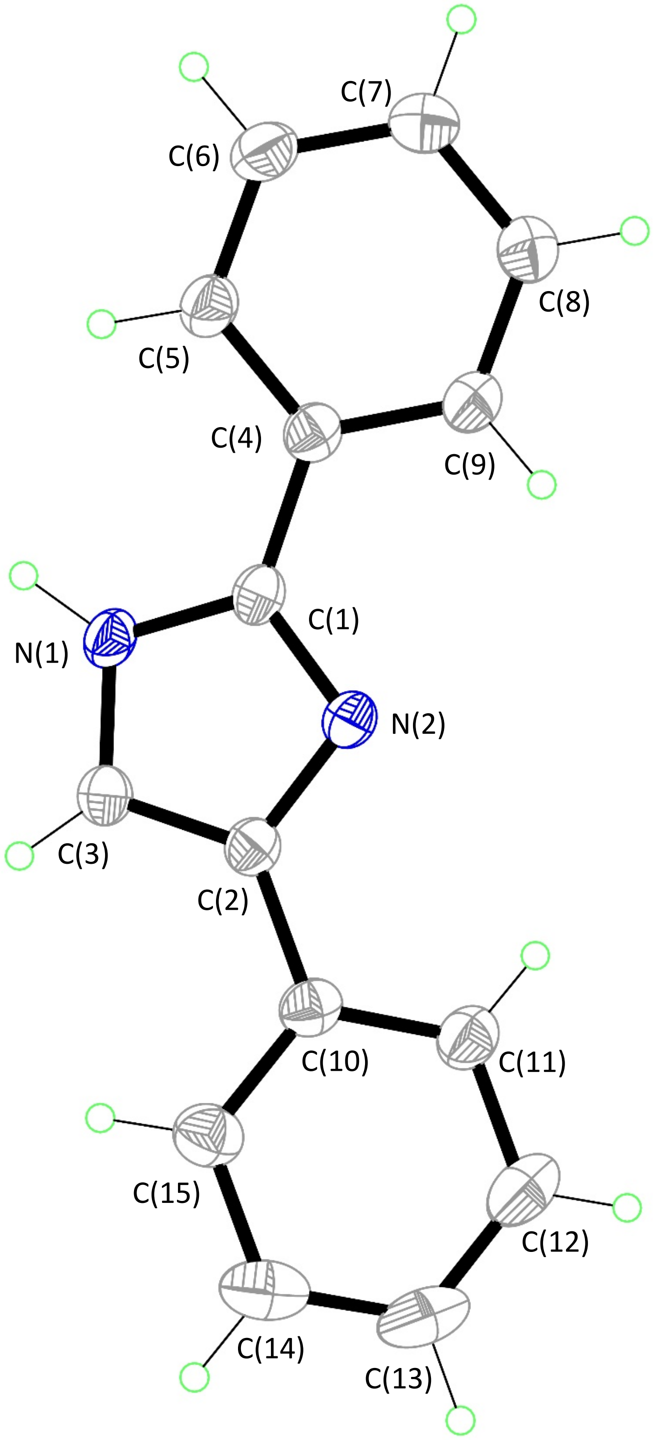

ORTEP drawing of $C_{15}H_{12}N_2$ with 50% probability ellipsoids, showing the atomic numbering scheme.

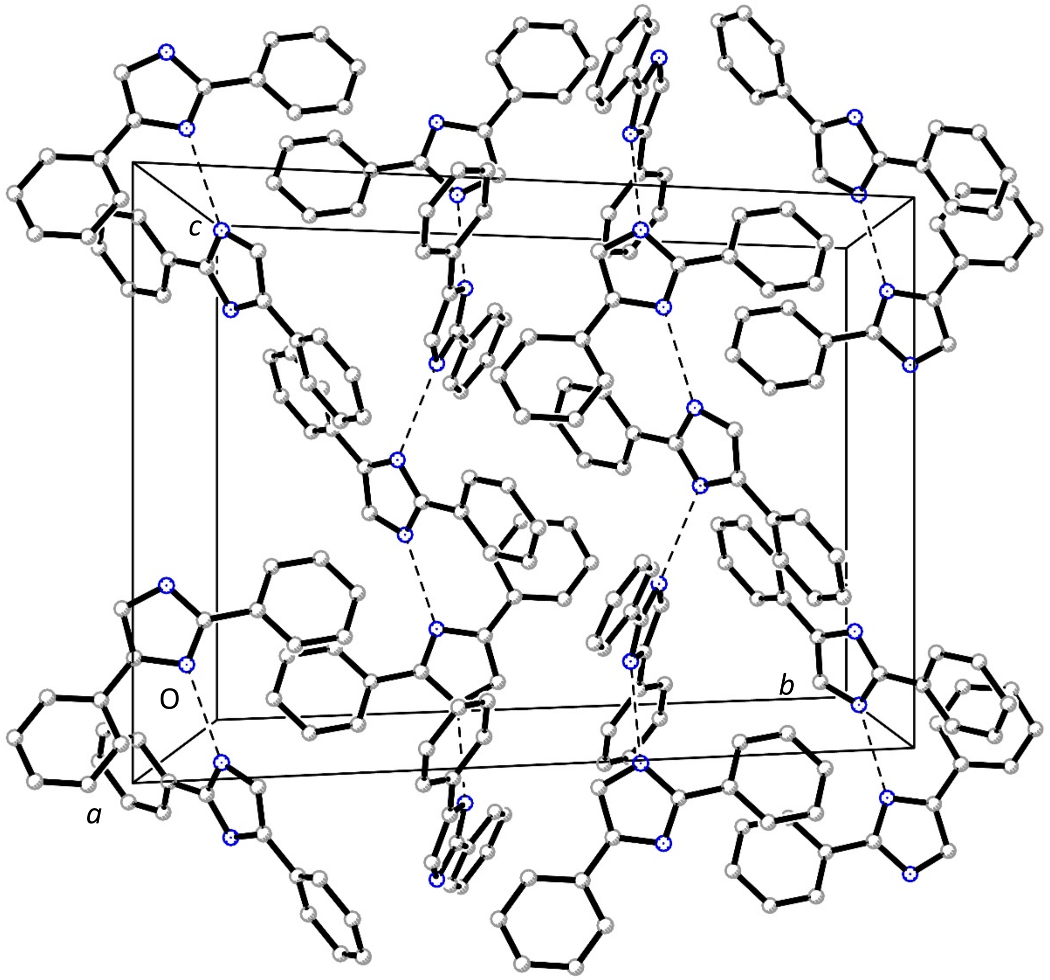

A packing view along the *a* direction

4. Computational predicted energies and cartesian coordinates

The optimization of structures of intermediates and transition states were performed by using Gaussian 16 quantum calculation software [10] with using M06-2X/6-31G(d,p) [11] with SMD implicit solvation model of DMSO with thermal correction at 373.15 K. The Gibbs free energies of systems were balanced by adding the free energies of H_2O and/or OH^-.

(1) H_2O

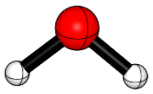

Name: H2O

Charge: 0

Multiplicity: 1

E(UM062X) = -76.3916202364 Ha

Zero Point Energies: -76.370190 Ha

Thermal Energies: -76.366631 Ha

Thermal Enthalpies: -76.365449 Ha

Thermal Free Energies: -76.393349 Ha

O	0.000000	0.000000	0.119270
H	0.000000	0.758044	-0.477080
H	-0.000000	-0.758044	-0.477080

(2) OH

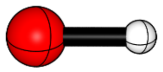

Name: OH

Charge: -1

Multiplicity: 1

E(UM062X) = -75.8110160484 Ha

Zero Point Energies: -75.802706 Ha

Thermal Energies: -75.799752 Ha

Thermal Enthalpies: -75.798570 Ha

Thermal Free Energies: -75.823999 Ha

O	0.000000	0.000000	0.108293
H	0.000000	0.000000	-0.866348

(3) R0-1

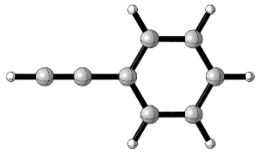

Name: R0-1

Charge: 0

Multiplicity: 1

E(UM062X) = -308.270513683 Ha

Zero Point Energies: -308.159806 Ha

Thermal Energies: -308.150117 Ha

Thermal Enthalpies: -308.148935 Ha

Thermal Free Energies: -308.200054 Ha

C	1.509964	-1.207294	0.000003
C	2.206848	0.000231	-0.000041
C	1.509598	1.207541	-0.000005
C	0.119167	1.212372	-0.000041
C	-0.585022	-0.000247	0.000058
C	0.119538	-1.212597	-0.000048
C	-2.022098	-0.000166	0.000322
C	-3.230183	0.000058	-0.000190
H	2.050713	-2.148221	0.000021
H	3.292268	0.000389	-0.000089
H	2.050043	2.148644	0.000012
H	-0.429946	2.148383	0.000015
H	-0.429245	-2.148801	0.000002
H	-4.300709	0.000212	-0.000300

(4) R0-2

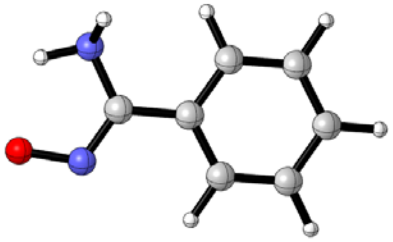

Name: R0-2

Charge: -1

Multiplicity: 1

E(UM062X) = -455.553374450 Ha

Zero Point Energies: -455.421383 Ha

Thermal Energies: -455.408879 Ha

Thermal Enthalpies: -455.407698 Ha

Thermal Free Energies: -455.465796 Ha

C	0.150757	0.045565	-0.010135
C	-1.303638	0.167783	-0.006212
N	-1.899278	1.458819	0.010356
N	-2.061042	-0.899929	-0.065912
O	-3.352899	-0.685561	-0.039494
C	0.791855	-1.210345	0.049637
C	2.174939	-1.309667	0.052140
C	2.974964	-0.163046	0.001935
C	2.358063	1.083567	-0.056616
C	0.968423	1.189578	-0.064986
H	-1.593562	1.999874	0.816572
H	-2.894407	1.235484	0.117097
H	0.177865	-2.103626	0.095330
H	2.639009	-2.291178	0.098582
H	4.057220	-0.244906	0.009368
H	2.961460	1.986302	-0.099558
H	0.505660	2.169704	-0.127117

(5) R1-R2-TS1

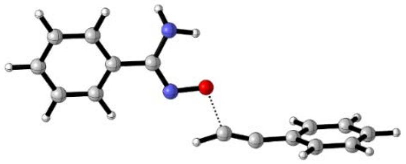

Name: R1-R2-TS1

Charge: -1

Multiplicity: 1

E(UM062X) = -763.814817112 Ha

Zero Point Energies: -763.570963 Ha

Thermal Energies: -763.547322 Ha

Thermal Enthalpies: -763.546140 Ha

Thermal Free Energies: -763.634061 Ha

Imaginary Frequencies: -281.7006 cm-1

C	5.870997	-0.333969	-1.125359
C	6.504035	-0.441763	0.113614
C	5.803888	-0.072678	1.262952
C	4.498055	0.395165	1.181392
C	3.841939	0.506029	-0.064860
C	4.565369	0.132166	-1.219409
C	2.513395	1.001783	-0.156402
C	1.278433	1.091982	-0.205151
H	6.400264	-0.616762	-2.031197
H	7.524212	-0.805205	0.182086
H	6.280530	-0.149521	2.236329
H	3.965559	0.681156	2.083649
H	4.085058	0.212304	-2.190109
H	0.396356	1.708592	-0.294767
C	-3.514557	-0.212971	0.021978
C	-2.125266	-0.712541	0.040006
N	-1.911010	-2.096296	0.167426
N	-1.148043	0.116178	-0.125399
O	0.070690	-0.435294	-0.084556
C	-3.787003	1.162734	0.101453
C	-5.094250	1.629661	0.081280
C	-6.164069	0.735835	-0.010285
C	-5.905880	-0.629468	-0.087458
C	-4.594041	-1.101361	-0.074309
H	-2.472084	-2.514607	0.903009
H	-0.918500	-2.228724	0.349578
H	-2.955833	1.855422	0.180834
H	-5.283252	2.697339	0.144308
H	-7.185551	1.102921	-0.020039
H	-6.727600	-1.335541	-0.163316
H	-4.407589	-2.167818	-0.154767

(6) R1

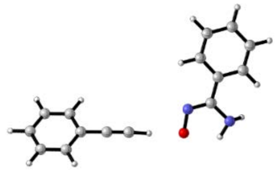

Name: R1

Charge: -1

Multiplicity: 1

E(UM062X) = -763.838442992 Ha

Zero Point Energies: -763.595097 Ha

Thermal Energies: -763.573439 Ha

Thermal Enthalpies: -763.572257 Ha

Thermal Free Energies: -763.653330 Ha

Imaginary Frequencies: -41.1518 cm-1

C	-6.560166	-0.178956	0.800221
C	-6.876618	0.962670	0.068813
C	-5.901658	1.586469	-0.707248
C	-4.612802	1.071780	-0.757612
C	-4.291645	-0.079753	-0.029460
C	-5.273501	-0.698236	0.756095
C	-2.954468	-0.603760	-0.062478
C	-1.835471	-1.053487	-0.093922
H	-7.313154	-0.666313	1.410582
H	-7.879713	1.366837	0.106379
H	-6.141250	2.477404	-1.275181
H	-3.848850	1.556489	-1.356559
H	-5.020371	-1.582789	1.328494
H	-0.832705	-1.509101	-0.160371
C	3.811539	-0.023144	0.019266
C	2.931912	-1.191348	-0.112340
N	3.507577	-2.477285	-0.262021
N	1.639667	-1.051477	-0.044277
O	0.949706	-2.172855	-0.172183
C	3.292089	1.282358	0.074081
C	4.130377	2.380693	0.191696
C	5.516269	2.216124	0.251614
C	6.043857	0.931096	0.196077
C	5.204266	-0.175291	0.084211
H	4.126694	-2.523889	-1.066946
H	2.697466	-3.077516	-0.432211
H	2.218145	1.413579	0.020461
H	3.702796	3.376789	0.231615
H	6.169078	3.076943	0.339671
H	7.117563	0.783296	0.243283
H	5.632079	-1.170840	0.058255

(7) R2

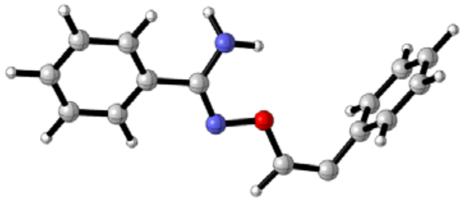

Name: R2

Charge: -1

Multiplicity: 1

E(UM062X) = -763.841119897 Ha

Zero Point Energies: -763.594652 Ha

Thermal Energies: -763.571243 Ha

Thermal Enthalpies: -763.570062 Ha

Thermal Free Energies: -763.657552 Ha

C	-2.954652	-0.206213	0.039466
C	-1.469549	-0.225890	0.042462
N	-0.836256	-1.438110	0.197720
N	-0.854882	0.887110	-0.170960
O	0.521987	0.693163	-0.118916
C	-3.645192	0.979235	0.319435
C	-5.034771	1.001380	0.305166
C	-5.753382	-0.159680	0.018133
C	-5.072020	-1.341904	-0.258714
C	-3.679216	-1.367068	-0.249925
H	-1.312720	-2.109103	0.786245
H	0.154122	-1.354691	0.390208
H	-3.081712	1.876262	0.553589
H	-5.559928	1.925182	0.527180
H	-6.838729	-0.141462	0.011999
H	-5.623368	-2.248243	-0.488758
H	-3.156804	-2.288641	-0.487907
C	4.667650	-1.070979	-1.044308
C	5.100301	-1.445015	0.229881
C	4.666230	-0.692938	1.323682
C	3.824431	0.399994	1.150173
C	3.366058	0.800545	-0.129756
C	3.826320	0.021620	-1.220720
C	2.557115	1.989825	-0.321433
C	1.236130	1.910036	-0.313122
H	4.988748	-1.641057	-1.913167
H	5.760676	-2.295497	0.366155
H	4.986084	-0.964208	2.327264
H	3.502781	0.972313	2.017601
H	3.505733	0.295683	-2.223466
H	0.524473	2.727456	-0.445454

(8) R3

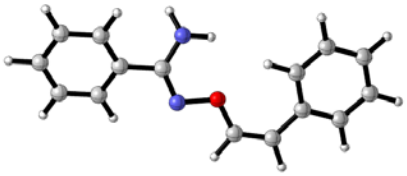

Name: R3

Charge: 0

Multiplicity: 1

E(UM062X) = -764.402867457 Ha

Zero Point Energies: -764.141798 Ha

Thermal Energies: -764.118515 Ha

Thermal Enthalpies: -764.117334 Ha

Thermal Free Energies: -764.202935 Ha

C	-3.075172	-0.170126	0.059639
C	-1.590731	-0.243225	0.087351
N	-1.007432	-1.446069	0.341601
N	-0.952021	0.845222	-0.196278
O	0.436590	0.592570	-0.121321
C	-3.723449	1.036416	0.344122
C	-5.111515	1.106323	0.308933
C	-5.863136	-0.025245	-0.006789
C	-5.220379	-1.227614	-0.291103
C	-3.830392	-1.302537	-0.259601
H	-1.552533	-2.120004	0.861963
H	-0.019429	-1.434593	0.557468
H	-3.134084	1.910302	0.600706
H	-5.608517	2.044024	0.536279
H	-6.946873	0.030911	-0.030131
H	-5.799715	-2.109811	-0.543891
H	-3.335218	-2.237886	-0.502520
C	4.012482	-1.723149	-0.122386
C	5.342424	-1.416029	0.158879
C	5.721528	-0.080734	0.289047
C	4.777501	0.929875	0.146358
C	3.429651	0.637106	-0.121876
C	3.064186	-0.713585	-0.262696
C	1.153457	1.733753	-0.259630
H	3.708167	-2.759127	-0.238862
H	6.076444	-2.208000	0.269454
H	6.755580	0.174566	0.500430
H	5.079528	1.969189	0.246694
H	2.040784	-0.974501	-0.505585
H	0.552569	2.632778	-0.358792
C	2.492307	1.758801	-0.254375
H	2.934183	2.747353	-0.335151

(9) R4-R5-TS2

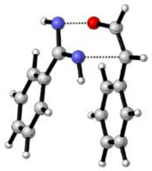

Name: R4-R5-TS2

Charge: 0

Multiplicity: 1

E(UM062X) = -764.338111602 Ha

Zero Point Energies: -764.078442 Ha

Thermal Energies: -764.056580 Ha

Thermal Enthalpies: -764.055398 Ha

Thermal Free Energies: -764.134329 Ha

Imaginary Frequencies: -515.0087 cm-1

C	0.390525	-1.409929	0.479835
C	-1.080834	-1.441727	0.785321
N	-1.667962	-0.661399	1.662085
N	-1.958189	-2.154970	0.036621
O	-2.448026	-0.835389	-1.182915
C	0.894024	-1.899635	-0.731664
C	2.253100	-1.824561	-1.018926
C	3.134406	-1.255263	-0.101950
C	2.646100	-0.773444	1.110473
C	1.287265	-0.857053	1.401229
H	-0.988761	-0.029917	2.095493
H	0.230801	-2.312974	-1.485159
H	2.621111	-2.200711	-1.968301
H	4.193803	-1.190608	-0.329173
H	3.323692	-0.335223	1.836537
H	0.933546	-0.495656	2.362830
C	0.966937	2.869689	0.600365
C	1.697976	2.442829	-0.508983
C	1.103135	1.603734	-1.450123
C	-0.207309	1.168689	-1.278465
C	-0.943266	1.572562	-0.154439
C	-0.345626	2.447190	0.768409
C	-2.308367	1.115533	0.118659
C	-3.010637	0.089954	-0.489831
H	1.420107	3.532823	1.330434
H	2.724485	2.769814	-0.643308
H	1.665743	1.278852	-2.320153
H	-0.662352	0.503807	-2.003245
H	-0.917887	2.774987	1.632748
H	-4.064136	-0.033221	-0.217149
H	-2.892440	1.760081	0.773888
H	-1.485016	-2.715768	-0.672514

(10) R4

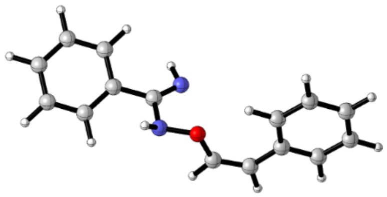

Name: R4

Charge: 0

Multiplicity: 1

E(RM062X) = -764.382668493 Ha

Zero Point Energies: -764.121075 Ha

Thermal Energies: -764.098140 Ha

Thermal Enthalpies: -764.096958 Ha

Thermal Free Energies: -764.183446 Ha

C	3.036903	0.146624	0.140213
C	1.556579	0.064169	0.324282
N	0.864850	0.589698	1.264650
N	0.942082	-0.753812	-0.636503
O	-0.449965	-0.635237	-0.668405
C	3.758749	-0.920334	-0.405693
C	5.139361	-0.822360	-0.556743
C	5.805110	0.338945	-0.171222
C	5.087749	1.405740	0.368512
C	3.709116	1.311379	0.524056
H	1.487531	1.058894	1.923399
H	3.244491	-1.832008	-0.692928
H	5.694952	-1.657108	-0.971819
H	6.881235	0.413893	-0.291680
H	5.601820	2.315064	0.662856
H	3.149473	2.149550	0.929490
C	-5.636845	0.258754	0.473695
C	-5.404890	1.343896	-0.369972
C	-4.152236	1.492096	-0.962678
C	-3.136514	0.571786	-0.719308
C	-3.354639	-0.524098	0.133953
C	-4.624503	-0.661742	0.719542
C	-2.338952	-1.533133	0.457955
C	-1.043277	-1.570721	0.129482
H	-6.608142	0.128340	0.941254
H	-6.192436	2.064967	-0.565043
H	-3.961816	2.331804	-1.624526
H	-2.171280	0.704931	-1.191918
H	-4.811300	-1.505603	1.378662
H	-0.380052	-2.367114	0.454031
H	-2.674768	-2.366795	1.067690
H	1.281225	-0.614126	-1.585699

(11) R5-R6-TS3

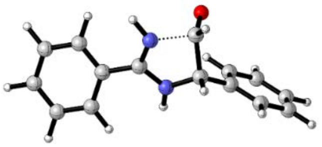

Name: R5-R6-TS3

Charge: 0

Multiplicity: 1

E(UM062X) = -764.443082587 Ha

Zero Point Energies: -764.181916 Ha

Thermal Energies: -764.160151 Ha

Thermal Enthalpies: -764.158969 Ha

Thermal Free Energies: -764.239607 Ha

Imaginary Frequencies: -127.8076 cm-1

C	2.467499	-0.017269	-0.171848
C	1.138320	0.573975	-0.437466
N	0.058749	-0.192825	-0.611955
N	0.854713	1.844208	-0.521577
O	-1.062948	2.161863	1.028000
C	3.408320	0.693265	0.579849
C	4.656298	0.135422	0.833847
C	4.972490	-1.127354	0.334871
C	4.037538	-1.834823	-0.418248
C	2.785566	-1.284068	-0.671966
H	0.059718	-1.196291	-0.487779
H	3.159350	1.670825	0.981496
H	5.381286	0.685762	1.424421
H	5.948407	-1.559388	0.532370
H	4.283900	-2.814848	-0.813342
H	2.066611	-1.830313	-1.275303
C	-4.651530	-0.843815	-0.626842
C	-4.687244	-1.261226	0.701347
C	-3.568376	-1.079346	1.515474
C	-2.419659	-0.484042	1.003664
C	-2.375750	-0.069271	-0.332307
C	-3.498181	-0.252318	-1.140908
C	-1.141364	0.590018	-0.871114
C	-0.840317	2.002465	-0.193699
H	-5.517793	-0.984011	-1.266175
H	-5.582130	-1.727363	1.102272
H	-3.594915	-1.399784	2.552707
H	-1.552003	-0.311655	1.631473
H	-3.466339	0.067304	-2.179375
H	-1.097501	2.808674	-0.915698
H	-1.233727	0.757589	-1.952635
H	1.552839	2.529233	-0.251633

(12) R5

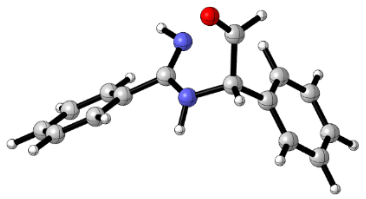

Name: R5

Charge: 0

Multiplicity: 1

E(RM062X) = -764.453480223 Ha

Zero Point Energies: -764.191856 Ha

Thermal Energies: -764.168775 Ha

Thermal Enthalpies: -764.167594 Ha

Thermal Free Energies: -764.252181 Ha

C	2.212315	-0.081042	0.131969
C	0.851444	0.476962	0.410194
N	0.124519	0.809940	-0.700970
N	0.364447	0.685791	1.580478
O	-0.704288	3.403603	0.286273
C	2.949500	0.333247	-0.981921
C	4.220803	-0.185907	-1.211835
C	4.761170	-1.126466	-0.337503
C	4.027980	-1.545430	0.771736
C	2.759960	-1.023909	1.007034
H	0.371339	0.355103	-1.570142
H	2.537934	1.077616	-1.656955
H	4.790699	0.148541	-2.072849
H	5.751236	-1.532549	-0.519549
H	4.441637	-2.283358	1.451647
H	2.183353	-1.361868	1.863398
C	-3.735667	-1.571916	-1.246828
C	-4.025227	-2.031796	0.036289
C	-3.425507	-1.424606	1.137497
C	-2.536865	-0.365082	0.962567
C	-2.239838	0.103038	-0.322782
C	-2.852582	-0.509622	-1.420456
C	-1.262925	1.237676	-0.583626
C	-1.370139	2.404645	0.403253
H	-4.201364	-2.035086	-2.111315
H	-4.716857	-2.856664	0.177326
H	-3.648634	-1.776826	2.140097
H	-2.059180	0.091427	1.822037
H	-2.634379	-0.145936	-2.421868
H	-2.154284	2.319288	1.178776
H	-1.520185	1.676554	-1.555273
H	1.063694	0.486060	2.294394

(13) R6

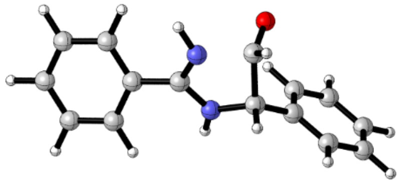

Name: R6

Charge: 0

Multiplicity: 1

E(UM062X) = -764.443106346 Ha

Zero Point Energies: -764.181503 Ha

Thermal Energies: -764.158944 Ha

Thermal Enthalpies: -764.157763 Ha

Thermal Free Energies: -764.240250 Ha

C	2.464650	-0.023107	-0.167597
C	1.129972	0.549042	-0.437391
N	0.058520	-0.221967	-0.622026
N	0.831596	1.819174	-0.520026
O	-1.059730	2.172167	0.984781
C	3.394295	0.699833	0.586286
C	4.647800	0.156772	0.844663
C	4.980002	-1.102934	0.348367
C	4.055838	-1.822616	-0.406575
C	2.798529	-1.286921	-0.665173
H	0.064691	-1.226375	-0.503686
H	3.132883	1.674603	0.986648
H	5.364548	0.716217	1.436663
H	5.960323	-1.523172	0.549414
H	4.314901	-2.800294	-0.799235
H	2.087667	-1.842124	-1.269890
C	-4.654683	-0.851864	-0.610057
C	-4.690998	-1.234185	0.728691
C	-3.571182	-1.034275	1.537361
C	-2.420751	-0.456298	1.009735
C	-2.376034	-0.077164	-0.336859
C	-3.499597	-0.277815	-1.139829
C	-1.140141	0.561879	-0.892512
C	-0.809380	1.985886	-0.236055
H	-5.521666	-1.006283	-1.245156
H	-5.587116	-1.686965	1.142042
H	-3.598522	-1.326912	2.582804
H	-1.552491	-0.267710	1.631757
H	-3.467185	0.014092	-2.186460
H	-1.076171	2.774186	-0.976429
H	-1.233187	0.707238	-1.976929
H	1.508426	2.518309	-0.233742

(14) R7

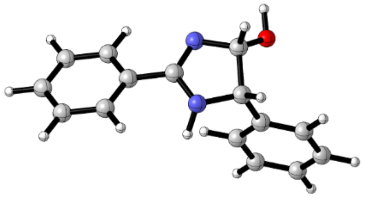

Name: R7

Charge: 0

Multiplicity: 1

E(UM062X) = -764.470586655 Ha

Zero Point Energies: -764.207105 Ha

Thermal Energies: -764.184928 Ha

Thermal Enthalpies: -764.183746 Ha

Thermal Free Energies: -764.266503 Ha

C	2.294149	0.143003	-0.107935
C	0.964445	0.773006	-0.266646
N	0.130073	0.376358	-1.299444
N	0.519731	1.707132	0.494479
C	2.985783	0.299711	1.097932
C	4.232747	-0.289503	1.267603
C	4.800085	-1.035836	0.234108
C	4.117225	-1.188436	-0.970269
C	2.866603	-0.600977	-1.143567
H	0.177428	-0.593680	-1.593858
H	2.532050	0.880225	1.894624
H	4.763110	-0.169384	2.206890
H	5.774177	-1.495785	0.368105
H	4.559172	-1.761733	-1.778801
H	2.348330	-0.707465	-2.091748
C	-4.452981	-0.820394	-0.138880
C	-4.014695	-1.889254	0.641556
C	-2.652043	-2.053963	0.880435
C	-1.731525	-1.155472	0.344105
C	-2.163929	-0.082777	-0.438440
C	-3.531322	0.074466	-0.675108
C	-1.195512	0.929679	-1.015737
C	-0.802453	2.065196	-0.016895
H	-5.512584	-0.687596	-0.335330
H	-4.730439	-2.591743	1.057139
H	-2.302334	-2.885002	1.485458
H	-0.671198	-1.299064	0.534807
H	-3.872633	0.905167	-1.287842
H	-0.704590	3.011616	-0.569063
H	-1.635711	1.366093	-1.915385
O	-1.761285	2.188655	0.993702
H	-1.482589	2.923990	1.556561

(15) R8

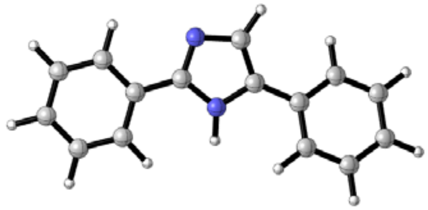

Name: R8

Charge: 0

Multiplicity: 1

E(RM062X) = -688.092966194 Ha

Zero Point Energies: -687.856821 Ha

Thermal Energies: -687.837130 Ha

Thermal Enthalpies: -687.835948 Ha

Thermal Free Energies: -687.913025 Ha

C	2.480934	-0.075372	0.007916
C	1.101118	-0.574491	0.080179
N	-0.004054	0.212155	-0.020728
N	0.758766	-1.842120	0.247815
C	3.533740	-0.999442	-0.005155
C	4.850988	-0.561950	-0.071481
C	5.136652	0.802060	-0.127920
C	4.093525	1.725401	-0.114343
C	2.772631	1.292682	-0.045180
H	-0.015307	1.206298	-0.214016
H	3.304329	-2.059217	0.035459
H	5.658091	-1.287817	-0.081566
H	6.165736	1.142758	-0.180970
H	4.306370	2.788983	-0.154268
H	1.976102	2.030412	-0.024889
C	-4.848253	-0.531559	-0.339978
C	-5.143554	0.808139	-0.088743
C	-4.112209	1.694364	0.214373
C	-2.795319	1.247659	0.271342
C	-2.490569	-0.095643	0.014511
C	-3.533557	-0.980397	-0.295097
C	-1.114971	-0.592454	0.074821
C	-0.606833	-1.863352	0.245028
H	-5.644698	-1.228466	-0.581627
H	-6.170004	1.158142	-0.129927
H	-4.332267	2.738050	0.416031
H	-2.005877	1.946375	0.533767
H	-3.307333	-2.019930	-0.513650
H	-1.164062	-2.779681	0.384411

References

(1) Chen, X. Y.; Englert, U.; Bolm, C. *Chem. Eur. J.* **2015**, *38*, 13221 – 13224.

(2) Shi, S.; Xu, K.; Jiang, C.; Ding, Z. *J. Org. Chem.* **2018**, *83*, 14791 – 14796.

(3) Reddy, N. N. K.; Rao, S. N.; Ravi, C.; Adimurthy, S. *ACS Omega* **2017**, *2*, 5235 – 5241.

(4) Bipin, K. V.; Sunil, K.; Umesh, K.; Savita, P.; Priti, A. *Univ. J. Pharm. Res.* **2017**, *2*, 21 – 27.

(5) Gopi, E.; Kumar, T.; Menna-Barreto, R. F. S.; Valença, W. O.; da Silva Júnior, E. N.; Namboothiri, I. N. N. *Org. Biomol. Chem*. **2015**, *13*, 9862 – 9871.

(6) Huang, Y. Q.; Song, H. J.; Liu, Y. X.; Wang, Q. M. *Chem. Eur. J*. **2018**, *24*, 2065 – 2069.

(7) Pews-Davtyan, A.; Beller, M. *Chem. Comm*. **2011**, *47*, 2152 – 2154.

(8) Bellina, F.; Cauteruccio, S.; Rossi, R. *J. Org. Chem*. **2007**, *72*, 8543 – 8546.

(9) Fang, S.; Yu, H.; Yang, X.; Li, J.; Shao, L. *Adv. Syn. Catal.* **2019**, *361*, 3312 – 3317.

(10) Gaussian 16, Revision B.01, Frisch, M. J.; Trucks, G. W.; Schlegel, H. B.; Scuseria, G. E.; Robb, M. A.; Cheeseman, J. R.; Scalmani, G.; Barone, V.; Petersson, G. A.; Nakatsuji, H.; Li, X.; Caricato, M.; Marenich, A. V.; Bloino, J.; Janesko, B. G.; Gomperts, R.; Mennucci, B.; Hratchian, H. P.; Ortiz, J. V.; Izmaylov, A. F.; Sonnenberg, J. L.; Williams-Young, D.; Ding, F.; Lipparini, F.; Egidi, F.; Goings, J.; Peng, B.; Petrone, A.; Henderson, T.; Ranasinghe, D.; Zakrzewski, V. G.; Gao, J.; Rega, N.; Zheng, G.; Liang, W.; Hada, M.; Ehara, M.; Toyota, K.; Fukuda, R.; Hasegawa, J.; Ishida, M.; Nakajima, T.; Honda, Y.; Kitao, O.; Nakai, H.; Vreven, T.; Throssell, K.; Montgomery, J. A.; Peralta, Jr., J. E.; Ogliaro, F.; Bearpark, M. J.; Heyd, J. J.; Brothers, E. N.; Kudin, K. N.; Staroverov, V. N.; Keith, T. A.; Kobayashi, R.; Normand, J.; Raghavachari, K.; Rendell, A. P.; Burant, J. C.; Iyengar, S. S.; Tomasi, J.; Cossi, M.; Millam, J. M.; Klene, M.; Adamo, C.; Cammi, R.; Ochterski, J. W.; Martin, R. L.; Morokuma, K.; Farkas, O.; Foresman, J. B.; and Fox, D. J. Gaussian, Inc., Wallingford CT, **2016**.

(11) Zhao, Y.; Truhlar, D. G. *Theor. Chem. Acc.* **2008**, *120*, 215 – 241.
